# Comparative analysis of the organization and complexity of immunoglobulin light chain loci in equids

**DOI:** 10.1093/jas/skag001

**Published:** 2026-01-09

**Authors:** Yanbo Qiu, Yichen Lei, Xiaohua Yi, Xiaoqin Tang, Beibei Zhang, Shuhui Wang, Xiuzhu Sun

**Affiliations:** College of Grassland Agriculture, Northwest A&F University, Yangling, Shaanxi, 712100, China; College of Animal Science and Technology, Northwest A&F University, Yangling, Shaanxi, 712100, China; College of Animal Science and Technology, Northwest A&F University, Yangling, Shaanxi, 712100, China; College of Animal Science and Technology, Northwest A&F University, Yangling, Shaanxi, 712100, China; College of Grassland Agriculture, Northwest A&F University, Yangling, Shaanxi, 712100, China; College of Animal Science and Technology, Northwest A&F University, Yangling, Shaanxi, 712100, China; College of Grassland Agriculture, Northwest A&F University, Yangling, Shaanxi, 712100, China

**Keywords:** comparative analysis, donkey, horse, immunoglobulin, IGλ, IGκ

## Abstract

This study investigated three donkey breeds—Guanzhong, Jiami, and Northern Shaanxi—to characterize the structural organization and diversification mechanisms of the immunoglobulin light-chain (IgL) loci and to conduct both intra-breed and interspecies comparisons with horses. The donkey IGλ locus is located on chromosome 8 and arranged in a Vλ-(Jλ-Cλ)-Vλ configuration. It contains 7 Cλ genes, each preceded by a corresponding Jλ gene to form a Jλ-Cλ cluster. Upstream of this cluster, 156 Vλ genes were identified, including 29 potential functional genes; downstream, 98 Vλ genes were detected, of which 22 were potentially functional. The IGκ locus resides on chromosome 6 and exhibits a Vκ-Jκ-Cκ structure, comprising one Cκ gene, 5 Jκ genes, and 72 Vκ segments, including 22 potential functional Vκ genes. Expression profiling revealed clear light-chain usage preferences in donkeys. For the λ-chain, Vλ72, Vλ135, Vλ150, Vλ190, and Vλ196—mainly belonging to the IGLV1 and IGLV3 subgroups—were preferentially utilized. For the κ-chain, Vκ67 and Vκ71 were dominantly expressed, highlighting the prominent role of the IGκV4 subgroup. Horses showed a similar pattern, with strong biases toward the IGLV1, IGLV3, and IGκV4 subgroups. Although breed-specific differences were observed in certain IGL expression profiles (e.g. V-J combinations), these variations were largely restricted to the λ-chain and involved low-frequency genes. Thus, highly utilized genes and subgroups exhibit high conservation among donkey breeds. This study is the first to comprehensively elucidate the structure and expression preferences of the donkey IgL locus, laying a solid foundation for the development of donkey-derived antibody resources.

## Teaser

Three Chinese donkey breeds exhibit unique immunoglobulin gene diversification and similar gene expression patterns to horses, thus offering a robust cost-effective immune model for developing species-specific antibodies.

## Introduction

Horses and donkeys play vital roles in transportation, agricultural labor, and recreational activities. Their disease resistance relies on complex immune system mechanisms, where immunoglobulins (IG) expressed by B cells are crucial effector molecules in adaptive immunity. In vertebrates, the limited set of germline IG genes is expanded through mechanisms, such as V(D)J recombination, somatic hypermutation (SHM), and class switch recombination (CSR) to enhance IG diversity and enable responses to evolving pathogens.

In jawed vertebrates, genes encoding the variable regions (V-regions) of B and T lymphocyte antigen receptors are generated via somatic recombination of variable (V), diversity (D), and joining (J) gene segments (Tonegawa, 1983; Davis and Bjorkman, 1988). This process is initiated by lymphocyte-specific recombination activating genes 1 and 2 (RAG1/RAG2) endonucleases, which introduce double-strand breaks (DSBs) at the boundaries of V, D, and J gene segments (Schatz and Swanson, 2011). The subsequent recombination results in diverse V, D, and J gene combinations, forming complete exons for the variable regions, which are transcribed and translated into full variable region fragments.

It is noteworthy that to generate an effective immune repertoire, V(D)J recombination achieves genetic diversity of IG at two levels. The first level is the diversified rearrangement of V, D, and J genes. The second level involves a specific joining mechanism of the encoding segments, characterized by the loss or addition of nucleotides (Chi et al., 2020). During V(D)J recombination, terminal deoxynucleotidyl transferase (TdT) adds nontemplated nucleotides (N-nucleotides) at the V-D and D-J junctions of the immunoglobulin heavy chain (IGH), greatly enhancing the diversity of the immune repertoire (Ijspeert et al., 2016).

Following V(D)J recombination, B cells undergo SHM and CSR, both of which are genetic modifications. Mature B cells migrate to peripheral lymphoid tissues, and upon antigen stimulation, they move to germinal centers of secondary lymphoid organs, where point mutations are introduced into the variable region of IGs, or, less frequently, nucleotide insertions and deletions occur. This process, known as SHM, results in antibodies with higher affinity for the antigen (Papavasiliou and Schatz, 2002; Durandy and Kracker, 2012). B cells expressing these high-affinity antibodies are preferentially selected for proliferation and survival. The continuous cycle of mutation and selection generates B cells that produce high-affinity antibodies (Siskind and Benacerraf, 1969).

Initially, each B cell expresses IgM, and upon entering the periphery, it co-expresses IgM and IgD on the cell surface. Notably, IgD is not produced via CSR, but through selective splicing of the primary transcript encoding IgM. CSR is a region-specific recombination process in which the μ constant region (Cμ) in the IGH gene is replaced by downstream constant regions (Cγ, Cε, or Cα), resulting in the switch from the primary IgM/IgD isotype to IgG, IgE, or IgA (Xu et al., 2012). Both SHM and CSR require the involvement of activation-induced cytidine deaminase (AID) (Shinkura et al., 2004).

Current research has provided detailed insights into the structure and expression diversity of the horse IG locus, while studies on the same loci in donkeys, a closely related species, are still in their infancy. In 2010, Sun et al. identified the horse IGH locus on chromosome 24, containing 50 VH, 40 DH, and 8 JH segments; the immunoglobulin kappa chain (IGκ) locus, which includes 60 Vκ, 5 Jκ, and 1 Cκ gene segments; and the immunoglobulin lambda chain (IGλ) locus, containing 7 Cλ genes, each preceded by a Jλ gene, arranged in an alternating Jλ-Cλ sequence (Jλ1-Cλ1-Jλ2-Cλ2…-Jλ7-Cλ7). They also identified 110 Vλ genes with the same transcriptional polarity as Jλ-Cλ clusters upstream, while 34 Vλ segments downstream exhibited the opposite transcriptional polarity (Sun et al., 2010). In 2015, Walther et al. expanded the analysis and found 52 VH, 40 DH, 8 JH, and 11 CH genes in the IGH locus, including 7 γ-chain CH genes, with two additional VH genes compared to the previous study. The composition of the IGκ locus remained consistent with the findings of Sun et al. (2010). The IGλ locus also revealed 144 Vλ genes and standardized gene naming was further established (Walther et al., 2015).

Horses have been widely used in the production of antisera, such as antivenom, with the advantage that IG gene research is relatively mature (Sun et al., 2010; Walther et al., 2015; Ruschig et al., 2024), and the large size and blood volume of the horse make the unit cost of serum production much lower than that of smaller animals. However, horses are more expensive to raise, require more space and feed, and are relatively complex to manage. Donkeys, as animals of the genus *Equus*, are often regarded as mini-horses (Haines and Goliszek, 2019), and they are important in agricultural production, economic development, cultural heritage, and ecological protection. In this study, we used typical livestock resources from Shaanxi Province (Guanzhong, Jiami, and Northern Shaanxi donkeys and Guanzhong and Ningqiang horses) to elucidate the structural characteristics of donkey IGL loci and the heterogeneity of IGL gene expression patterns in donkey and horse breeds using genome comparison and high-throughput sequencing technologies. This study will provide theoretical references for the design of donkey vaccines and the development of donkey-derived antibodies.

## Materials and Methods

All experiments implemented were approved by the Institutional Animal Care and Use Committee of the Northwest A&F University (IACUC-NWAFU), and followed local animal welfare guidelines, laws, and policies.

### Experimental animals and RNA isolation

This study involved healthy adult male individuals of the Guanzhong, Jiami, and Northern Shaanxi donkey breeds, and the Guanzhong and Ningqiang horse breeds, from the following locations: the Guanzhong Donkey Breeding Center (Fufeng, Shaanxi), Jiami Donkey Breeding Center (Yulin, Shaanxi), Wuqi Donkey Cooperative (Yan’an, Shaanxi), Baoji Liulin Junyi Agricultural Development Co., Ltd. (Meixian, Shaanxi), and the Ningqiang Horse National Breeding Center (Hanzhong, Shaanxi). The animals are hereafter referred to as GZ Donkey, JM Donkey, NS Donkey, GZ Horse, and NQ Horse, respectively. All animals were maintained under standard housing and management ­conditions (10-25 °C, 50–70% humidity). They were kept in well-ventilated pens with dry bedding, provided with ad libitum access to clean water and a balanced diet formulated according to the nutritional requirements of adult equines. All animals were exposed to a natural light-dark cycle. Routine health monitoring and veterinary care were provided to ensure the well-being of all animals. Spleen tissues were collected from horses and donkeys and immediately stored in liquid nitrogen, with at least three biological replicates for each breed. RNA was extracted using an optimized Trizol-based method (Zhang et al., 2023).

### Analysis of the donkey IGL locus structure

The Vλ/Vκ, Jλ/Jκ, and Cλ/Cκ gene sequences from species such as horse, pig, dog, mouse, and human were retrieved from the NCBI database. BLAST (https://blast.ncbi.nlm.nih.gov/Blast.cgi) was used to locate V, J, and C genes within the donkey genome (GCA_016077325.2). The structure of the IG genes was determined using Ig BLAST (https://www.ncbi.nlm.nih.gov/igblast/) and FUZZNUC (http://www-archbac.u-psud.fr/genomics/patternSearch.html). Identified Vλ and Vκ genes were classified as “potentially functional Vλ/Vκ”, “open reading frames (ORFs)”, or “pseudogenes (pVλ or pVκ)” based on criteria (Lefranc, 1998). RSS sequences matching the 12/23 rule (with up to five base mismatches allowed) were searched for potential Jλ and Jκ genes. The V and J genes were named according to their types and genomic locations, and a structural map of the donkey immunoglobulin IGλ and IGκ loci was constructed (the corresponding gene sequences are provided in Supplementary Material A).

### Analysis of germline gene subgroup classification

All Vλ/Vκ gene segments were selected, and the nucleotide sequences of all Vλ/Vκ germline genes were subjected to multiple sequence alignment using the Clustal Omega tool (https://www.ebi.ac.uk/jdispatcher/msa/clustalo). Based on the alignment results, a percentage identity matrix was constructed to quantify the similarity between each pair of sequences. Using this matrix, we established the following criteria for gene subgroup classification: 1) Sequences with a nucleotide identity ≥75% were assigned to the same gene family; 2) Sequences with an identity ≤70% were classified into different gene families; 3) For sequences with an identity between 70% and 75%, the final classification was determined by comprehensively considering their sequence characteristics and evolutionary positions in the phylogenetic tree. The nomenclature of all subgroups is consistent with the subgroup numbers defined based on similarity in the IMGT database.

### 5′-RACE-Ready cDNA synthesis and rapid amplification of cDNA ends (RACE)

5′-RACE-Ready cDNA synthesis and RACE amplification were performed on RNA from nine donkeys and six horses using the SMARTer RACE 5′/3′ Kit (Takara, Dalian). The RACE amplification system (total volume 25  μL) consisted of: 12.5 μL 2 × Phanta Max Master Mix (Vazyme, Nanjing); 9  μL PCR-Grade H_2_O; 1  μL cDNA; 1.5  μL Universal Primer (UPM, 10 μM); and 1  μL downstream gene-specific primer (GSP, 10 μM). The following PCR program was used for amplification of the IG gene library: 95 °C for 3 min for template denaturation, followed by 30 cycles of 95 °C for 15 s, 60 °C for 15 s, and 72 °C for 1 min, with a final 5 min extension at 72 °C. A single upstream UPM primer (5′-AAGCAGTGGTATCAACGCAGAGT-3′) was used for donkeys and horses, whereas four downstream GSP primers were used for donkeys and horses, namely: Donkey-IGλ-R (5′-GCGGGAAGAGAGAGACCGAGGGT-3′), Donkey-IGκ-R (5′-GATGAAGGCAGATGGCTTAGCA-3′), Horse-IGλ-R (5′-GAGAGAGACCGAGGGTGCAGAC-3′) and Horse-IGκ-R (5′-AGATGAAGGCAGATGGCTTAGCAT-3′). PCR products were evaluated for size via electrophoresis on a 1.5% agarose gel.

### DNA library construction and High-Throughput sequencing

Specific amplification products were sent to Sangon Biotech (Shanghai) for library construction and sequencing. IG sequences were amplified in a two-step PCR process compatible with Illumina sequencing library preparation. The first-round PCR system included: 2 μL DNA template (10 ng/μL), 1 μL each of upstream and downstream primers (10 μM), and 15 μL 2 ×  PCR Ready Mix (Kapa HiFi Ready Mix) in a total volume of 25 μL. The PCR program was as follows: 98 °C for 3 min, followed by 8 cycles of 98 °C for 30 s, 60 °C for 30 s, and 72 °C for 30 s, with a final extension at 72 °C for 5 min. PCR products were analyzed on a 2% agarose gel, and the correct-sized bands were purified using AMPure XP magnetic beads. The second-round PCR, using the first-round products as templates, was performed to generate a sequencing library with molecular tags. The reaction system included: 2 μL DNA template (10 ng/μL), 1 μL each of P7 and P5 primers (containing molecular tags, 10 μM), and 15 μL 2 × PCR Ready Mix (total volume 30 μL). The PCR program was as follows: 98 °C for 5 min, followed by 5 cycles of 94 °C for 30 s, 55 °C for 20 s, and 72 °C for 30 s, with a final 72 °C extension for 5 min. PCR products were purified using AMPure XP beads. Sequencing was performed on the NovaSeq6000/MiSeq (Illumina, San Diego, CA) with dual-end 300 bp sequencing.

### Bioinformatics analysis of IGL genes

After high-throughput sequencing, the reads were assembled and filtered to remove sequences lacking amplification primers and those with a length shorter than 400 bp. The resulting data were processed in a Linux environment. First, IgBLAST from NCBI was downloaded and installed. Then, gene libraries for donkey Vλ/Vκ and Jλ/Jκ were constructed according to the IMGT database format requirements, as detailed in https://ncbi.github.io/igblast/cook/How-to-set-up.html. Sequences that aligned with both V and J gene segments were retained, and the matched sequences were assigned to germline genes for further analysis. The sequencing data generated and processed by PE300 are provided in Supplementary Table S1. Since the Vκ and Jκ sequences for horses are available in the IMGT database (https://www.imgt.org/), these were analyzed using the “NGS High-Throughput analysis” function. For horse Vλ and Jλ gene libraries, genes from published studies (Sun et al., 2010; Walther et al., 2015) were used, with the same analysis method applied as for donkeys.

The expression patterns of IGλ and IGκ genes in donkeys and horses were analyzed as follows: (i) V(D)J recombination analysis for each sample; (ii) Junctional diversity analysis, focusing on the contributions of N/P nucleotide insertions and the nucleotide lengths of 3'V-Region and 5'J-Region (Lefranc and Lefranc, 2020); (iii) CDR3 length distribution and the contribution of CDR3 diversity to IG diversity; (iv) SHM analysis, including categorizing observed mutations and calculating mutation frequencies from A, T, C, and G to the other three bases. The SHM frequency was calculated using the formula: SHM frequency = number of observed mutations/(number of bases in one Vλ/Vκ gene × number of observed genes). Mutations in the variable regions (FR1-FR3) were also analyzed.

### Statistical analysis

Data extraction and integration were performed using Microsoft Excel 2016 and PyCharm (v2020.1.1 x64). Multiple sequence alignment of the donkey Vλ and Vκ gene sequences was conducted with the ClustalW algorithm implemented in MEGA 11.0. The intrafamilial phylogenetic analysis for each gene was performed using the Maximum Parsimony (MP) method. The resulting phylogenetic trees were visualized using the iTOL online platform (https://itol.embl.de).

Diversity indices, including richness (S), the Shannon diversity index (H′), and Pielou’s evenness index (J), were calculated using R software (v4.4.1). During data preprocessing, non-functional VJ combinations (empty or with zero abundance across all replicates) were filtered out. The calculation criteria were as follows: Richness (S): The number of unique, productive VJ combinations (abundance > 0) within a single replicate. Shannon diversity index (H′): Calculated using the diversity function (index = “shannon”) from the vegan R package, reflecting both the richness and abundance distribution of VJ combinations. Pielou’s evenness index (J): Calculated as J = H′/ln(S), where ln(S) represents the theoretical maximum of H′ for a given richness S. The index was set to 0 when S = 0 to avoid calculating the logarithm of zero. Additionally, the correlation between CDR3 length and the number of cysteine residues was assessed using Spearman’s rank correlation analysis via the cor.test function in R (v4.4.1).

All statistical analyses were performed in R (v4.4.1). For multiple group comparisons, data normality and homogeneity of variances were first assessed using the Shapiro–Wilk test and Levene’s test, respectively. If both assumptions were met, one-way ANOVA followed by Tukey’s Honestly Significant Difference (HSD) post-hoc test was applied for group comparisons. If the assumptions were violated, the non-parametric Kruskal–Wallis test was used, followed by Dunn’s test for pairwise comparisons with *P*-value adjustment using the Benjamini-Hochberg (BH) method to control the false discovery rate. For comparisons between two groups, the independent-samples *t*-test was used for parametric data, and the Mann–Whitney *U*-test was used for non-parametric data. All tests were two-tailed, and statistical significance was set at *P *< 0.05. Corrected *P*-values (*P*adj) were reported, with *P *< 0.05 considered significant (*) and *P *< 0.01 considered highly significant (**). Data are presented as mean ± standard error of the mean (SEM).

## Results

### Composition of the donkey IGλ locus

**Figure 1.**
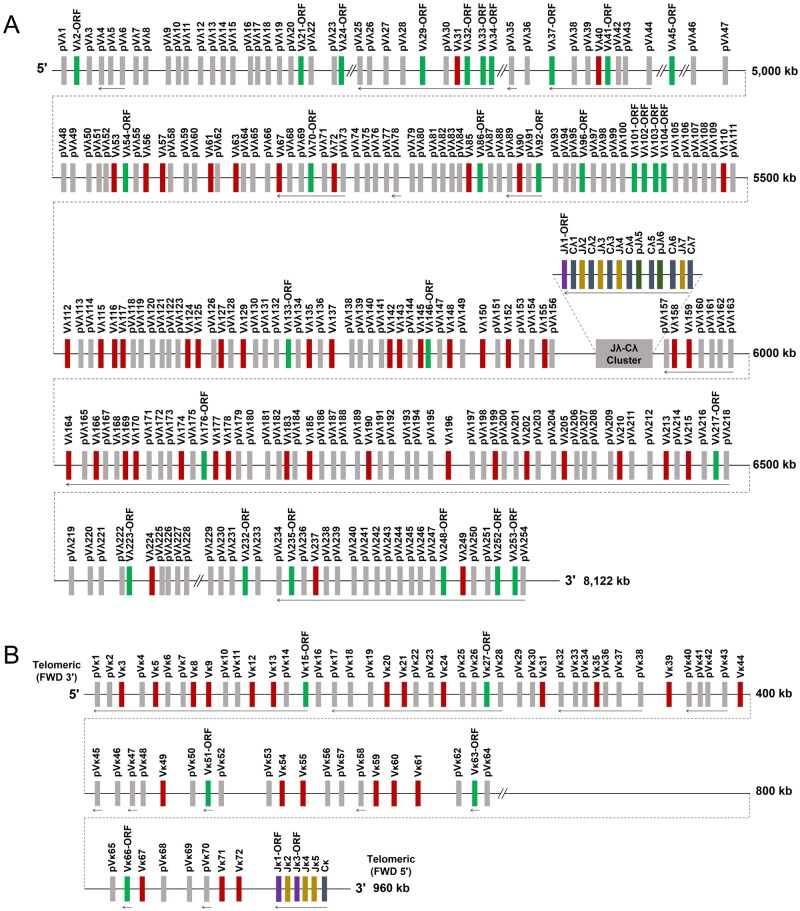
Schematic structure of IGλ (A) and IGκ (B) loci in donkeys.

The donkey IGλ locus is organized on chromosome 8 in the form of Vλ-(Jλ-Cλ)-Vλ. It comprises 7 Cλ genes, each preceded by a Jλ gene segment, forming Jλ-Cλ gene clusters. Upstream of these clusters, 156 Vλ genes were identified, including 29 potentially functional genes, 21 ORFs, and 106 pVλ. Approximately 22% (34) of the Vλ genes shared reverse transcriptional polarity with the Jλ-Cλ genes. Downstream of the Jλ-Cλ clusters, 98 Vλ genes were identified, including 22 potentially functional genes, 8 ORFs, and 68 pVλ. About 95% (93) of these downstream Vλ genes exhibited the same reverse transcriptional polarity as the Jλ-Cλ genes (Figure 1A). 254 Vλ genes were visualized in a phylogenetic tree (Supplementary Figure S1A). According to the subgroup classification criteria, 51 of these 254 Vλ genes—those that were potentially functional—were assigned to seven subgroups (IGLV1-IGLV6 and IGLV8) (Table 1). Notably, the present study identified a total of 254 Vλ genes in donkeys, with at least 174 being pVλ. Pseudogenes that were too short or truncated and thus could not be assigned to corresponding subgroups were excluded from the count in this study; consequently, the actual number of pVλ in donkeys may be higher than the estimate reported herein.

**Table 1.** Subgroup classification of Vλ genes in donkey

Subgroup	Total	Potential functional (% of total)	Pseudogene % of total)	ORF (% of total)	Vλ gene segments
**IGLV1**	31	15 (48.39)	14 (45.16)	2 (6.45)	Vλ31, Vλ40, Vλ56, Vλ63, Vλ72, Vλ90, Vλ110, Vλ117, Vλ124, Vλ129, Vλ135, Vλ143, Vλ148, Vλ150, Vλ237, Vλ2-ORF, Vλ33-ORF, pVλ6, pVλ13, pVλ19, pVλ22, pVλ65, pVλ74, pVλ88, pVλ99, pVλ113, pVλ140, pVλ154, pVλ230, pVλ241, pVλ247
**IGLV2**	13	6 (46.15)	7 (53.85)	0	Vλ199, Vλ202, Vλ205, Vλ210, Vλ213, Vλ215, pVλ200, pVλ201, pVλ203, pVλ204, pVλ209, pVλ211, pVλ214
**IGLV3**	23	12 (52.17)	10 (43.48)	1 (4.35)	Vλ158, Vλ159, Vλ166, Vλ169, Vλ170, Vλ174, Vλ177, Vλ178, Vλ183, Vλ185, Vλ190, Vλ196, Vλ176-ORF, pVλ28, pVλ157, pVλ160, pVλ163, pVλ168, pVλ175, pVλ180, pVλ181, pVλ188, pVλ193
**IGLV4**	9	1 (11.11)	8 (88.89)	0	Vλ164, pVλ25, pVλ171, pVλ179, pVλ184, pVλ186, pVλ191, pVλ194, pVλ197
**IGLV5**	60	11 (18.33)	37 (61.67)	12 (20.00)	Vλ61, Vλ67, Vλ112, Vλ115, Vλ116, Vλ125, Vλ137, Vλ142, Vλ145, Vλ152, Vλ249, Vλ32-ORF, Vλ34-ORF, Vλ41-ORF, Vλ70-ORF, Vλ86-ORF, Vλ92-ORF, Vλ102-ORF, Vλ133-ORF, Vλ232-ORF, Vλ235-ORF, Vλ248-ORF, Vλ252-ORF, pVλ18, pVλ55, pVλ58, pVλ59, pVλ60, pVλ66, pVλ69, pVλ73, pVλ76, pVλ83, pVλ87, pVλ89, pVλ91, pVλ97, pVλ98, pVλ100, pVλ105, pVλ107, pVλ108, pVλ109, pVλ119, pVλ121, pVλ122, pVλ123, pVλ128, pVλ131, pVλ134, pVλ139, pVλ149, pVλ221, pVλ226, pVλ229, pVλ238, pVλ239, pVλ242, pVλ243, pVλ245
**IGLV6**	7	2 (28.57)	3 (42.86)	2 (28.57)	Vλ57, Vλ127, Vλ24-ORF, Vλ104-ORF, pVλ42, pVλ138, pVλ254
**IGLV8**	6	4 (66.67)	1 (16.67)	1 (16.67)	Vλ53, Vλ85, Vλ155, Vλ224, Vλ96-ORF, pVλ251

Note: The pseudogenes that are too divergent or truncated to be assigned to subgroups are not counted.

### Composition of the donkey IGκ locus

Genes at the donkey IGκ locus are organized in the form of Vκ-Jκ-Cκ on chromosome 6, located on the antisense strand. The IGκ locus contains 1 Cκ gene, 5 Jκ gene segments, and 72 Vκ gene segments. Among these Vκ segments, there are 22 potentially functional Vκ genes, 5 ORFs, and 45 pVκ. Approximately 63% of the Vκ genes (45 in total) shared the same transcriptional polarity as the Jκ-Cκ genes, with both exhibiting reverse polarity (Figure 1B). 72 Vκ genes were visualized in a phylogenetic tree (Supplementary Figure S1B). According to the subgroup classification criteria, these Vκ genes were assigned to 8 IGκV subgroups (IGκV1-IGκV7 and IGκV9). pVκ that were too short or truncated and could not be assigned to corresponding subgroups were excluded from the count. The potentially functional Vκ genes belong to 7 of these subgroups (excluding IGκV5) (Table 2).

**Table 2.** Subgroup classification of Vκ genes in donkey

Subgroup	Total	Potential functional (% of total)	Pseudogene (% of total)	ORF (% of total)	Vκ gene segments
**IGκV1**	2	1 (50.00)	1 (50.00)	0	Vκ24, PVκ40
**IGκV2**	11	6 (54.55)	5 (45.45)	0	Vκ8, Vκ9, Vκ12, Vκ13, Vκ20, Vκ35, PVκ19, PVκ28, PVκ33, PVκ37, PVκ41
**IGκV3**	2	1 (50.00)	1 (50.00)	0	Vκ5, PVκ26
**IGκV4**	23	11 (47.83)	12 (52.17)	0	Vκ31, Vκ39, Vκ49, Vκ54, Vκ55, Vκ59, Vκ60, Vκ61, Vκ67, Vκ71, Vκ72, PVκ46, PVκ48, PVκ50, PVκ52, PVκ53, PVκ56, PVκ57, PVκ62, PVκ64, PVκ65, PVκ68, PVκ69
**IGκV5**	1	0	0	1 (100%)	Vκ15-ORF
**IGκV6**	1	1 (100)	0	0	Vκ21
**IGκV7**	2	1 (50.00)	1 (50.00)	0	Vκ44, PVκ30
**IGκV9**	2	1 (50.00)	1 (50.00)	0	Vκ3, PVκ23

Note: The pseudogenes that are too divergent or truncated to be assigned to subgroups are not counted.

### Analysis of donkey IGλ V(D)J recombination

**Figure 2.**
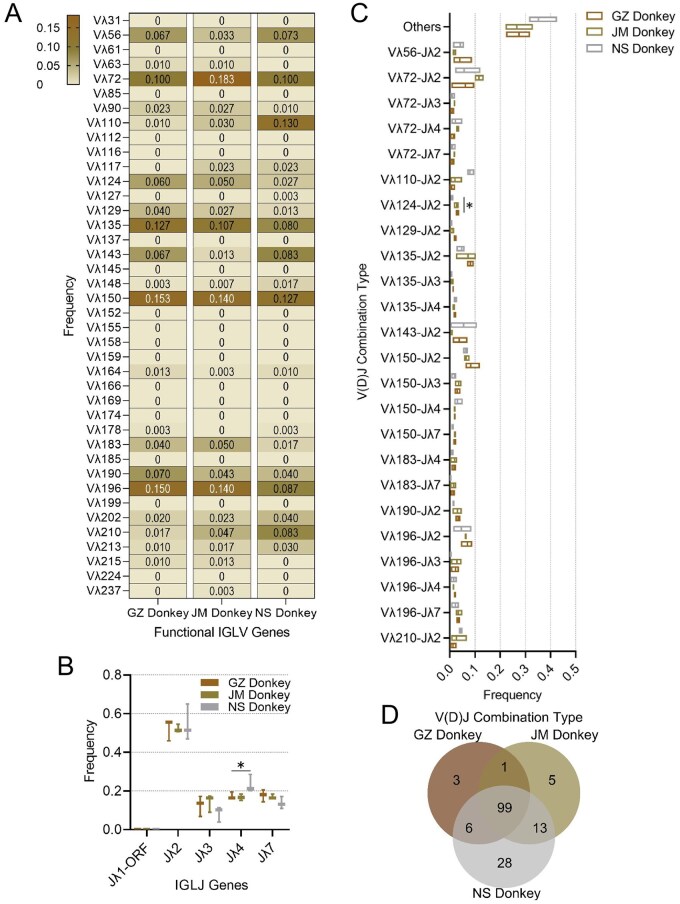
Analysis of Vλ and Jλ gene utilization and Vλ-Jλ combinations in donkeys. (A–D): Analysis of the frequency of Vλ (A) and Jλ (B) gene usage in different donkey breeds; frequency of Vλ-Jλ combinations (C) and Venn diagram analysis of the number of combination types (D) in different ­donkey breeds.

Although 51 potential functional genes were identified in the donkey germline, three donkey breeds exhibited different degrees of utilization of only 40 of these Vλ genes, which are mainly distributed across seven subgroups (IGLV1-IGLV6 and IGLV8). Analysis following alignment of donkey gene sequences with germline genes revealed that donkey populations tend to preferentially utilize genes such as Vλ72, Vλ135, Vλ150, and Vλ196 (Figure 2A), and these genes are concentrated in two subgroups (IGLV1 and IGLV3) (Table 2). Notably, the utilization rate of genes in the IGLV1 subgroup (>60%) was significantly higher than that in other subgroups (Supplementary Figure S2A). These findings indicate that despite the diversity and abundance of Vλ genes, the immune system of donkeys still exhibits certain limitations in the selection and utilization of Vλ genes.

Notably, there were significant interbreed differences in Vλ gene usage among donkeys, with interindividual variation observed. For instance, JM Donkey showed a higher utilization rate of Vλ72 than GZ Donkey and NS Donkey; NS Donkey had a higher utilization rate of Vλ110 than the other two breeds but a lower utilization rate of Vλ196. We also analyzed Vλ usage in horses. Similar to donkeys, horses preferentially utilized Vλ genes in the IGLV1 and IGLV3 subgroups, with IGLV1 being predominantly dominant (average utilization rate >63%) (Supplementary Figure S2C).

The three donkey breeds preferentially utilized the Jλ2 gene, with an average utilization rate exceeding 52%. Jλ3/4/7 were also utilized to varying degrees, among which NS Donkey ­exhibited a significantly higher usage frequency of Jλ4 than GZ Donkey (*P *< 0.05) (Figure 2B). The V(D)J recombination process generated diverse Vλ-Jλ combination types: 109, 118, and 146 combinations were identified in GZ Donkey, JM Donkey, and NS Donkey, respectively, with 100 combinations shared among the three breeds (Figure 2D). Some specific combinations (e.g., Vλ124-Jλ2) showed significant distribution differences between GZ Donkey and NS Donkey (*P *< 0.05) (Figure 2C).

Further global diversity analysis of Vλ-Jλ combinations revealed that the richness of these combinations in the NS Donkey was significantly higher than in the GZ Donkey (*P *< 0.05) (Supplementary Table S2A), whereas no statistically significant differences were observed in the Shannon diversity index or Pielou evenness index among the three donkey breeds (*P *> 0.05). These findings indicate that although the NS Donkey is capable of generating a greater diversity of Vλ-Jλ combinations in the λ-chain, the overall architecture of combination usage—particularly the distribution of high-frequency V-J pairs—remains highly consistent across the three donkey breeds. This suggests a certain degree of conservation in the fundamental patterns of V(D)J recombination among breeds, which is not substantially altered by the increase in combinatorial variety. Notably, in the two horse breeds examined, no significant differences were detected in the richness, Shannon diversity index, or Pielou evenness of Vλ-Jλ combinations (*P *> 0.05) (Table S2B).

### Analysis of donkey IGκ V(D)J recombination

**Figure 3.**
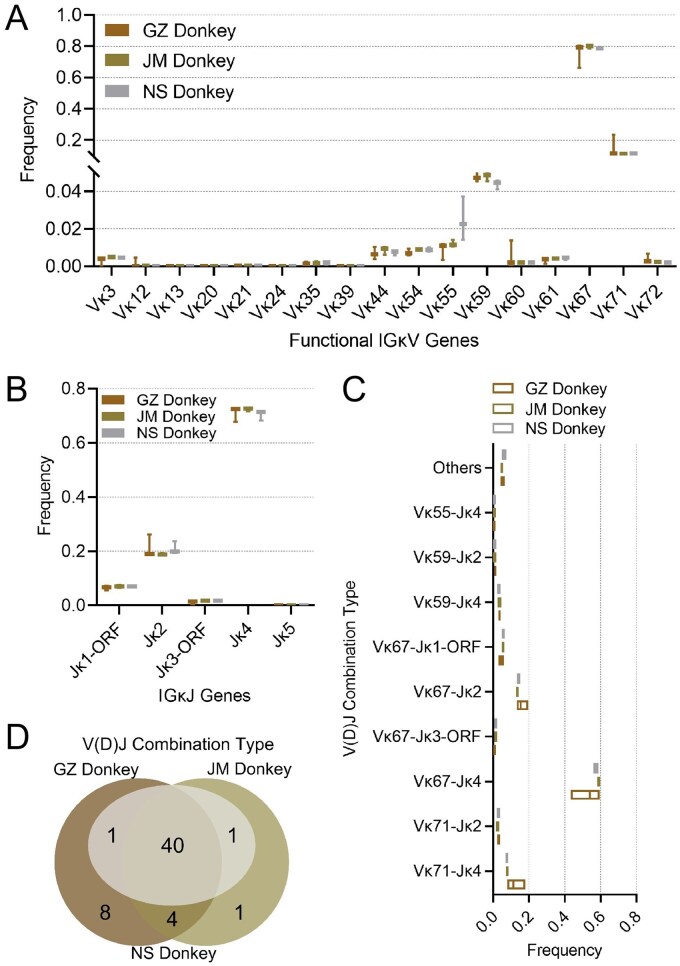
Analysis of Vκ and Jκ gene utilization and Vκ-Jκ combinations in donkeys. (A–D): Analysis of the frequency of Vκ (A) and Jκ (B) gene usage in different donkey breeds; frequency of Vκ-Jκ combinations (C) and Venn diagram analysis of the number of combination types (D) in different donkey breeds.

Matching the Vκ sequences from three donkey breeds with the 22 potentially functional germline Vκ genes revealed the utilization of 17 genes across IGκV1/2/4/6/7/9 subgroups. The IGκV4 subgroup dominated in all breeds, with usage frequencies exceeding 99% (Supplementary Figure S2B). Overall, the utilization of the Vκ gene in different donkey breeds showed certain common patterns. The usage rates of Vκ67 and Vκ71 were significantly higher than those of other genes, approximately 77.9% and 12.5%, respectively, both of which belong to the IGκV4 subgroup. Moreover, no significant difference in the utilization rate of advantageous genes was observed among the three varieties (*P *> 0.05) (Figure 3A).

All three donkey breeds exhibited high usage rates for Jκ4 (approximately 70%) and Jκ2 (approximately 20%) (Figure 3B). Analysis of Vκ-Jκ combination types revealed 53, 46, and 42 combinations in GZ Donkey, JM Donkey, and NS Donkey, respectively, with 44 shared combinations among the three breeds (Figure 3D). Notably, all three breeds showed high utilization of three combination types: Vκ67-Jκ4, Vκ67-Jκ2, and Vκ71-Jκ4, which accounted for approximately 80% of the total combinations with no significant interbreed differences (Figure 3C). Global diversity analysis of V(D)J recombination combinations showed that the three donkey breeds exhibited no significant differences in recombination richness, Shannon diversity index, or Pielou evenness for the κ chain (*P *> 0.05) (Supplementary Table S2A). This result indicates that different donkey breeds maintain a high degree of consistency in dominant Vκ-Jκ combination patterns and overall recombination diversity, further suggesting that the κ chain may be subject to stronger evolutionary or functional constraints during V(D)J recombination, thereby exhibiting marked conservation across breeds. Similarly, in the κ chain of the two horse breeds studied, these diversity metrics also showed no statistically significant differences (*P *> 0.05) ­(Supplementary Table S2B). This finding implies that the stability of κ chain recombination diversity at the intraspecific level is not unique to donkeys but may represent a more general conserved feature within the genus Equus. Moreover, compared to the κ chain, the λ chain exhibits significantly greater recombination diversity in both donkeys and horses.

### Diversity of donkey IGλ junctions and CDR3λ analysis

During Vλ-Jλ joining, the average lengths of the 3′V-Region and 5′J-Region were approximately 14 to 15 and 7 to 8 nucleotides, respectively, with significant differences observed between donkeys and horses as well as among breeds (*P *< 0.01). The contributions of N-nucleotide (13–16 nt) and P-nucleotide (approximately 0.1 nt) insertions to IGλ variable region diversity also exhibited heterogeneity between species and breeds, which was mainly attributed to variations in the insertion length of N-nucleotides (Supplementary Table S3A).

**Figure 4.**
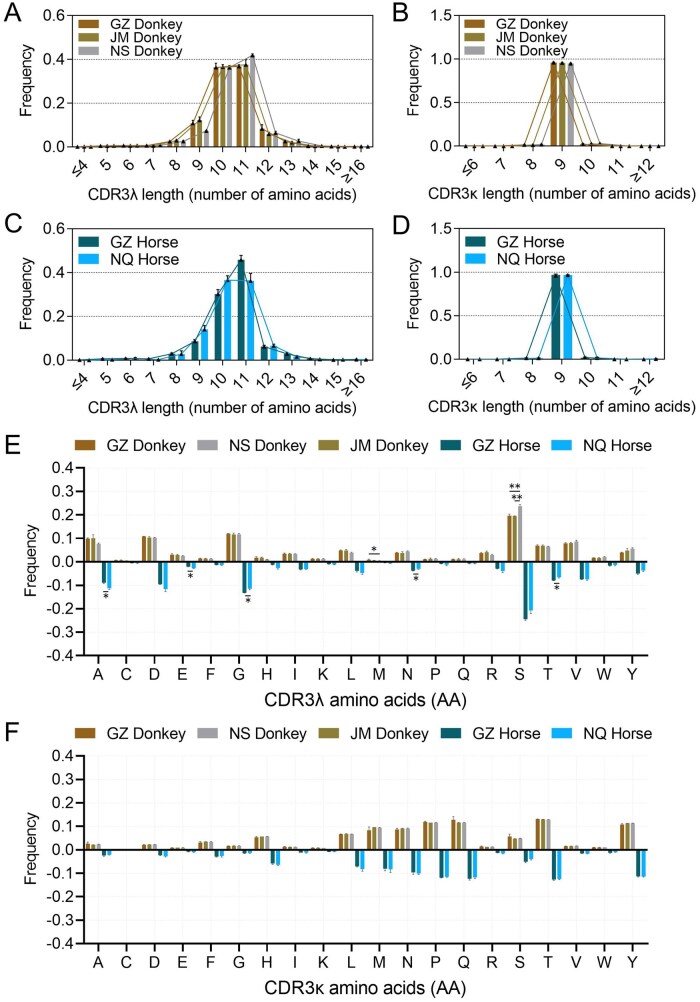
Analysis of CDR3λ and CDR3κ in donkeys and horses. (A–F): AA lengths of GZ, JM and NS Donkey CDR3λ (A) and CDR3κ (B); AA lengths of GZ and NQ Horse CDR3λ (C) and CDR3κ (D); Donkey and Horse CDR3λ (E) and CDR3κ (F) AA composition.

The CDR3λ length of donkeys ranged from 3 to 32 amino acids (aa), with a primary concentration in the 10–11 aa range. This length accounted for over 70% of the total, with an average utilization rate exceeding 73% across the three donkey breeds. Interbreed comparison showed no significant differences in CDR3λ length distribution (Figure 4A). Horses exhibited a similar CDR3λ length range to donkeys but displayed interbreed differences in specific length usage. As shown in Figure 4C, GZ Horse had a significantly lower usage frequency of 9-aa CDR3λ (approximately 8.6%) than NQ Horse (approximately 14.0%) (*P *< 0.05). Further analysis revealed that GZ Horse showed a higher usage rate of 11-aa CDR3λ and a lower rate of 10-aa CDR3λ compared to NQ Horse; although this difference did not reach statistical significance (*P *> 0.05), a certain complementary distribution trend was observed. Additionally, GZ Horse exhibited significantly higher usage rates of 7-aa and 14-aa CDR3λ than NQ Horse, but due to their extremely low overall proportions (less than 1% each), these differences had limited biological significance and were not further explored in this study. Notably, no significant interspecies differences were found in the overall CDR3λ length distribution pattern between horses and donkeys (*P *> 0.05), indicating that CDR3λ length characteristics are conserved between the two species.

Analysis of the amino acids composition of CDR3λ in donkeys and horses revealed a pronounced preference for serine (S), glycine (G), aspartic acid (D), and alanine (A) in both species (Figure 4E). However, breed-specific differences were also observed: among donkeys, the usage frequency of serine was significantly higher in NS Donkey compared to GZ and JM Donkey (*P *< 0.01), while in horses, significant variations were detected in the utilization frequencies of alanine and glycine, among other amino acids (*P *< 0.05). Notably, cysteine (C) occurred at a very low frequency across all samples, ranging only from 0.4% to 0.7%, with no statistically significant differences observed between species or breeds (*P *> 0.05). It is known that cysteine residues in the CDR3 region can contribute to structural complexity and stability through the formation of intra-loop disulfide bonds. However, data from this study indicate that the vast majority of CDR3λ sequences in both donkeys and horses lack cysteine (Supplementary Table S4).

### Diversity of donkey IGκ junctions and CDR3κ analysis

Unlike Vλ-Jλ junctions, Vκ-Jκ joining were highly consistent across donkeys, horses, and breeds (*P *> 0.05), with average 3'V-Region and 5'J-Region lengths of 22-23 and 8-9 nucleotides, respectively. The contribution of N-nucleotides was around 1-2 nucleotides, significantly lower than in IGλ ­(Supplementary Table S3B).

CDR3κ lengths in donkeys ranged from 4 to 45 aa, with a concentration at 9 aa (>95%), showing a unimodal distribution (Figure 4B). CDR3κ lengths in GZ Horse and NQ Horse also displayed a peak at 9 aa (Figure 4D), without significant inter-breed differences, indicating significant conservation of CDR3κ amino acids lengths across species.

The CDR3κ regions of both donkey and horse antibodies exhibited a high utilization frequency for threonine (T), proline (P), tyrosine (Y), glutamine (Q), methionine (M), asparagine (N), and leucine (L); and an extremely low utilization rate for cysteine, even lower than that of the λ chain, at merely 0.15% to 0.22%. Furthermore, no significant differences in amino acids utilization frequencies were observed between horse and donkey breeds. (Figure 4F). Over 97% of CDR3κ sequences lacked Cys, with rare single-Cys occurrences and nearly no double-Cys sequences (Supplementary Table S4B). In addition, correlation analysis revealed that the absolute value of the correlation coefficient between the number of cysteines in the CDR3 region and CDR3 length was less than 0.15. This indicates an extremely weak correlation between Cys count and CDR3 length in both λ and κ chains (*P *< 0.01), suggesting that the cysteine content in CDR3 is not affected by length ­variations (Supplementary Table S5).

### Analysis of IGλ SHM

**Figure 5.**
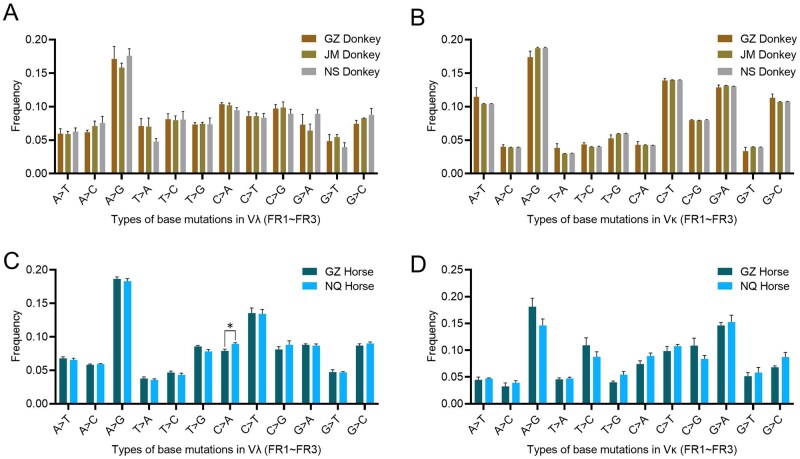
Statistics of base mutation types in donkey and horse Vλ and Vκ genes (FR1-FR3). (A–D): GZ, JM and NS Donkey Vλ (A) and Vκ (B) base mutation types; GZ and NQ Horse Vλ (C) and Vκ (D) base mutation types.

**Figure 6.**
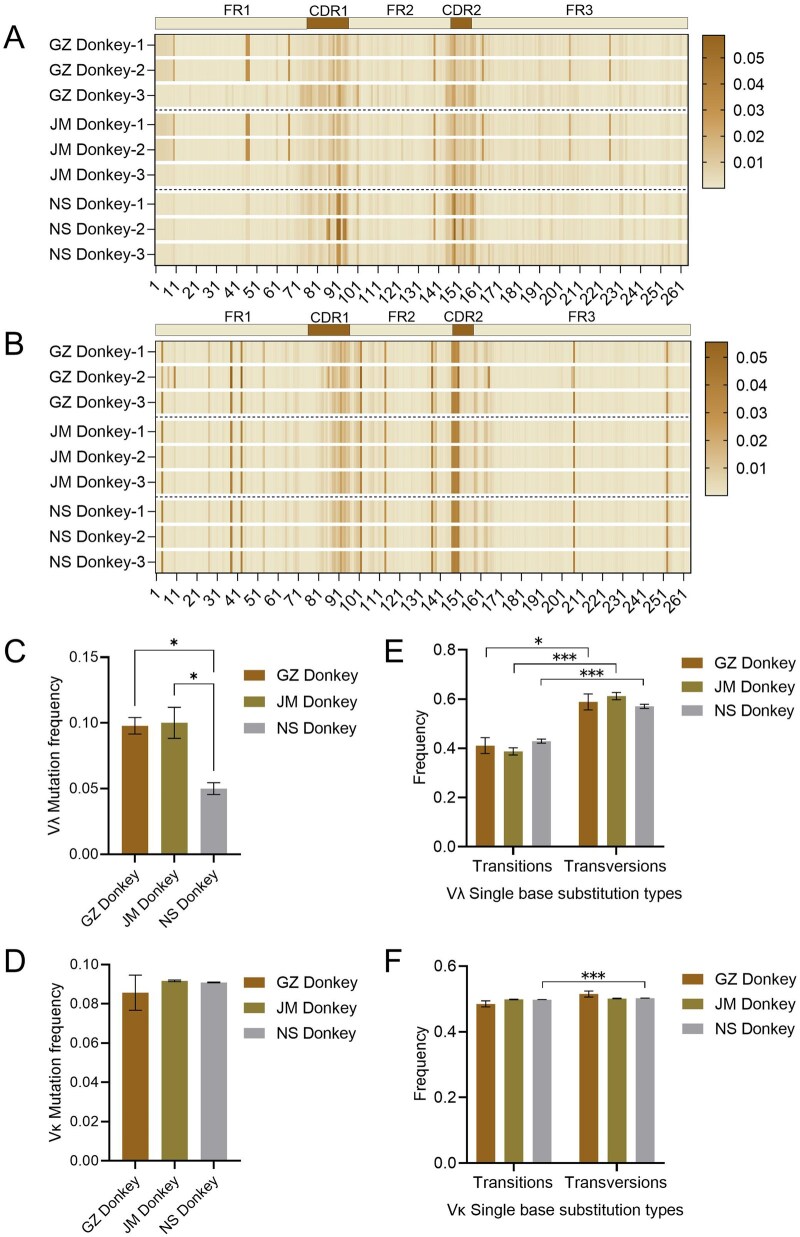
Somatic hypermutation (SHM) analysis of donkey V gene (FR1-FR3). (A–F): GZ, JM and NS Donkey Vλ (A) and Vκ (B) base mutation position; Vλ (C) and Vκ (D) total mutation rate; Vλ (E) and Vκ (F) transitions and transversions occurrence frequency.

Due to the high frequency of Vλ150 usage in donkeys, it was selected as a template for SHM analysis. Sequencing results revealed that the mutation rate for “A > G” in the Vλ gene aligned with Vλ150 was significantly higher (>15%) compared to other mutation types (*P *< 0.01). Mutation types showed no significant variation among the three donkey breeds (*P *> 0.05) (Figure 5A). Additionally, transversions occurred at a significantly higher frequency than transitions in the Vλ variable region (*P *< 0.05) (Figure 6E). The total mutation rate in the IGλ variable region ranged from 4.3% to 11.6%, with GZ Donkey (9.8%) and JM Donkey (10.0%) exhibiting significantly higher rates than NS Donkey (5.0%) (*P *< 0.05) (Figure 6C). SHM preferentially occurred in the CDR1 and CDR2 regions, while mutation patterns in the FR regions displayed individual and breed-specific differences (Figure 6A).

Similar to donkeys, horses also displayed high-frequency mutations of “A > G” and “C > T.” The mutation rate of “A > G” in Vλ (represented by KF985051) (>18%) was significantly higher than other mutation types, and the frequency of “C > A” mutations in NQ Horse was significantly higher than that in GZ Horse (*P *< 0.05) (Figure 5C). However, no significant difference in the total SHM rate (5.6–8.6%) of the Vλ variable region was observed between the two horse breeds (Figure S3C). Consistent with donkeys, horses also showed a significantly higher frequency of transversions than transitions in Vλ genes (*P *< 0.01) (Supplementary Figure S3E). SHM mutations were preferentially localized in CDRs and the junctional regions between CDRs and FRs (Supplementary Figure S3A).

To further investigate whether the frequency of transitions and transversions in the variable regions is related to the selection of gene subgroups, the analysis was extended to the IGLV3 subgroup, where usage rates in both donkeys and horses were second only to IGLV1. The Vλ196 gene from donkeys and the Vλ gene (KF985100) from horses, both belonging to the IGLV3 subgroup, were used as templates. The results showed that in both donkeys and horses, the frequency of transitions was significantly higher than that of transversions (*P *< 0.05), with no significant differences between breeds (Supplementary Figure S4A and B). This suggests that genes from different subgroups may exhibit specific differences in SHM patterns.

### Analysis of IGκ SHM

Given the significant preference for IGκV4 subgroup genes in the κ chain variable regions of both donkeys and horses, Vκ67 (donkey) and Vκ4-1 (horse, referenced from the IMGT database) were selected as representative sequences for SHM analysis, following the same methodology as for Vλ chains. Results showed that the “A > G” base mutation was the most frequent among all analyzed breeds in both Vλ (Figure 5A) and Vκ (Figure 5B) chains, indicating a conserved base mutation hotspot in the IGL variable regions of horses and donkeys. Regarding the overall mutation rate (FR1-FR3), the donkey Vκ chain exhibited a range of 6.8% to 9.8% (Figure 6D), while the horse Vκ chain exhibited a rate of 4.2% to 5.9% (Fig. S3D). Furthermore, the overall mutation rate showed no statistically significant differences between breeds within each species (*P *> 0.05), suggesting that somatic high-frequency mutation rates are primarily determined by species factors rather than breed influences.

Regarding mutation types, NS Donkey exhibited a significantly higher frequency of transversions than transitions in the κ chain (*P *< 0.01) (Figure 6F), showing a unique mutation preference. In contrast, the transition-to-transversion ratio was approximately 1:1 in other donkey breeds with no significant differences. No significant differences in transition and transversion frequencies were observed between the two horse breeds (Supplementary Figure S3F). These results indicate that, except for NS Donkey, SHM in the κ chain of donkeys and horses tends to maintain a basic balance between transitions and transversions, suggesting similar DNA repair and mutation mechanisms across most breeds.

Furthermore, SHM mutations in donkeys were more likely to occur in CDR1, CDR2, the anterior and middle parts of FR1, the anterior-middle and middle-posterior parts of FR2, the middle and posterior parts of FR3, and the junctional regions between FRs and CDRs, with similarities among breeds (Figure 6B). In horses, SHM mutations were preferentially localized in CDR1, CDR2, the junctional regions between FRs and CDRs, and the anterior part of FR3 ­(Supplementary Figure S3B).

## Discussion

During B-cell differentiation, IGH rearrangement precedes IGL rearrangement (Alt et al., 1981). Mammalian species exhibit two light-chain types, IGλ and IGκ. Earlier studies suggested that IGκ rearrangement occurs before IGλ (Hieter et al., 1981), but recent research indicates a species-specific sequence of IGκ → IGλ → IGH → IGκ → IGλ, suggesting independent evolutionary paths across species (Sinkora et al., 2020; Sinkora et al., 2022). Some researchers suggest that the predominant use of a particular light chain may be attributed to the number of germline V gene segments present in a species. The primary diversification of the mouse IGκV repertoire has been linked to the insufficient contribution of its λ chain. In other words, the mouse may have lost significant portions of the IGλV loci, which may have driven structural diversification within the IGκV loci ­(Almagro et al., 1998). Light chain usage in mice is also influenced by factors such as higher recombination frequency at the IGκ locus, antigen selection, and increased autoimmunity in IGκ-deficient mice, which suggests negative selection on IGλ (Ramsden and Wu, 1991; Knott et al., 1998; Tallmadge et al., 2014). Unlike mice, horses predominantly use the λ chain (Arun et al., 1996; Wagner, 2006). Similarly, our study reveals a higher number of Vλ genes in donkeys compared to Vκ genes, with Vλ exhibiting broader utilization, suggesting that donkeys, like horses, may preferentially employ the λ chain.

Notably, in both donkeys and horses, highly utilized Vλ genes predominantly belong to the IGLV1 and, to a lesser extent, the IGLV3 subgroups. Evidence suggests that IG genes in various species preferentially utilize specific subgroups. For instance, porcine Vλ genes are predominantly derived from the IGLV3 and IGLV8 subgroups (Schwartz et al., 2012; Wertz et al., 2013), while over 95% of Vκ-Jκ rearrangements use genes from the IGκV2 family (Butler et al., 2005; Butler et al., 2006). Following PEDV vaccination, porcine B cells in peripheral blood and mesenteric lymph nodes primarily utilize IGLV3 and IGκV1 (rather than IGκV2) subgroups (Li et al., 2020). Similarly, bovines prefer IGLV1 and IGκV2 (Wu, 2022; Li et al., 2023), while sheep favor IGLV1 and IGκV1 subgroups (Park et al., 2023). In minks, λ chains mainly employ IGLV3, while κ chains predominantly use IGκV1 (Yi et al., 2024).

In this study, donkeys exhibited a high-frequency usage of Vκ67 and Vκ71 genes, which share a high sequence similarity (95.83%) and are both classified within the IGκV4 subgroup (Table 2). This suggests significant evolutionary relatedness and potential similarities in antigen recognition mechanisms, enabling these genes to play an efficient role in the donkey immune system. Furthermore, genomic sequencing of three donkey breeds revealed that 99% of the functionally utilized Vκ genes belong to the IGκV4 subgroup (Supplementary Figure S2B), underscoring the dominant role of the IGκV4 subgroup in donkey immune responses.

Furthermore, in the κ-chain gene repertoire of donkeys, potential functional genes of the IGκV4 subgroup account for 50% of the total number of potential functional germline Vκ genes. In contrast, the combined potential functional genes of the remaining seven Vκ subgroups constitute only the other 50% (Table 1). This distribution pattern indicates a significant enrichment of potential functional genes in the IGκV4 subgroup at the genomic level. This phenomenon is conserved among horses. As reported by Sun et al. (2010), the most frequently expressed subgroup in the horse κ-chain also contains a high proportion (40–50%) of potential functional genes.

Further analysis revealed that this rule also holds true for the λ-chain. In donkeys, the dominant subgroups (IGLV1 and IGLV3) in the antibody repertoire collectively harbor 52.9% of all potential functional germline Vλ genes, while the combined potential functional genes of the other six λ-chain subgroups account for only 47.1% (Table 1). Correspondingly, in the horse λ-chain, the dominant subgroups in the antibody repertoire contain 33.3% of the total potential functional germline Vλ genes (Sun et al., 2010).

Collectively, these data indicate that the dominantly used subgroups in the antibody repertoire of horse and donkey light chain genes are characterized by an enrichment of potentially functional genes at the genomic level. However, it is important to emphasize that the high-frequency usage of a subgroup in the antibody repertoire does not directly imply a higher proportion of “functional genes” within that subgroup. In fact, the present study found that in the light chain genes of both donkeys and horses, subgroups with a larger absolute number of potential functional genes often exhibit a concurrent increase in the number of pseudogenes (Table 1). This phenomenon of “synchronous expansion” of functional genes and pseudogenes within dominant subgroups is prevalent in both κ and λ light chain genes of donkeys and horses, suggesting that this may reflect a conserved genomic structural feature during the evolution of IG genes in equids.

The addition of N-nucleotides to coding and signal joints during V(D)J recombination is predominantly mediated by TdT (Gilfillan et al., 1993; Komori et al., 1993). TdT is an enzyme specifically expressed in developing lymphocytes, responsible for adding random N-nucleotides to the free 3′ ends of DNA breaks (Sandor et al., 2004). This mechanism increases the diversity of T cell receptor (TCR) and IG molecules, which is crucial for the adaptive immune system’s ability to recognize a wide range of antigens (Zhang et al., 1995; Komori et al., 1996). Furthermore, studies have confirmed that the contribution of P-nucleotides to the diversity of IG sequences is less than 1%, with most P-nucleotides likely being inserted at the junctions during the addition of N-nucleotides (Jackson et al., 2004). The minimal contribution of P-nucleotides to the diversity of IG expression libraries aligns with the findings of the present study.

In IG and TCR, lymphocyte V, D, and J segment rearrangement during development generates substantial diversity in the CDR3 region. The length of CDR3λ and CDR3κ regions exhibits certain patterns across different species. In sheep, CDR3λ and CDR3κ lengths vary from 1 to 27 and 1 to 24, respectively, with distributions concentrated at 10-11 aa and 9 aa (Park et al., 2023). Similarly, dogs predominantly exhibit CDR3λ and CDR3κ lengths of 11 aa and 9 aa, respectively, with consistency across small, large, and laboratory beagle breeds (Steiniger et al., 2014). In yaks, CDR3λ and CDR3κ length preferences align with those of sheep and dogs (Wu et al., 2022). In avian species, such as Peking ducks and chickens, also show CDR3λ lengths concentrated at 9 aa (Guan et al., 2016; Qiu et al., 2024). In this study, donkeys and horses share similar CDR3λ and CDR3κ length characteristics, reflecting interspecies conservation.

Notably, CDR3 lengths vary among chains. Humans and mice show broader CDR3 length distributions for IGH and TCRδ, significantly longer than κ and λ chains, as well as α, β, and γ chains, respectively (Rock et al., 1994). Donkeys and horses exhibited mean CDR3H lengths of 14.91 aa and 15.66 aa, respectively (Qiu et al., 2025), also longer than their CDR3λ and CDR3κ lengths. The ultra-long CDR3H regions in cattle form cysteine-rich “stalk-and-knob” structures, stabilized by disulfide bonds that create spatial diversity (Wang et al., 2013; Arras et al., 2023), compensating for V(D)J recombination limitations. In our study, significant differences in the amino acid composition of CDR3λ and CDR3κ were found in donkeys and horses, but both had a lower utilization of Cys. This suggests that the light chains of donkey and horse may struggle to maintain their levels of antibody library diversity through a mechanism of structural stabilization of disulfide bonds similar to that of the bovine ultra-long CDR3H region.

It is found that in the IGLV1 subgroup, the frequency of transversions in donkey and horse Vλ genes is markedly higher than that of transitions. In molecular evolution, transition mutations are generally favored over transversions because transitions tend to have more conservative effects on proteins (Keller et al., 2007; Stoltzfus and Norris, 2016). However, when performing a transition-transversion analysis using a representative gene from the IGLV3 subgroup as a template, both donkey and horse exhibited a higher frequency of transitions, with no interbreed differences (Figure S4). This suggests that the higher frequency of transversions in the IGLV1 subgroup may be linked to its frequent usage, highlighting gene usage frequency as a potential determinant of mutation type distribution and offering new insights into the evolution of immune genomes.

From the perspective of individual and population immunology, each individual has its own antigen recognition system. Different individuals exhibit shared antibody responses with similar amino acid characteristics against the same antigen. These common antibodies are often encoded by identical or similar V genes, and specific amino acid residues enable them to bind to the same antigen, suggesting convergent immune responses across individuals when facing the same pathogen (Thomson et al., 2008; Watson et al., 2017). However, since these convergent antibodies are encoded by the individual genome, there may still be some degree of individual bias in immune responses, which could be influenced by genetic and environmental factors (Watson et al., 2013). This study revealed that although differences in IGL gene expression patterns (such as specific V-J gene combinations) exist among different donkey breeds, these variations are predominantly concentrated on the λ chain rather than the κ chain. Moreover, even within the λ chain, the vast majority of differences occur in non-dominant, infrequently used genes. Thus, we conclude that the core components of the functional antibody repertoire—particularly the highly expressed genes and subgroups—remain highly conserved across the donkey breeds examined in this study.

Equine-derived IG genes have been widely used in the development of recombinant antibodies (Liu et al., 2022; Ruschig et al., 2024). For example, traditional antitoxin and antivenom therapies rely on polyclonal antisera obtained from large animals such as horses. While lifesaving, these treatments present challenges related to consistency, safety, and large-scale production. Recombinant antibodies, as alternatives, offer more consistent, safer, and more effective treatments. Rosenfeld et al. (2022) laid the foundation for equine-derived recombinant antibody development by amplifying equine IGV gene segments and successfully identifying neutralizing antibodies that target specific epitopes of botulinum toxin. Furthermore, previous studies have successfully utilized the preferential usage of VH and Vλ genes in horses, employing rapid and high-throughput methods to isolate specific monoclonal antibodies (Wibmer and Mashilo, 2022). Donkeys, as smaller animals with stronger immune capabilities, offer lower rearing costs compared to horses. Additionally, the similarity in IG gene expression patterns between donkeys and horses makes donkeys an ideal candidate for antibody production.

## Conclusion

This study elucidates the structural features of donkey IGLV and IGκV genes and their preferential usage among subgroups. Through an in-depth analysis of the variation and expression patterns of these genes, this research provides a theoretical basis for the development of specific antibodies in donkeys, as well as offering new insights into breeding animals for disease resistance. These findings hold significant potential applications, particularly for improving livestock productivity and enhancing disease resistance.

## Supplementary Material

skag001_Supplementary_Data

## Data Availability

Sequence data that support the findings of this study have been deposited in the NCBI (https://submit.ncbi.nlm.nih.gov/), with the primary accession code PRJNA1245107 and PRJNA1245155.

## Abbreviations:

5'RACE: rapid amplification of cDNA the 5' ends
aa: amino acids
AID: activation-induced cytidine deaminase
C: constant region
CDR: complementarity-determining region
CSR: class switch recombination
Cys: cysteine
D: diversity gene segment
DSB: double strand breaks
FR: framework region
IG: immunoglobulin
IGH: immunoglobulin heavy chain
IGL: immunoglobulin light chain
IGκ: immunoglobulin κ chain
IGλ: immunoglobulin λ chain
Jκ: κ chain joining gene segment
Jλ: λ chain joining gene segment
ORF: open reading frame
RAG: recombination activating genes
RSS: recombination signal sequences
SHM: somatic hypermutation
TCR: T cell receptor
TdT: terminal deoxynucleotidyl transferase
V: variable region
Vκ: κ chain variable gene segment
Vλ: λ chain variable gene segment
