# Supplementary material for: Comparative analysis of the organization and complexity of immunoglobulin light chain loci in equids
Source: J Anim Sci. 2026 Jan 9;104:skag001. doi: 10.1093/jas/skag001 (PMC12906693; doi:10.1093/jas/skag001)
Supplement: skag001_Supplementary_Data [file skag001_supplementary_data.zip › 22-Jan-2026_070757_Supplementary_material_A.pdf]

Supplementary material A-Vλ and Vκ genes sequence

Vλ gene sequence

>pVL1

CAGGCAGGGCTGACTCAGCCGCCCTCAGTGACCAAGTCCCCAGGACAGACGGCCACACTCACCTGCA
CTGGAAAGAGCAAAAATGTCGGCCATGAGGGGGGCAGCGTGGCTGCAGCAGCAACCCCAAGGCCGTG
TCCCCACACTCCTGACCAGCAGGAATAACATCTGGGCCTCTGGGGTCTCCGAGAGATTCTCTGGCTCC
AGGTGAGGCAGTGTGGCACCCTGAGCATCTCTGGGCTCCAGCCTGGAACGAGGCTGATTATTACTGC

>VL2-ORF

CAGTCTGTGCTGACTCAGCAGGCCTCAGTGTCTGGGAATCTGGGCCAGAGGGTCACCATCTCCTGCAC
TGGAAGCAGCTCCGACATCAGTGATTATGATGTGCACTGGTACCAACAGCTCCCGGGAATGGCCCCCA
AACTCATCATCTATGATAACAGCAAACAGCCATCTGGGGTCCCAGAAGGATTCTCTGGCTCCAAGTCT
GGCAACTCGGCCACCCTGACCATCACTGGGCTCAAGTCTGAGGACGACGCTGATTATTACTGT

>pVL3

CAGCCTGTGTGACTCAGTTACTCTCTGCCTCTGCCTCCCTGGGAGCTTTGGCCATGCTCAGCTGCACCC
TGAGCAGTGAGCAGAGCACCTTCTACATCGGATGGTGTCAACAGCACCCTAGGAAGACCCCTCAGGA
TGTGACGTGCCTGAAGAGTGAAGGAAGCCACAGCAAGGGGGATGGAATTCCTGATCATTTCTGGGGC
TCCAGCTCTGGGGCTGACCGCTACTCAACCATCCCCACCAGCCAGTCTGTGGAGCATCTCACAGCATT
GCTGT

>pVL4

GCCAGTGCTGACTCAGCCACCTTCCCTCTGCATCACCTGGAGCAAATGCCAGACTCACCTGCACTGCC
AGCAGTGACATCAGTGTTGGTGGATACAGGATATTCTGGACCAGCCGAAGCCAGGGAGCCCCCAGGT
ATCTCTTGATCTACCACACAGACTCAAATGAGCATCAGGGCCCAGGATCCACAGCCACTTCTCTGGAT
CCAAAGATGACACAACTAACTCTGGTGTTCTGCTCGCCTCCGGGCTGCGACCTGAGGACAAGGCTCAC
TATTACTGT

>pVL5

CAGCCTGTGTGACTCAGTTACTCTCTGCGTCTGCCTCCCTGGGAGCTTTGGCCATGCTCAGCTGCACCC
TGAGCAGTGAGCAGAGCACCTTCTACATCGCATGGTGTCAACAGCACCCTAGGAAGGCCCCTCAGGAT
GTGACGTGCCTGAAGAGTGAAGGAAGCCACAGCAAGGGGGACGGAATTCCTGATCATTTCTGGGGCT
CCAGCAGCTCTGGGGCTGACCGCTACTCAACCATCCCCACCAGCCAGTCTGTGGAGCATCTCACAGCA
TTGCTGT

>pVL6

CAGTCTGTGCTGACTCAGCCGGCCTCAGTGTCTGGGAACCTGGGCCAGAGGGTCACCATCTCCTGCAC
TGGGAGCAGCTCCGACATCGGTGATTATGATGTGCACCGGTACCAACAGCTCCCGGGAATGGACCCCA
AACTCGTCATCTATGATAACAGCTAACGGCCATCTGGGGTCCCAGAATGGTTCTCTGGCTCCAAGTCT
GGCAACTTGGCCACCCTGACCATCACTGGACTCAAGTCTGAGGACGATGCTGATTATTACTGT

>pVL7

CAGCCTGTGTGACTCAGTTACTGTCTGCATCTGCCTCCCTGGGAGCTTCGGCCATGCTCAGCTGCACCC
TGAGCAGTGAGCAGAGCACCTTCTACATCGGATGGTGTCAACAGCACCCTAGGAAGGCCCCTCAGGA
TGTGACGTGCCTGAAGAGTGAAGGAAGCCACAGCAAGGGGGATGGAATTCCTGATCATTTCTGGGGC
TCCAGCTCTGGGACTGACCGCTACTCAACCATCCCCACCAGCCAATCTGTGGAGCATCTCACAGCATT
GCTGT

>pVL8

GCCAGTGCTGACTCAGCCACCTTGCCTCTGCATCACCTGGAGCATATGCCAGACTCACCTGCACTGCC
AGCAGTGACATCAGTGTTGGTGGATACAGGATATTCTGGACCAGCCAAAGCCAGGGAGCCCCCAGGT
ATCTCTTGCTCTGCCACACAGACTCAAATGAGCATCAGGGCCCAGGATCCCCAGCCACTTCTCTGGAT
CCAAAGATGACACAACTAACTCTGGTGTTCTGCTCGTCCCCGGGCTGCGACCTGAGGACGAGGCTCAC
TATTACTGT
>pVL9
CAGATGGGACTGACTCAGCCACCCTCAGGGCCCAAGTCCCTGGGATAGACAGGCACACTCACCTGCAT
GGTAAACAGCAACAGTGTTGGCCAACAGAGAGCAGCATGGCTGCAGCAGCTCCTGGGCCATGTCCCC
AAACTCCTGATCCACAGGAGTAACAGCTTGCTGTCAGGGGTCTCTGAGAGATTCTCTGGCTCCAGGTC
AGACAGTGTGACCTCCTAGATGTCACTGCGATGGCCCAATCCTTCATTCCTGAAAGGTACGGAC
>pVL10
CAGTCTGGGCTGACACAGGAAGCCTCAGTGTCTGGGTCTGTGGGACAGAAAGTCACCCTCTCCTGTGC
TGGAAATAGCAACAACGTTGGAATATTTGATGTGGGCTGGTACCAAATTTCTCACGGTGCCCCCAAAA
CTGTGACGCTCAGAAGTTCTCGTCCCTCAGGGATCCCAGCTCGGTTCTCTCTGGCTCCAAGTCCGGCAA
CACGGCCTATCTGACCATCTCTGGGCTCCAGCCTGAGGATGAGGCTGATTATTACTGT
>pVL11
TTGCTGACTCAGTGTCCTCCCTCTCTGCACCTCTGGGAACATCAGCCAGACTCATCTGCATCCTGAGCA
GTGTTCTCAGTGGTGGGGGCTTCTGGATGAGCTCATAGCAGCAGAAGGAGGGAGCCCTCCCCAGTATC
TCCTGAGTTTCAAGTCAGACTCAGGTAAGAATCAGGGTTCCAGGCTCCCCAGCCACCTCTCTGGATCC
AAAGTTGCCTCCACTGACTCAGATATTTTATTTCTCTGGGCTGCAGCCTGAAGAGGGTGACATTACTGT
>pVL12
CAGGCAGGGCTGACTCAGCCGCCCTCAGTGACCAAGTCCCCAGGACAGACGGCCACACTCACCTGCA
CTGGAAAGAGCAAAAATGTCGGCCATGAGGGGGCAGCGTGGCTGCAGCAGCAACCCCAAGGCCGTGT
CCCCACACTCCTGACCAGCAGGAATAACATCTGGGCCTCTGGGGTCTCCGAGAGATTCTCTGGCTCCA
GGTGAGGCAGTGTGGCACCCTGAGCATCTCTGGGCTCCAGCCTGGAACGAGGCTGATTATTACTGCTC
>pVL13
CAGTCTGTGCTGACTCAGCAGGCCTCAGTGTCTGGGAATCTGGGCCAGAGGGTCACCATCTCCTGCAC
TGGAAGCAGCTCCGACATCAGTGATTATGATGTGTGCACTGGTACCAACAGCTCCCGGGAATGGCCCC
CAAACTCATCATCTATGATAACAGCAAACAGCCATCTGGGGTCCCAGAAGGATTCTCTGGCTCCAAGT
CTGGCAACTCGGCCACCCTGACCATCACTGGGCTCAAGTCTGAGGACGACGCTGATTATTACTGT
>pVL14
CAGCCTGTGTGACTCAGTTACTCTCTGCGTCTGCCTCCCTGGGAGCTTTGGCCATGCTCAGCTGCACCC
TGAGCAGTGAGCAGAGCACCTTCTACATGGGATGGAATCAACAGCACCCTAGGAAGACCCCTCAGGA
TGTGACGTGCCTGAAGAGTGAAGGAAGCCACAGCAAGGGGGATGGAATTCCTGATCATTTCTGGGGC
TCCAGCTCTGGGGCTGACCGCTACTCAACCATCCCCACCAGCCATTCTGTGGAGCATCTCACAGCATT
GCTGT
>pVL15
GCCAGTGCTGACTCAGCCACCTTGCCTCTGCATCACCTGGAGCATATGCCAGACTCACCTGCACTGCC
AGCAGTGACATCAGTGTTGGTGGATACAGGATATTCTGGACCAGCCAAAGCCAGGGAGCCCCCAGGT
ATCTCTTCTTGCTCTGCCACACACAGACTCAAATGAGCATCAGGGCCCAGGATCCCCAGCCACTTCTCT
GGATCCAAAGATGACACAACTAACTCTGGTGTTCTGCTCGTCCCCGGGCTGCGACCTGAGGACGAGGC
TCACTATTACTGTA
>pVL16
TCTTTGCTGACCCAGCCATCCTCGGTGTCAGGGACCCTGGACCAGGAGGTCACCATCTCCTGCTCCGG

GAACAGCAACATTGGATCCCATAGTGTGTGTTGGTTCCAGCTGTCAGTGGTGCTGCCCCCCAAAACTC
TGACTCTTGGAAGTACCAGGCCCTGAGAGAGATTCCAGATGATTCTCTGTCTCCAAGTCAGGCAACGT
GGTCTCTTTGACCATCTGTGGGCTCCAGCCTGAGGACGAGGTGATTGTTACTGT
>pVL17
CAGGCAGGGCTGACTCAGCCGCCCTCAGTGACCAAGTCCCCAGGACAGACGGCCACACTCACCTGCA
CTGGAAAGAGCAAAAATGTCGGCCATGAGGGGGCAGCGTGGCTGCAGCAGCAACCCCAAGGCCATGT
CCCCACACTCCTGACCAGCAGGAATAACATCTGGGCCTCTGGGGTCTCCGAGAGATTCTCTGGCTCCA
GGTGAGGCAGTGTGGCCCCCTGAGCATCTCTG
>pVL18
TACCAGCACAATGCACGGTCCCCTTCCCAGCATCTCCTCTACTACCACACGGATTCAGAGAAGCACCA
GGCTCCAGGTCCCCAGCTGCTTCTCTGGATCCAAAGATGCCTCGGCCAACGCAAGGATTCTGCTCATC
TCTGGGCAGCAGCCAGAGGATGAGGCTGATTATTATTACTGTGT
>pVL19
CAGTCTGTGCTGACTCAGCAGGCCTCAGTGTCTGGGAATCTGGGCCAGAGGGTCACCATCTCCTGCAC
TGGAAGCAGCTCCGACATCAGTGATTATGATGTGCACTGGTACCAACAGCTCCCGGGAATGGCCCCCA
AACTCATCATCTATGATAACAGCAAAACGGCCATCTGGGGTCCCAGAAGGATTCTCTGGCTCCAAGTC
TGGCAACTCGGCCACCCTGACCATCACTGGGCTCAAGTCTGAGGACGACGCTGATTATTACTG
>pVL20
CAGCCTGTGTGACTCAGTTACTCTCTGCGTCTGCCTCCCTGGGAGCTTTGGCCATGCTCAGCTGCACCC
TGAGCAGTGAGCAGAGCACCTTCTACATCGGATGGTGTCAACAGCACCCTAGGAAGGCCCCTCAGGA
TGTGACGTGCCTGAAGAGTGAAGGAAGCCACAGCAAGGGGGACGGAATTCCTGATCATTTCTGGGGC
TCCAGCTCTGGGGCTGACCGCTACTCAACCATCCCCACCAGCCAGTCTGTGGAGCATCTCACAGCATT
GCTGT
>VL21-ORF
CCGGCCAGTGCTGACTCAGCCACCTTCCCCTCTGCATCACCTGGAGCAAATGCCAGACTCACCTGCAC
TGCCAGCAGTGACATCAGTGTTGGTGGATACAGGATATTCTTCTGGACCAGCCAAAGCCAGGGAGCCC
CCAGGTATCTCTCTTGCTCTGCCACACAGACTCAAATGAGCATCAGGGCCCAGGATCCCAGCCACTTC
TCTGGATCCAAAGATGACACAACTAACTCTGGTGTTCTGCTCGCCTCCGGGCTGCAACCTGAGGACGA
GGCTCACTATTACTGT
>pVL22
CAGTCTGTGGCTCAGCCCGCCTCAGTGTCTGGGACCCTGGGCCAGACAGTCACCATCTCTTGCACTGG
AAGCAGCTCCAACATCGGGCGTGGTTATGCGGGCTGGTACCAACAGATCCCAGGAACGTGCCCCAAA
ACCGTCATCTATGCTACTAACAAATGACCCTCAGGGGTCCCAGACCGATTCTCTGTCTCCAAGTCTGGC
AACACAGCCACCCTGACCATTACTGGGCCGCAGGCTGAGGACAAGGCTGATTGTTACTGT
>pVL23
ACCTTCTCCTGCACAGGGAGCAGCACAGACATTGGGGGTCACCATGGCAACGGTACCAAGGACTCCC
ATGCAGAACCCCTCTGTGTGTGACCTAGGACGGAGGTCATTGACCTGCCAAGAGCTTGAACCTGATTC
TCCAGGTTCTGTTTGTTAACAGAACCGCTTTCACCACCTGTGCTGCAGCCTGAGGACCAGGCTGATTAT
CATTGC
>VL24-ORF
GAGTTTGTGCTGACACAGCCCCGCTCTGTGTCTGAGTCGCTGGGACAGAAGGCCACCATTTCCTGCAC
CGGCAGCAGCGGCAACATTGGAAGCAAATATGTGCACTGGTACCAGCAGCGCCCGGGCAGTACCCCC
ACCACTGTGATCTATAATGATGACGAAACACCCTCTGGGGTTCCTGATCGGTTCTCTGGCTCCATCAAC
AGCTCCTCCAATGCTGCCTACCTGACCATCTCTGGGCTGCAGCCTGAGGACCAGGCAGACTACTACTG

T
>pVL25
CTGCCTGTGCTGACCCCGCCCCCATCTGCATCTGCCTCCCTGGGAGCCTCAGCCAAGCTCACCTGCACC
CTGAGCACCTACTCCATTCACTGTATCAACAGAGACCAGGGAAGGACCCTGCGGAGGTGAAGAAGGT
TAACAGAGATGGAAGCCACAGCAACGGGAATGGACCCTTGACCACTTCTCAGGCTCCAGCCCTGTG
GCTGACTCCTACTTCACCGTCTCCAGCATCCAGTCTGAGGACGAGGCTGAGCATCACTGTG
>pVL26
CTGACTCAGCCTCCTGAGGCATCTGGGGCCTCTGGGCAGATAGTCACTATCTGCTGTGCTGAAGCAAC
AGTGACATTGAGGGTTGTAATTGTGTCTGCTGTAAACACGCTCCTGGCACAGCCCCACAAGATTCAGG
TCAGATCCCAAATCAAACCTCACAGGCCCCAGACTCCAAATCCGGCAGCATGGCCTCTCTGAGCATCC
TTCAGCTCCAGCCCAGCTGTGAGGCTGATGTTTGTTGCT
>pVL27
TCCTATGAGCTGAGGCAGCTGCCCTCAGTGTAGGTGGCCCTGGGATAGACGGCCACAATCGCCTGCCC
TGGAAATGACCTGGGGGATAAATATGCATCTTGGTCCCAGCAGAAGCCAGGCCAGGCCCCTGTGGTG
GTCATCAATAACAATGGCTGACAGCTCTCAGAGGTCCCTGCCCAATTATCTGACTCCAACTCAGGGAA
CAAGGCCACCCTGACACCCCAGCAGGGCCCAGGCCAAGGACGAAGCTGACGATTACCA
>pVL28
TCCTATGAGCTGAGGCAGCTGCCCTCAGTGTAGGTGGCCCTGGGATAGACGGCCACAATCGCCTGCCC
TGGAAATGACCTGGGGGATAAATATGCATCTTGGTCCCAGCAGAAGCCAGGCCAGGCCCCTGTGGTG
GTCATCAATAACAATGGCTGACAGCTCTCAGAGGTCCCTGCCCAATTATCTGACTCCAACTCAGGGAA
CAAGGCCACCCTGACACCCAGCAGGGCCCAGGCCAAGGACGAAGCTGACGATTACCAG
>VL29-ORF
CAGCTGGTGCTGAATCAGCCACCATCTGTGTCCTCATCCCTTGGAACCACAGTCCGCCTGGCCTGCACC
TTGAGCAGCGACCATGATGTCAGCCTTTACAGCATCTACTGGTACCAGCAGAGGCCTGGCCACCCTCC
ACGATTCTTGCTGAGATATTTCTCCCATTCAGACAAGAGCCAGGGCCCCAGGATCCCCCCTCGCTTCTC
TGGATCCAAAGACTTGGCCAAGAACACAGGGTATTTGAGCATCTCCGAGCTGCGGCCTGAGGATGAG
GCCATGTATTTCTGT
>pVL30
GTGCTTACTCAGCTGCCCTCCCTCTCTGTAGCCCTGGGAACAACAGGCCAAACTTGCCTGTACGCTGA
GCCATGGCTTCTGGGTTGGTGGCTACAGGATGTTCTAGCACAAGCAGAAACCAAGGAGCCCTCCTAGT
TTCTCCTGTCCCTTTCCTCAGATTCAGGGAAGCTCCTGACTCTGGGTCCTCAATCGCTCTTCTGGACCTA
AAGATGC
>VL31
CAGTCAGTGCTGACTCAGCCGGCCTCAGTGTCTGGGAACCTGGGCCAGAGGGTCACCATCTCCTGCAC
TGGAAGCAGCTCCGACACCAGGGATAATTATGTGAACTGGTACCAGCAGCTCCCAGGAACCGCCCCC
AAACTCATCATCTATGAAAATAGCAAAAGACCCTCTGGGATCCCAGATCGATTCTCTGGCTCCAAGTC
TGGAAACCCGGCCTCCCTGACCATCACTGGGCTCCAGGCTGAGGATGCGGCTGATTATTACTGC
>VL32-ORF
CGGCCTGTGCTGACCCAGCCGCCCTCCCTCTCTGCATCTCCTGGAACATCAGCCAGACTCTCCTGCACC
CTGAGCAGGGGCATCAGTGTTGGCAGCAAAGATACATTTTGGTACCAGCAGAAGCCAGGGAGCCCTC
CCCAGTGTCTCCTCTACTACTGCACAAACTCAGATAAACACCAGGGCTCCAGGTCCCCAGCTGCTTCTC
TGGATCCAAAGATGCCTCAGCCAACGCAGGGCTTCTGCTCATCTCTGGGCAGCAGCCCAAGGATGAGG
CTGACTATTACTGTG
>VL33-ORF

CAGTCAGTGCTGACTCAGCCGGCCTCAGTGTCTGGGAACCTGGGCCAGAGGGTCACCATCTCCTGCAC
TGGAAGCAGCTCCGACACCAGGGATAATTATGTGAACTGGTACCAGCAGCTCCCAGGAACCACCGCC
CCAAACTCATCATCTATGAAAATAGCAAAAGACCCTCTGGGATCCCAGATCGATTCTCTGGCTCCAAG
TCTGGAAACCCGGCCCTCCCTGACCATCACTGGGCTCCAGGCTGAGGATGCGGCTGATTATTACTGC
>VL34-ORF
CGGCCTGTGCTGACCCAGCCGCCCTCCCTCTCTGCATCTCCTGGAACATCAGCCAGACTCTCCTGCACC
CTGAGCAGGGGCATCAGTGTTGGCAGCAAAGATACATTTTGGTACCAGCAGAAGCCAGGGAGCCCTC
CCCAGTGTCTCCTCTACTACTACTACTGCACAAACTCAGATAAACACCAGGGCTCCAGGTCCCCAGCT
GCTTCTCTGGATCCAAAGATGCCTCAGCCAACGCAGGGCTTCTGCTCATCTCTGGGCAGCAGCCCAAG
GATGAGGCTGACTATTACTGTG
>pVL35
GTGCTTACTCAGCTGCCCTCCCCTCTCTGTAGCCCTGGGAACAACAGGCCAAACTTGCCTGTACGCTGA
GCCATGGCTTCTGGGTTGGTGGCTACAGGATGTTCTAGCACAAGCAGAAACCAAGGAGCCCTCCTAAT
TTCTCCTGTCCCTTTCCTCAGACTCAGGGAAGCTCCTGACTCTGAGTCCTCAATCGCTCTTCTGGACCA
AAAGATGCT
>pVL36
CAGGCAGGGCTGACTCAGCCGCCCTCAGTGACCAAGTCCCGGGACAGACGGCCACACTCATCTCCACT
GGAAACAGCAACAGTGGTGGCAGTGAGGGGGCAATTTGCCTGCAGCAACCCCAGCGCCTTCTTCCCA
AACTGCTGATGAGGAGGAAGAAAAATCAGGCCTCCAGAACCTCTGAGAAATTCTCTGTCTCTGGGCCA
GGCAGTGTGGCCTCCTGAGCATCTCTGGTCTGGTGTGAATGTGGGGAAACTGAATCACTCGGA
>VL37-ORF
CAGCCGGTGCTGAATCAGCCACCGTCTGTGTCCTCGTCCCTTGGAAGCACAGTCCGCCTGGCCTGCAC
CTTGAGCAGCGACCACGATGTCAGCCTTTACAGCATCTACTGGTACCAGCAGAGGCCTGGCCACCCTC
CACGATTCTTGCTGAGATATTTCTCCCATTCAGACAAGAGCCAGGGCCCCAGGATCCCCCCTCGCTTCT
CTGGATCCAAAGACTTGGCCAAGAACACAGGGTATTTGAGCATCTCCGAGCTGCAGCCTGAGGATGA
GGCCGTGTATTTCTGT
>pVL38
GTGCTTACTCAGCTGCCCTCCCTCTCTGTAGCCCTGGGAACAACAGGCCAAACTTGCCTGTACGCTGA
GCCATGGCTTCTGGGTTGGTGGCTACAGGATGTTCTAGCACAAGCAGAAACCAAGGAGCCCTCCTAAT
TTCTCCTGTCCCTTTCCTCAGACTCAGGGAAGCTCCTGACTCTGAGTCCTCAATCGCTCTTCTGGACCA
AAAGATGC
>pVL39
CAGACTGTGCTGACTCAGTCACCCTCTGCATCTAACACCCTGGGAGTTAGTCTTGGCTGAGCTCCCCTT
GCATCCAGAGTGAGCGCAGCTCCAACAGCACTGCATCGTATCAGCAGCAGCAGGGAAGGCCCCTCAG
TTTGTGATGTGGCTTAAGAGTGACAGGAGCCCCAGCAAGGGGGATGCGACCCCCAAACACTTCTCAG
GCTCCAGCTCTGGGGCTGGCCACTACTTACCCATCTCCAAAGTCCGGCCTGAGGATGAGGCTGACTCT
ATCTGT
>VL40
CAGTCAGTGCTGACTCAGCCGGCCTCAGTGTCTGGGAACCTGGGCCAGAGGGTCACCATCTCCTGCAC
TGGAAGCAGCTCCAACACCAGGGATAATTATGTGAACTGGTACCAGCAGCTCCCAGGAACCGCCCCC
AAACTCATCATCTATGAAAATAGCAAAAGACCCTCCGGGACCCCAGATCGAATCTCTGGCTCCAAGTC
TGGAAACCCGGCCTCCCTGACCATCACTGGGCTCCAGGCTGAGGATGAGGCTGATTATTACTGC
>VL41-ORF
GGCCTGTGCTGACCCAGCTGCCCTCCCTCTCTGCATCTCCTGGAATATCAGCCAGACTCTCCTCCACCC

TGAGCAGGGGCATCAGTGTTGGCAGCAAAGATACATTTTGGTACCAGCAGAAGCCAGGCAGCCCTCC
CCAGTGTCTCCTCTACTGCTACACAAACTCAGATAAACGCCAGGGTTCCAGGTCCCCAGCTGCTTCTCT
GGATCCAAAGATGCCTCAGTCAATGCAGGGCTTCTGCTCATCTCTGGGCAGCAGCCCAAGGATGAGGC
TGACTATTACTGTG
>pVL42
TTTGTGCTGACACAGCCCCGCTCTGTGTCTGAGTCGCTGGGACAGAAGGCCACCATTTCCTGCACCGG
CAGCAGCGGCAACATTGGAAGCAAATATGTGCACTGGTACCAGCAGCGCCCGGGCAGTACCCCCACC
CCCCACTGTGATCTATAATGATGACGAAACACCCTCTGGGGTTCCTGATCGGTTCTCTGGCTCCATCAA
CAGCTCCTCCAATGCTGCCTACCTGACCATCTCTGGGCTGCAGCCTGAGGACCAGGCAGACTACTACT
GT
>pVL43
ACCTTCTCCTGCACAGGGAGCAGCACAGACATTGGGGGTCACCATGGCAACGGTACCAAGGACTCCC
ATGCAGAACCCCTCTGTGTGTGACCTAGGACGGAGGTCATTGACCTGCCAAGAGCTTGAACCTGATTC
TCCAGGTTCTGTTTGTTAACAGAACCGCTTTCACCACCTCTGCTGCAGCCTCAGGACCAGGCTGATTAT
CATTGC
>pVL44
CAGGCAGGGCTGACTCAGCCGCCCTCAGTGACCAAGTCCCCGGGACAGACGGCCACACTCATCTCCAC
TGGAAACAGCAACAGTGGTGGCAGTGAGGGGGCAATTTGCCTGCAGCAACCCCAGCGCCTTCTTCCCA
AACTGCTGATGAGGAGGAAGAAAAATCATCAGGCCTCCAGAACCTCTGAGAAATTCTCTGTCTCTGGG
CCAGGCAGTGTGGCCTCCTGAGCATCTCTGGTCTGGTGTGAATGTGGGGAAACTGAATCACTCGGA
>VL45-ORF
CAGCCAGCCCTGGCCCACCGGGAATCACTGATGGTCTTCCCAGGCCAAGTGGCCCAACTCTCCTGCAC
GATCAACCCCCGCCACCCCATTGGGGACTACGGAGTGTCTTGGTACCAGCAGCGGGCAGGCAGCGCC
CCTCGCTACCTCCTCTACTACCGCTCAGAAAAGGACCACCACCGATCCCCTGACATCCCTGACCGCTTC
TCAGCAGCCGCCGACGCAGCCCACAACGTCTGCATCCTGACCATCAGCCCTGTGCAGCCCGAGGACGA
CGCGGATTATTACTGC
>pVL46
CAGGCTGTGCTGACTCAGCTGGCCTCAGTGTTGGGGTCCCTGGGAGACGTGGGCACCATAGCCTGCAC
ATGAAGAAGCAACATTGGGCCATATGCCGCAGACTGGACCAACATCTGCCGGGTCTCCAGAGTCCTCA
TCCACAAGAATGACAATCGGCCCTTAGGGATCCTCCCAAGGTGGTTCTTTGGCTCCAAGTTGGGCCAC
ACAGCCTTCCCCACCACTGCTGGGCTCCAGGCTGAGGACAAGGCCAGTTATAACCGT
>pVL47
CAGGCAGGGCTGACTCAGCCGCCCTCAGTGACCAAGTCCCCGGGACAGACGGCCACACTCATCTCCAC
TGGAAACAGCAACAGTGGTGGCAGTGAGGGGGCAATTTGCCTGCAGCAACCCCAGCGCCTTCTTCCCA
AACTGCTGACAAGGAGGAAGAAAAATCAGGCCTCCAGAACCTCTGAGAAATTCTCTGTCTCTGGGCC
AGGCAGTGTGGCCTCCTGAGCATCTCTGGTCTGGTGTGAATGTGGGGAAACTGAATCACTCGGA
>pVL48
CTGGAAGCAGCAGTGACATTGGATCCTATAACATCTATGGTTCCAGCTCTTACTGGTATTGCCCCAAA
ACTCTGACTCTTGGAAGTACCCAGCCCTGAGAGATTCCAGATGAGTCTGTCTCTAAGTCAGGCAACTT
CCCCTCTCTTACCACCTGTGGGCTCCGGCCTGAGGATGAGGTGATTCTTACTGT
>pVL49
CAGGCAGGGCTGACTCAGCCGCCCTCAGTGACCAAGTCCCCGGGACAGACGGCCACACTCATCTCCAC
TGGAAACAGCAACAGTGGTGGCAGTGAGGGGGCAATTTGCCTGCAGCAACCCCAGCGCCTTCTTCCCA
AACTGCTGATGAGGAGGAAGAAAAATCAGGCCTCCAGAACCTCTGAGAAATTCTCTGTCTCTGGGCCA

GGCAGTGTGGCCTCCTGAGCATCTCTGGTCTGGTGTGAATGTGGGGAAACTGAATCACTCGGA
>pVL50
CAGTCTGGGTGACACAGGAAGCCTCAGTGTCTGGGTCTGTGGGACAGAAAGTCACCCTCTCCTGCGCT
GGAAATAGCAACAACGTTGGAATATTTGATGTGGGCTGGTACCGTATTTCTCACGGTGCCCCCAAAAC
TGTGATGCTCAGAAGTTCTCGTCCCTCAGGGATCCCAGCTCGGTTCTCTGGCTCCAAGTCTGGCAACAT
GGCCTATCTGACCATCTCTGGGCTCCAGCCTGAGGATGAGGCTGATTATTACTGTT
>pVL51
CAGCACCCATATCTGTCTGTGTCTTCAGGTTCCCTCTCCCAGCCTGTGCTGACCCAGCCGCCCCTCCCT
CTCTGCATCTCCTGGAACAACACAGCCAGACTCACCTGCACCCTGAGCAGGGACGTCAGTGTGGGCAT
CTATCCCAGTCTGTGCTGCTACTCAGACTCAAGCATGTGATTGGGATCAGCAGATCCCAGCTGCTTCTC
TGGATCCAAAGATGCCTCGGCCAATGCAGGCTTTTGCTTATCTCTGGGCTCCAGCCTGAGAACGAGG
CTGACTCTTACCAT
>pVL52
GTCACCTTCTCCTGCACAGGGAGCAGCAGAGACATTGGGTGTCACCATGGCAAGGTACCAAGGACTCT
CATGCAGAGCCCCTACTTGTGTGACCTAGGACAGCAGTGGGGACCTTCCAGGGGTTCCAGACTGATTC
TTGAGATCCACATTTATTACTAGAACCTCTTTGGCCACCTCTGGGCTCCAGCCTGAGGATGAGGCTGAT
TATCACTGT
>VL53
CAGACGGTGGTGACCCAGGAGCCATCACTCTCAGTGTCTTCAGGAGGGACGGTCACACTCACCTGTGG
CCTTAGCTCCGGGTCAGTCTCTTCAGGTTATGACCCCAGCTGGTACCAGCAAACCCCAGGCCAGGCTC
GCTGCACACTCATCTACAGCACAAACAGCCGCCTCTCTGGTGTCCCTAATCACTTCTCTGGATCCGTCT
CCAGGATCAAAGCCACCCTCACCACCACGGGGGCCCAGCCTGGGGACGAGCCTGACTATTACTGT
>VL54-ORF
CAGCCTGTGCTGACTCAATCTTCCTCCATTTCTGCATCCCCAGGGTCTTATGCTACACTCACGTGCACC
CTGAGCGCTGGCTTCAGTGTGGGCAGCTCTGCCATACACTGGTTCCAGCAGGAGACAGTGAGCCCTCT
CTGGTTTCTCCTGGGACTTAGATCAGACTCAGATAATCACCAGGGCTTGGGGGCCCCAGCCACTTCTCT
GGATCCAAAGGTACCTTGGCCAATGCAGGGCTTCTGCTCATCTCTGGGTTCCAGTCTGAGGATGAAGC
CAATTATTTATCCT
>pVL55
GCTGTGCTGACTCAGCTGCTCTCCCTCTCTGCATCTCCCAGAACATCAGCCAGGCTCTCCTTCACCCTG
AGCAGTGACATCAATGTTGGCAGCAAGAATACATTCTGGTACCAGTAGAAGCCAGGGACCCCTCTCCA
CTATCTCCTCTACTACCACACAGACTCAGATAAGCACCAGGGCTCCAGGTCCCTAGCCACTTCTCCGG
ATCCAAAGACGTCTTGGTCAATGCAGGGCTTCTGCTCATCTCTGAGCTGCAGCCTAAGGACAAGGCTG
ACTATGACTGTG
>VL56
CAGTCTGTGACTCAGCCCGCCTCAGTGTCTGGGACCCTGGGCCAGACAGTCACCATCTCCTGCACTGG
AAGCAGCTCCAATGTCGGGAGTGGTTATGTGGGCTGGTACCAACAGATCCCAGGAAGAGCCCCCAAA
CTCCTCATCTATTATGCCACTAATAGGGCTTCTGGGGTCCCCGACCGATTCTCTGGCTCCAGGTCTGGC
AACACAGCCACCCTGACCATCTCTGGGCTCCAGGCTGAGGACGAGGCCGATTATTACTGT
>VL57
GAGGCTGTGCTGACTCAGCCTCACTTGGTGTCAGAGTCCCTGGGGCAGAAGGCCACCATCTCCTGCAC
TCTCACCAGCGGTGACATTGGAGACAACTATGTGCACTGGTACCAGCAGCACCCAGGCAGTGCCCCCA
CCACCATGATCTACAAGGATGACCGAAGACTCCCAGGGTTCCTGATCAGTTCTCTGGCTGCAGTGACA
GCTCCTCCTATGCTGCCTACATGGCCATCTCCGAGCTGCAGCCTGAGGATGAGGCTGACTAGGACTGT

C
>pVL58
CAGCCTGTGCTGACTCAGCTGCTCTTCCTCTCTACATGTCCCAGAACATCAGCCAGAATCACCTGCACC
CTGAGAAGTGACATCAGTGTTGGCAGCAAAGATACATTCTGGTACCAGCAGAAGCCAGGGACCACTC
CCCCGTCTCCTCTACTACCACACGGACTCAGATAAGCACCAGGGCTCCAGGTCCCCAGCTGCTTCTCTG
GATCCAAAGATGCCTTGACCAATGCAAGGCTTCTGCTCATCTCCAGGCAGTAGCCTGAGGACGAGGCT
GACTATTCGTGTGCA
>pVL59
CAGCCTGTGCTGACTCAGCCATCCTCCCTCTCCACATCTCTGGGAACAACAGCCAGACTCACCTGCAC
CATGAGCAGTGGTTTCAGTATTGGCGACTACTGGGTACACTGGTTCCAGCAGAAGCCAGGGAGCCCTG
CTCAGTGTCTCCTGTACTACTAGTCAGACTCAGATATGCACCAGGGCTCTTCCCCAGTCACTTCTCTGG
ATCCAAAGATGCCTCGGCCAATGCAGGGCATCTGCTCATCTCTGGACTCCAGCCTGAGGACGAGGCTG
ACTATTACTGT
>pVL60
GCTGTGCTGACTCAGCTGCTCTCCCTCTCTGCATCTCCCAGGACATTAGGCAGACTCACCTGCAACCTG
AGAAGTGGCATCAATGTTGGCAGCAAAGATACATTCTGGTACCAGCAGAAGCCAGGGACCCGTTGCC
AGTGTCTCCTCTACTCCACACAAATTCAGATAAGCACCAGGGCTTCAGGTCCCCAGCTGCTCCTATGGT
TCCAAGGAGGCCTCAGACAATGCAGGGCTTCTGCTCATCTCTGGACTCCAGCCTGGGGATGCAGCTGA
CTATGACTG
>VL61
CAGCCTGTGCTGACCCAGCCGCCCTCCCTCTCTGCATCTCCTGGAACATCAGGCAGACTCACCTGCACC
CTGAGCAGTGATGTCAGTGTTGGCAGCTCTCTCATATTCTGGTACCAGCAGAAGCCAGGGAGCCCTCC
GGGGTATCTTCTGAGTTTCTACTCAGACTCAGTTAAGCACCAGGGCTCCCGGGTCCCCAGCCACTTCTC
TGGATCCAAAGACACCTCGGCCAATGCAGGGCTTCTGCTCATCTCTGGGCTCGAGGCTGAGGACGAGG
CTGACTATTACTGT
>pVL62
GTGCTGCCCCAGCCACCCTCAGTGTCAGAATTTCCAGGAGGAAGAATCTCCCTCAACAGGAAAACCAG
TAACATGGTGTGTGTAACTAGGAGGGGGTCCTCTTATCAGGGTCAGCCAGGAAATGCCCTGGACACAG
GCTCTATGAGGAAGACTTTGATGCTCAGGTAGCCCAGGATGAGTCTCTGCTCCCTGTCAGCAGCTCAG
CCTCCCTGACCATCTCTG
>VL63
CAGTCTGTGACTCAGCCCCCCTCAGTGTCTGGGACCCTGGGCCAGACAGTCACCATCTCCTGCTCTAG
AAGCAGCTCCAACATCGGGTATAGTAGTAGTTATGTGAGCTGGTACCAACAGATCCCAGGAACAGTCC
CCAAAACCCTCATCTATTATGCCACTAGTAGAGTGTCCGGGGTCCCCGATCGATTCTCTGGCTCCAGGT
CTGGCAACACAGCCACCCTGACCATCACTGGGCTCCAGGCTGAGGACGAGGCTGATTATTACTGC
>pVL64
CAGCCTGTGCTGACTCAGTTGCCACCTGCAACTGCCTCCCTGGGAGCCTGGGCTGAGATCACCTGCAT
CCAGAGCGGGGAGCACAGCACTGACAGGTCTCCATCGTATCTGCAGCAGACGGGGAAGGCCCTCAGT
TTGTGACATGGCTTAAAAGTGACAGGGGCAACAGCAAGCGTTATGGGATCCCAAGTCACTCCCCAGG
CTCCAGCTCTGGGGCTGGCCACTATTTACCCATCTGCAAAGTCCGGTCTGAGGTTGAAGGCTGACTCC
ATCCG
>pVL65
CAGTCTGTGACTCAGCCCGCCTCAGTGTCTGGGACCCTGGGCCAGACAGTCACCGTCTCCTGCTCTGG
AAGCAGCTCCAACATCGGGGTGGTTATGGTACGAGCTGGTTCGAACAGATCCCAGGAACAGCCCCCA

AAATGCTCATCTATGCTACTAACACACGTCTCTCAGGGGTCCCAGATCGATTCTCTGGCTCCAAGTCTG
ACAACACAGCTACCCTGACCATCACCGGGCTCCAGGCTGAGGACGAGGCTGATTATTACTGAT
>pVL66
CATTCTGTGCTGACTCAGCCATCCTCCCTCTCCACATCTCTGGGAGCAACAGCCAGACTCACCTGCACC
ACGAGCAGTGGTTTCAGTGTTGGCGACTACTGGGTACACTGGTTCCAGCAGAAGCCAGGGAGCCCTGC
TCAGTGTCTCCTGTACTACTAGTCAGACTCAGATATGCACCAGGGCTCTGAGGTCCCCAGTCACCTCTC
TGGATCCAAAGATGCCTCGGCCAATGCAGGGCTTCTGCTCATCTCTGGACTCCTGCCTGAGGACGAGG
CTGACTATTACTGT
>VL67
CAGCCTGTGCTGACTCAGCCATCCTCCCCTCTCCACATCTCTGGGAACAACAGCCAGACTCACCTGCA
CCATGAGCAGTGGTTTCAGTATTGGCGACTACTGGGTACACTGGTTCCAGCAGAAGAAGCCAGGGAG
CCCTGCTCAGTGTCTCCTGTACTACTAGTCAGACTCAGATATGCACCAGGGCTCTTCCCCAGTCACTTC
TCTGGATCCAAAGATGCCTCGGCCAATGCAGGGCATCTGCTCATCTCTGGACTCCAGCCTGAGGACGA
GGCTGACTATTACTGT
>pVL68
CAGTCTGTGCTGACTCAGCCACCATCAGTATCTGGGGCCCCAGGCCTGAGGATCACCATGTACTGCAA
AGGAAGCAGCTTCAACATCGGGGGTGGAAATTATGTGTCCTGGTACCAACCGCTCCAGGGAACAACC
CCCAGACTTCTGACATATGAGAACAGCAAACACCCCTCTGGGGTCCCTGATAGATTCTCTTGATCCTA
GTCTGGCAACTCCGGATCACTGACCATCACTGGGCTGCAGATTGGGGACGAAGCTGATTATTACTGC
>pVL69
CAGCCTGTGCTGACTCAGCTGCTCTTCCTCTCTACATGTCCCAGAACATCAGCCAGAATCACCTGCACC
CTGAGAAGTGACATCAGTGTTGGCAGCAAAGATACATTCTGGTACCAGCAGAAGCCAGGGACCACTC
CCCCGTCTCCTCTACTACCACACGGACTCAGATAAGCACCAGGGCTCCAGGTCCCCAGCTGCTTCTCTG
GATCCAAAGATGCCTTGACCAATGCAAGGCTTCTGCTCATCTCCAGGCAGTAGCCTGAGGACGAGGCT
GACTATTCGTGTGCA
>VL70-ORF
CAGCCTGTGCTGACTCAGCCTTCCTCCATTTCTGCATCCTCTGGATCTTACGCTACACTCACCTGCACC
CTGAGCCGTGGCTTCAGTGTGGGCAGCTCTGCCATACACTGGTTCCAGCAGGAGACAGTGAGCCCTCT
CTGGTTTCTCCTGGGATTTAGATCAGACTTAGATAAGCACCCGGGCTCAGGGATTCCCAGCCACTTCTC
TGGATCCAAAGGTACCTCGGCCAATGCAGGGCTGCTGCTCATCTCTGGGCTCCAGTGTGAGAATGAGG
CCAGTTATTACTGC
>pVL71
TCCTCCCTCTCTGTTCCCTCTAAACCTCTGTGAGACTCACCTGCACCATGAGCAGGGGCTTCACTCTTG
GTACCTTCTGGATATGCTGGTACCGGCAGAAGCCAAGGAGTCCTCCCAGGTGTTTTCGTACGTTCCACT
CACATTCAGATGAGCACCAGGGCTCTGGGGGTCCCCAGGAAATTCCCCGGATCCAATGATGCATCAGC
CAATGCTAGGGTTTTGCTCATCTCTGCACTCCAG
>VL72
CAGTCTCTGACTCAGCCCGCCTCAGTGTCTGGGACCCTGGGCCAGACAGTCACCATCTCCTGCTCTGG
AAGCAGCTCCAACATCGGGTATGGTAGTAGTACTGTGGGCTGGTTCCAACAGATCCCAGGAACAGCCC
CCAAAACCCTCATCTATGCTACTAACAAACGAGCCTCAGGGGTCCCAGATCGATTCTCTGGCTCCAAG
TCTGGCAACACAGCCACCCTGACCATCTCTGGGCTCCAGGCTGAGGACGAGGCCGATTATTACTGT
>pVL73
CAGCCTGTGCTGACTCAGCCACCCTCCCTTTCTGCATCTCCTGGATCATCGGCCAGACTCAACTGCACC
CTGAGCAGTGACGTCAGTCTTGGCAGCTATACCATATACTGGTAGCAGCAGAAGCCAGGGAGCCCTCC

CCAGTGTCTCCTGAGTTTCTTCTCAGATTCATAAAAACATCAAGGCTTCGGGGGTCCCCAGCCGCTTCT
CTGGGTCCAAAGATTCCTCTGCCAATGCAGGGCTCCTGCTCATCTCTGGACTGCAGCCTGAGGATGAG
GCTGACTATTACTGTG
>pVL74
CAGTGTGTGACTGAGCCCGCCTCAGTGTCTGGGACCCTGGGCTCGACAGTCACCATCTCCTGCTCTGG
AAGCAGCAGCAACATTGGTGGTTATGGTGTTGGCTGGTTCCAACAGATCCAAGGAAAAGCCCCCAAA
ACCATCATCTCTGCTAATAACAAACAACCCTCAGGGATCCCAGATCGATTTTCTGGCTCCAAGTCTGG
CAACACAGCCACCCTGACCATCTCTGGGCTCCAGGCTGACGACGAGGCCGATTATTACTGT
>pVL75
CAGCCTTTGCTGACTCAATTGCCACCTGCAACTGCCTCCCTGGGAGCCTGGGCCGAGATTACCTACATC
CAGAACAGGGAGCACAGCACCGGCAGGTCTGCGTCACATCAGCAGCAGATGGGGAAGGCCCCTCAGT
TTGTGACATGGTTTAAAAATGACAGGAACCACAGCAAGGGGGATGGGATCCCCAGTCACTTCTCAGGT
TCTAGCTCTGGGGCTGACCACTAAGTAACCATCTCCGGTCTGAGGATGAGGCTGAGTCCCTCTGTGGA
ATAG
>pVL76
CAGCCTGTGCTGACTCAGCCACCCTCCCTTTCTGCATCTCCTGGATCATCGGGCAGACTCAACTGCACC
CTGAGCAGTGAGTGACGTCAGTCTTGGCAGCTATACCATACACTGGTACCAGCAGAAGAAGCCAGGG
AGCCCTCCCCAGTGTCTCCTGAGTTTCTTCTCAGATTCATAAAAACATCAAGGCTTCGGGGTCCCCAGC
CGCTTCTCTGGGTCCAAAGATTCCTCTGCCAACGCAGGGCTCCTGCTCATCTCTGGACTGCAGCCTGAG
GATGAGGCTGACTCTTACTGTG
>pVL77
CAGTCTGTGCTGACTCAGCCACCATCACTCTCTGGGGCCCCAGGCCAGAGGGTCACCATGTACTGCAC
CGGAAGCAGCTCCAACATCGGGGGTGGCAATTATGTGTCCTGGTACCAACAGCTCCAGGGAACGACC
CCCATACTCCTGACATAGAAGAACAGCAAACACCCCTCTGGGGTCCCTGATCGATTCTCTTGATCTGA
GTCTGGCAACTCCGGATCCCTGACCATCACTGGGCTCCAGATTGGGGACGAGGCTGATTATTACTGC
>pVL78
CAGTCTGTGCTGACTCAGCCACCATCACTCTCTGGGGCCCCAGGCCAGAGGGTCACCATGTACTGCAA
CGGAAACAGCTCAACATCGGGGGTGGCAGTTATGTGTCCTGGTACCAACAGCTCCAGGGAACGACCC
CCAACTCCTGACATATGAGAAACAGCAAACACCCTCTGGGGTCACTGGTCACTGATCAATTCTCTTGA
GCTCTGATCTAGTCTGGCAAACTTCGGGATCACTGACCATCACTGGGCTCCAGATTGGGGACGAGGCT
GATTATTACTGCTT
>pVL79
CTGGAAGCAGCAGTGACATTGGATCCTATAACATCTATGGTTCCAGCTCTTAGTGGTATTGCCCCAAA
ACTCTGACTCTTGGAAGTACCCAGCCCTGAGAGATTCCAGATGAGTCTGTTCTCTAAGTCAGGCAACT
TCCCCTCTCTTACCACCTGTGGGCTCCGGCCTGAGGATGAGGTGATTCTTACTGT
>pVL80
CAGGCAGGGCTGACTCAGCCGCCCTCAGTGACCAAGTCCTCGGGACAGACGGCCACACTCACCTGCA
CTGGAAACAGCAACAATGGTGGCAGTGAGGGGGCAATTTGCCTGCAGCAACCCCAGCGCCTTCTTCCC
AAACTGCTGATGAGGAGGAAGAAAAATCAGGCCTCCAGAACCTCTGAGAAATTCTCTGTCTCTGGGCC
AGGCAGTGTGGCCTCCTGAGCATCTCTGGTCTGGTGTGAATGTGGGGAAACTGAATCACTCGGA
>pVL81
CAGTCTGGGTGACACAGGAAGCCTCAGTGTCTGGGTCTGTGGGACAGAAAGTCACCCTCTCCTGCGCT
GGAAATAGCAACAACGTTGGAATATTTGATGTGGGCTGGTACCGTATTTCTCACGGTGCCCCCAAAAC
TGTGATGCTCAGAAGTTCTCGTCCCTCAGGGATCCCAGCTCGGTTCTCTGGCTCCAAGTCTGGCAACAT

GGCCTATCTGACCATCTCTGGGCTCCAGCCTGAGGATGAGGCTGATTATTACTGTT
>pVL82
GTGCTGACTCAGACCAGCTCTGTGCTGGAGTCTCCGGGGCAGGAGGCCACCATCTCCTGCACCCACAG
CAGTGGCTACATTGGGAGCAGATATGTGCACTGCTACCAGCAGTGCCCCCATCACCGAAATCTACAAG
AGTGACAAGGGAGCCTCCAGGGTTCCTGATCAGTTTTCTGGTTCCATAGGAAGCTCCCCTAATGCCAC
CTGCCTGACCGTCTCTGAGCTGCAGCCTGAGGACGAGGCCAACTACTCCGGT
>pVL83
CAGCCTGTGCTGACCCAGCCGCCCTCCCTCTCTGCATCTCCTGGAACAACAGCCAGACTCACCTGCAC
CCTGAGCAGGGACGTCAGTGTGGGCATCTATCCCAGTCTGTGCTGCTACTCAGACTCAAGCATGTGAT
TGGGATCAGCAGATCCCAGCTGCTTCTCTGGATCCAAAGATGCCTCGGCCAATGCAGGCTTTTTGCTTA
TCTCTGGGCTCCAGCCTGAGAACGAGGCTGACTCTTACCAT
>pVL84
AAGTGTGTGCATGTGTGACTCCTCCTGTGAAGCTCCTGATGGGCCCGGATCCAAGGTCACCTTCTCCTG
CACAGGGAGCAGCAGAGACATTGGGTGTCACCATGGCAATGGTACCAAGGACTCTCATGCAGAGCCC
CTACTTGTGTGACCTAGGACAGCAGTGGGGACCTTCCAGGGGTTCCAGACTGATTCTTGAGATCCACA
TTTATTACTAGAACCTCTTTGGCCACCTCTGGGCTCCAGCCTGAGGATGAGGCTGATTATCACTGT
>VL85
CAGACGGTGGTGACCCAGGAGCCATCACTCTCAGTGTCTTCAGGAGGGACGGTCACACTCACCTGTGG
CCTTAGCTCCGGGTCAGTCTCTTCAGGTTATGACCCCAGCTGGTACCAGCAAACCCCAGGCCAGGCTC
CCTGCACACTCATCTACAGCATGAACAGCCGCCTCTCTGGGGTCCCTAATCGCTTCTCTGGATCCGTCT
CCGGGAACAAAGCCACCCTCACCACCACGGGGGCCCAGCCTGAGAACGAGGCTGACTATTACTGT
>VL86-ORF
CAGCCTGTGCTGACTCAATCTTCCTCCATTTCTGCATCCCCAGGGTCTTACGCTATACTCACCTGCACC
CTGAGCGCTGACTTCAGTGTGGGCAGCTCTGCCATACACTGGTTCCAGCAGGAGACAGTGAGCCCTCT
CTGGTTTCTCCTGGGACTTAGATCAGACTCAGATAAGCACCAGGGCTCGGGGGCCCCAGCCACTTCTC
TGGATCCAAAGGTACCTTGGCCAATGCAGGGCTTCTGCTCATCTCTGGGTTCCAGTCTGAGGATGAAG
CCAATTATTTATCCTGAGCTAACATCTACCAATCCTCCT
>pVL87
CAGCTGTGCTGACTCAGCTGCTCTCCCTCTCTGCATCTCCCAGAACATCAGCCAGGCTCTCCTTCACCC
TGAGCAGTGACATCAATGTTGGCAGCAAGAATATATTCTGGTACCAACAGAAGCCAGGGACCCCTCCC
CTCTATCTCCTCTAGTACCACCACACAGACTCAGATAAGCACCAGGGCTCCAGGTCCCTAGCCACTTCT
CCGGATCCAAAGACGTCTTGGTCAACTGCAGGGCTTCTGCTCATCTCTGAGCTGCAGCCTAAGGACAA
GGCTGACTATGACTGT
>pVL88
CAGTCTGTGACTCAGCCCGCCTCAGTGTCTGGGACCCTGGGCCAGACAGTCACCATCTCCTGCACTGG
AAGCAGCTCCAACATCGGGAGTGGTCATGTGTCCTGGTACCAAACAGATCCCAGGAACAGCCCCCAA
ACGCCTCATCTATTCTTCCGCTAGCAGGGCTTCCGGGGGTCCCCGACCGATTCTCTGGCTCCAGGTCTG
GCAACACAGCCACCCTGACCATCTCTGGGCTCCAGGCTGAGGACGAGGCCGATTATTACTGT
>pVL89
CAGCCTGTGCTACCTCAGCCACCCTCCCTCTGCATGTCCTGGAATACCAGCCAGACTCTCCTGCACCCT
GTGCTCTCTCGCTTCAGTGTTGATGATTACTGGATATGCTGGTACCAGAAGAAACCATGCAGCCCCTCC
CTAGGATCTCCTCATTTTCAAGTCAGACTCAGATAAGCCTCAGGGTTGTGGGGTCCGCAGCCCACTTGT
CTGGAGCTGAATACGCCTCGGCCAATGCAGGGCATTTGCTCCTCTCTGGGCTCGAGCCTGAGGATGAG
GCTGACTGTGATTGT

>VL90
CAGTCTGTGACTCAGCCCGCCTCAGTGTCTGGGACCCTGGGCCAGACAGTCACCATCTCCTGCACTGG
AAGCAGCTCCAACATCGGGAGTGGTCATGTGTCCTGGTACCAACAGATCCCAGGAACAGCCCCCAAA
CGCCTCATCTATTCTTCCGCTAGCAGGGCTTCCGGGGTCCCCGACCGATTCTCTGGCTCCAGGTCTGGC
AACACAGCCACCCTGACCATCTCTGGGCTCCAGGCTGAGGACGAGGCCGATTATTACTGT
>pVL91
GCTGTGCTGACTCAGCTGCTCTCCCTCTCTGCATCTCCCAGAACATCAGCCAGGCTCTCCTTCACCCTG
AGCAGTGACATCAATGTTGGCAGCAAGAATATATTCTGGTACCAACAGAAGCCAGGGACCCCTCCCCT
CTATCTCCTCTAGTACCACACAGACTCAGATAAGCACCAGGGCTCCAGGTCCCTAGCCACTTCTCCGG
ATCCAAAGACGTCTTGGTCACTGCAGGGCTTCTGCTCATCTCTGAGCTGCAGCCTAAGGACAAGGCTG
ACTATGACTGTG
>VL92-ORF
CAGCCTGTGCTGACTCAATCTTCCTCCATTTCTGCATCCCCAGGGTCTTACGCTATACTCACCTGCACC
CTGAGCGCTGACTTCAGTGTGGGCAGCTCTGCCATACACTGGTTCCAGCAGGAGACAGTGAGCCCTCT
CTGGTTTCTCCTGGGACTTAGATCAGACTCAGATAAGCACCAGGGCTCGGGGGCCCCAGCCACTTCTC
TGGATCCAAAGGTACCTTGGCCAATGCAGGGCTTCTGCTCATCTCTGGGTTCCAGTCTGAGGATGAAG
CCAATTATTTATCCT
>pVL93
CAGTCTGGGTGACACAGGAAGCCTCAGTGTCTGGGTCTGTGGGACAGAAAGTCACCCTCTCCTGCGCT
GGAAATAGCAACAACGTTGGAATATTTGATGTGGGCTGGTACCGTATTTCTCACGGTGCCCCCAAAAC
TGTGATGCTCAGAAGTTCTCGTCCCTCAGGGATCCCAGCTCGGTTCTCTGGCTCCAAGTCTGGCAACAT
GGCCTATCTGACCATCTCTGGGCTCCAGCCTGAGGATGAGGCTGATTATTACTGTT
>pVL94
CAGCCTGTGCTGACCCAGCCGCCCCTCCCTCTCTGCATCTCCTGGAACAACAGCCAGACTCACCTGCA
CCCTGAGCAGGGACGTCAGTGTGGGCATCTATCCCAGTCTGTGCTGCTACTCAGACTCAAGCATGTGA
TTGGGATCAGCAGATCCCAGCTGCTTCTCTGGATCCAAAGATGCCTCGGCCAATGCAGGCTTTTTGCTT
ATCTCTGGGCTCCAGCCTGAGAACGAGGCTGACTCTTACCAT
>pVL95
GTGTGCATGTGTGACTCCTCCTGTGAAGCTCCTGATGGGCCCGGATCCAAGGTCACCTTCTCCTGCACA
GGGAGCAGCAGAGACATTGGGTGTCACCATGGCAATGGTACCAAGGACTCTCATGCAGAGCCCCTAC
TTGTGTGACCTAGGACAGCAGTGGGGACCTTCCAGGGGTTCCAGACTGATTCTTGAGATCCACATTTA
TTACTAGAAACCTCTTTGGCCACCTCTGGGCTCCAGCCTGAGGATGAGGCTGATTATCACTGT
>VL96-ORF
CAGACGGTGGTGACCCAGGAGCCATCACTCTCAGTGTCTTCAGGAGGGACGGTCACACTCACCTGTGG
CCTTAGCTCCGGGTCAGTCTCTTCAGGTTATGACCCCAGCTGGTACCAGCAAACCCCAGGCCAGGCTC
CCTGCACACTCATCTACAGCATGAACAGCCGCCTCTCTGGGGTCCCTAATCGCTTCTCTGGATCCGTCT
CCGGGAACAAAGCCACCCTCACCACCACGGGGGCCCAGCCTGAGAACGAGGCTGACTATTACTGT
>pVL97
CAGCCTGTGCTGACTCAATCTTCCTCCATTTCTGCATTCCCCAGGGTCTTACGCTATACTCACCTGCAC
CCTGAGCGCTGACTTCAGTGTGGGCAGCTCTGCCATACACTGGTTCCAGCAGGAGACAGTGAGCCCTC
TCTGGTTTCTCCTGGGACTTAGATCAGACTCAGATAAGCACCAGGGCTCGGGGGCCCCAGCCACTTCT
CTGGATCCAAAGGTACCTTGGCCAATGCAGGGCTTCTGCTCATCTCTGGGTTCCAGTCTGAGGATGAA
GCCAATTATTTATCCT
>pVL98

GCTGTGCTGACTCAGCTGCTCTCCCTCTCTGCATCTCCCAGAACATCAGCCAGGCTCTCCTTCACCCTG
AGCAGTGACATCAATGTTGGCAGCAAGAATATATTCTGGTACCAACAGAAGCCAGGGACCCCTCCCCC
TCTATCTCCTCTAGTACCACACAGACTCAGATAAGCACCAGGGCTCCAGGTCCCTAGCCACTTCTCCG
GATCCAAAGACGTCTTGGTCACAATGCAGGGCTTCTGCTCATCTCTGAGCTGCAGCCTAAGGACAAGG
CTGACTATGACTGTG

>pVL99

CAGTCTGTGACTCAGCCCGCCTCAGTGTCTGGGACCCTGGGCCAGACAGTCACCATCTCCTGCACTGG
AAGCAGCTCCAACATCGGGAGTGGTCATGTGTCCTGGTACCAACAGATCCCAGGAACAGCCCCCAAA
CGCCTCATCTATTCTTCCGCTAGCAGGGCTTCCGGGGGTCCCCGACCGATTCTCTGGCTCCAGGTCTGG
CAACAACACAGCCACCCTGACCATCTCTGGGCTCCAGGCTGAGGACGAGGCCGATTATTA

>pVL100

CAGCCTGTGCTACCTCAGCCACCCTCCCTCTGCATGTCCTGGAATACCAGCCAGACTCTCCTGCACCCT
GTGCTCTCGCTTCAGTGTTGATGATTACTGGATATGCTGGTACCAGAAGAAACCATGCAGCCCTCCCT
AGGATCTCCTCATTTTCAAGTCAGACTCAGATAAGCCTCAGGGTTGTGGGGTCCGCAGCCACTTGTCT
GGAGCTGAATACGCCTCGGCCAATGCAGGGCATTTGCTCCTCTCTGGGCTCGAGCCTGAGGATGAGGC
TGACTGTGATTGT

>VL101-ORF

CAGCCTGTACTGACTCAGCCTTTCTCCATTTCTGCATCCCCAGGATCTTACAATACACTCACCTGCACC
CTGAGTGCTGCCTTCAATGTGGGGCAGCTGTGCCATACACTGGTTCCAGCAGGAGACAGTGAGCCCTC
CTTGGTTTTCTCCTGGGACTTAGATCAGACTCAGATAAGCACCAGGGCTCAGGGGGCCCCAGCCACTT
CTCTGGATCCAAAGGTACCTTGGCCAATGCAGGGCTTCTGCTCATCTCTGGGTTCCAGTCTGAGGATG
AAGCCAATTATTTATTTA

>VL102-ORF

CAGCCTGGGCTGACTCAGCTGCTCTTCCTCTCTACATGTCCCAGAACATCAGCCAGACTCACCTGCACC
CTGAGAAGTGACGTCAGTGTTGGCAGCAAAGATATATTCTGGTACCAGCAGAAGCCAGGGACCCCTC
CCCCATGTCTCCTCTACTACCACACAGACTCAGATAAGCACCAGGGCTCCAGGTCCCCAGCTGCTTCTC
TGGATCCAAAGATGCCTTGGCCAATGCCGGGCTTCTGCTCATCTCTGGGCAGCAGCCTGAGGACGAGG
CTGACTATTCGTGTG

>VL103-ORF

CAGCCTGTACTGACTCAGCCTTTCTCCATTTCTGCATCCCCAGGATCTTACAATACACTCACCTGCACC
CTGAGTGCTGCCTTCAATGTGGGCAGCTGTGCCATACACTGGTTCCAGCAGGAGACAGTGAGCCCTCC
TTGGTTTCTCCTGGGACTTAGATCAGACTCAGATAAGCACCAGGGCTCAGGGGGCCCCAGCCACTTCT
CTGGATCCAAAGGTACCTTGGCCAATGCAGGGCTTCTGCTCATCTCTGGGTTCCAGTCTGAGGATGAA
GCCAATTATTTATTT

>VL104-ORF

GAGGCTGTGCTGACTCAGCCTCACTTGGTGTCAGAGTCCCTGGGGCAGAAGGCCACCATCTCCTGCAC
TCTCACCAGCGGCGACATTGGAGGCAACTATGTGCACTGGTACCAGCAGCACCCAGGCAGTGCCCCCA
CCACCATGATCTACAAGGATGACCAAAGACCCACAGGGTTCCTGATCAGTTCTCTGGCTGCAGTGACA
GCTCCTCCCATGCTGCCTACCTGGCCATCTCTGGGCTGCAGCCTGAGGATGAGGCTGACTAGGACTGT
C

>pVL105

CAGCCTGGGCTGACTCAGCTGCTCTTCCTCTCTACATGTCCCAGAACATCAGCCAGACTCACCTGCACC
CTGAGAAGTGACGTCAGTGTTGGCAGCAAAGATATATTCTGGTACCAGCAGAAGCCAGGGACCCCTC
CCCCATGTCTCCTCTACTACCACACAGACTCAGATAAGCACCAGGGCTCCAGGTCCCCAGCTGCTTCTC

TGGATCCAAAGATGCCTTGGCCAATGCCGGGCTTCTGCTCATCTCTGGGCAGCAGCCTGAGGACGAGG
CTGACTATTCGTGTG
>pVL106
CAGACTGTGCTGACTCTGCCACCATCAGTATCTGGGGCCCCAGGCCTGAGGGTCACCATCTACTGCAC
TAGAAGCAGCTCCAACATCAGGGGTGGCAATTATGTGTCCTGGTACCAACAGCTCCAGGGAACGACC
CCCAAACTCCTGACATATGAGAATAGTAAACACCCCTCTGGGATCCCTGATCGATTCTCTTGATCCAA
GTCTGGTAACTCCAGCTCCCTGACCATCACTGGGCTCCAGATTGAGGATGAGGCTGATTATTACTGC
>pVL107
CATTCTGTGCTGATTCAGCCATCCTCCCTCTCCACATCTCTGGGAGCAACAGCCAGACTCACCTGGACC
ATGAGCAGTGGTTTCAGTGTTGGCGACTACTGGGTACACTGGTTCCAGCAGAAGCCAGGGAGCCCTGC
TCAGTGTCTCCTGTACTACTAGTCAGACTCAGATAAGCACCAGGGCTCTGAGGTCCCCAGTCACTTCA
CTGGATCCAAAGATGCCTCGGCCAACGCAGGGCTTCTGCTCATCTCTGGACTCCAGCCTGAGGACGAG
GCTGACTATTACTGT
>pVL108
CATTCTGTGCTGATTCAGCCATCCTCCCTCTCCACATCTCTGGGAGCAACAGCCAGACTCACCTGGACC
ATGAGCAGTGGTTTCAGTGTTGGCGACTACTGGGTACACTGGTTCCAGCAGAAGCCAGGGAGCCCTGC
TCAGTGTCTCCTGTACTACTAGTCAGACTCAGATAAGCACCAGGGCTCTGAGGTCCCCAGTCACTTCA
CTGGATCCAAAGATGCCTCGGCCAACGCAGGGCTTCTGCTCATCTCTGGACTCCAGCCTGAGGACGAG
GCTGACTATTACTGT
>pVL109
GCTGTGATGACTCAGCTGCTGTCCCTCTCTGCATCTCCCAGAACATTAGACAGACTCACCTGCAACCTG
AGAAGTGGCATCAATGTTGGCAGCAAAGATACATTCTGGTACCAGCAGAAGCCAGGGACCCGTTACC
AGTGTCTCTCTACTCCACACAAATGCAGATAAGAACCAGGGCTTCAGGTCCCCAGCTGCTTCTCTGGA
TCCAAGGAGGCCTCAGACAATGCAGGGCTTCTGATCATCTCTGGACTCCAGCCTGGGGACGCAGCTGA
CTATGACTGT
>VL110
CAGTCTCTGACTCAGCCCGCCTCAGTGTCTGGGACCCTGGGCCAGACAGTCACCATCTCATGCTCTGG
AAGCAGCTCCAACATTGGGTATAGTAGTAGTTATGCAAGCTGGTACCAACAGATCCCTGGAACAGCCC
CCAAACTCCTCATCTATGCTACTAACAAACGAGCCTCAGGGGTCCCAGATCGATTCTCTGGCTCCAGG
TCTGGCAACACAGCCACCCTGACCATCTCTGGGGTCCAGGCTGACGACGAGGCCGATTATTACTGT
>pVL111
AGGTTCCCTCTCCCAGCCTGTGTCCTCCCTCTCCGTTCCCTCTAAACCTCTATGAGACTCACCTGCACC
ATGAGCAGGGGCTTCACTCTCGGTACCTTCTGGCTATTTTGGTACCAGCAGAAGCCAAGGAGTCCTCC
CAGGTGTTTACTTACATTCCACTCACATTCAGATGAGCACCAGGGCTCTGGGGGTCCCCAGGAAATTC
CCTGGATCCAATGATGCATCAGCCAATGCTAGGGTTTTGCTCATCTCTGCATTCCAG
>VL112
CAGCCTGTGCTGACCCAGCTGCCCTCCCTCTCTGCATCTCCTGGAACATCAGCCAGACTCACCTGCACC
CTGAGCAGTGACGTCAGTGTTGGCAGCTCTCTTATATTCTGGTACGAGCAGAAGCCAGGGAGCCCTCC
GGGGTATCTTCTGAGTTTCTACTCAGACTCAGTTAAGCACCAGGGCTCCGGGGTCCCCAGCCACTTCTC
TGGATCCAAAGACACCTCGGCCAATGCAGGGCTTCTGCTCATCTCTGGGCTCGAGGCTGAGGACGAGG
CTGACTATTACTGT
>pVL113
CAGTCTCTGACTCAGCCCGCCTCAGTGTCTGGGACCCTGGGCCAGACAGTCACCATCTCATGCTCTGG
AAGCAGCTCCAACATTGGGGTATAGTAGTAGTTATGCAAGCTGGTACCAACAGATCCCTGGAACAGCC

CCCCAAACTCCTCATCTATGCTACTAACAAACGAGCCTCAGGGGTCCCAGATCGATTCTCTGGCTCCA
GGTCTGGCAACACAGCCACCCTGACCATCTCTGGGGTCCAGGCTGACGACGAGGCCGATTATTACTGT
>pVL114
GTCCTCCCTCTCCGTTCCCCCTCTAAACCTCTATGAGACTCACCTGCACCATGAGCAGGGGCTTCACTC
TCGGTACCTTCTGGCTATTTTGGTACCAGCAGAAGCCAAGGAGTCCTCCCAGGTGTTTACTTACATTCC
ACTCACATTCAGATGAGCACCAGGGCTCTGGGGGTCCCCAGGAAATTCCCTGGATCCAATGATGCATC
AGCCAATGCTAGGGTTTTGCTCATCTCTGCATTCCA
>VL115
CAGCCTGTGCTGACCCAGCTGCCCTCCCTCTCTGCATCTCCTGGAACATCAGCCAGACTCACCTGCACC
CTGAGCAGTGACGTCAGTGTTGGCAGCTCTCTTATATTCTGGTACGAGCAGAAGCCAGGGAGCCCTCC
GGGGTATCTTCTGAGTTTCTACTCAGACTCAGTTAAGCACCAGGGCTCCGGGGTCCCCAGCCACTTCTC
TGGATCCAAAGACACCTCGGCCAATGCAGGGCTTCTGCTCATCTCTGGGCTCGAGGCTGAGGACGAGG
CTGACTATTACTGT
>VL116
CAACCTGTGGTGACTCAGCCGCCCTCCCTCTCTACATCTCCTGGAACATCAGGCAGACTCACCTGCACC
CTGAGCAGTGACATCAGTGTTGACAGCAAAGATACATTCTGGTACCAGCAGAAGCCGGGGACCCCTC
CCCAGTGTCTCCTCTACTACCACACAGACTCAGATAAGCACCAGGGCTCCAGGTCCCCAGCTGCTTCT
CTGGATCCAGAGATGCCTCGGCCAATGCAGGGCTTCTGCTCATCTCTGGGCAGCAGCCTGAGGACGAG
GCTGACTGTTACTGTG
>VL117
CAGTCTGTGACTCAGCCCGCCTCAGTGTCTGGGACCCTGGGCCAGACAGTCACCATCTCCTGCTCTGG
AAGCAGCAGCAACATTGGTGGTTATGGTGTTGGCTGGTTCCAACAGATCCAAGGAAAAGCCCCCAAA
ACCGTCATCTATGCTAATAACAAACGACCCTCAGGGGTCCCAGATCGATTTTCTGGCTCCAAGTCTGG
CAACACAGCCACCCTGACCATCTCTGGGCTCCAGGCTGAGGACGAGGGCGATTATTACTGT
>pVL118
CAGCCTTTGCTGACTCAATTGCCACCTGCAACTGCCTCCCTGGGAGCCTGGGCCGAGATTACCTACATC
CAGAGCAGGGAGCACAGCACCGGCAGGTCTGCGTCGCATCAGCAGCAGATGGGGAAGGCCCCTCAGT
TTGTGACATGGTTTAAAAATGACAGGAACCACAGCAAGGGGGATGGGATCCCCAGTCACTTCTCAGGT
TCTAGCTCTGGGGCTGACCACTAAGTAACCATCTCCGGTCTGAGGATGAGGCTGAGTCCCTCTGTGGA
ATAG
>pVL119
CAGCCTGTGCTGACTCAGCCACCCTCCCTTTCTGCATCTCCTGGATCATCGGGCAGACTCAACTGCACC
CTGAGCAGTGACGTCAGTCTTGGCAGCTATACCATACACTGGTACCAGCAGAAGCCAGGGAGCCCTCC
CCAGTGTCTCCTGAGTTTCTTCTCAGATTCATAAAAACATCAAGGCTTCGGGGTCCCCAGCCGCCGCTT
CTCTGGGTCCAAAGATTCCTCTGCCAACGCAGGGCTCCTGCTCATCTCTCTGGACTGCAGCCTGAGGAT
GAGGCTGACTCTTACTGTG
>pVL120
CAGTCTGTGCTGACTCAGCCACCATCACTCTCTGGGGCCCCAGGCCAGAGGGTCACCATGTACTGCAC
CGGAAGCAGCTCCAACATCGGGGGTGGCAATTATGTGTCCTGGTACCAACAGCTCCAGGGAACGACC
CCCCATACTCCTGACATATGAAGAACAGCAAACACCCCTCTGGGGTCGGGGTCCCTGATCGATTCTCT
TGATCTTGAGTCTGGCAACTCCGGATCCCTGACCATCACTGGGCTCCAGATTGGGGACGAGGCTGATT
ATTACTGC
>pVL121
CATTCTGTGCTGACTCAGCCATCCTCCCTCTCCACATCTCTGGGAACAACAGCCAGACTCACCTGCACC

ATGAGCAGTGGTTTCAGTGTTGGCGACTACTGGGTACACTGGTTCCAACAGAAGCCAGGGAGCCCTGC
TCAGTGTCTCCTGTACTACTAGTCAGACTCAGATATGCACCAGGGCTCTGAGGGTCCCCAGTCACTTCT
CTGGATCCAAAGATGCCTCGGCAAATGCAGGGCTTCTGCTCATCTCTGGACTCCAGCCTGAGGACGAG
GCTGACTATTACTGT
>pVL122
CATTCTGTGCTGACTCAGCCATCCTCCCTCTCCACATCTCTGGGAACAACAGCCAGACTCACCTGCACC
ATGAGCAGTGGTTTCAGTGTTGGCGACTACTGGGTACACTGGTTCCAACAGAAGCCAGGGAGCCCTGC
TCAGTGTCTCCTGTACTACTAGTCAGACTCAGATATGCACCAGGGCTCTGAGGTCCCCAGTCACTTCTC
TGGATCCAAAGATGCCTCGGCAAATGCAGGGCTTCTGCTCATCTCTGGACTCCAGCCTGAGGACGAGG
CTGACTATTACTGT
>pVL123
CAGTCTGTGCTGACTCAGCTGCTCTCCCTCTCTGCATCTCCCAGAACTTTAGGCAGACTCACCTGCAAC
CTGAGAAGTGGCATCAATGTTGGCAGCAAAGATGCATTGTGGTACCAGCAGAAGCCAGGGACCCGTT
GCCAGTGTCTCACCTACTCCACACAAATGCAGATAAGCACCAGGGCTTCAGGTCCCCAGCTGCTTCTC
TGGATCCAAGGAGGCCTCGGACAATGCAGGGCTTCTGATCATCTCTGGACTCCAGCCTGGGGATGCAC
CTGACTATGACTGTGC
>VL124
CAGTCTCTGACTCAGCCCGCCTCAGTGTCTGGGACCCTGGGCCAGACAGTCACCATCTCCTGCTCTGG
AAGCAGCTCCAACATCGGGAATAGTTATAGTTCTGTGGGCTGGTTCCAACAGATCCCAGGAACAGCCC
CCAAAACCCTCATCTATGCTACTAACACACGAGCCTCAGGGGTCCCAGATCGATTCTCTGGCTCCAAG
TCTGGCAACACAGCCACCCTGACCATCTCTGGGGTCCAGGCTGAGGACGAGGCTGACTATTACTGT
>VL125
CAGCCTGTGCTGACCCAGCCACCCTTCTTCTCTGCATCTCCTGGAGCATCAGCCAGACTCACCTGCACC
CTGAGCAGTGACATCAGTGTTGACAGCTCTCTCATATTCTGGTACCAGCAGAAGCCAGGGAGCCCTCC
CCTGTATCTCCTGAGTTTCTACTCCGACTCAGTTAAGCACCAGGGCTCCGGGGTCCCCAGCCACTTCTC
TGGATCCAAAGACACCTCGGCCAATGCAGGGCTTCTGCTCATCTCTGGGCTCAAGGCTGAGGACGAGG
CTGACTATTACTGT
>pVL126
ACCTTCTCCTGCACAGGCCGCAGCAGACATTGGGGGTCACCATGGCAACGGTACCAAGGACTCCCATG
CAGAACCCCTCTGTGTGTGACCTAGGACAGAGGTGATGGACCTGCCAGGAGTTTGAGCCTGATTCTCC
AGATTCAAGTTTGTTAACAAAGCCACTTTCACCACCTCTGCTCCAGCCTGAGGACTAATCTGATGACC
ATTGTT
>VL127
GAGGTTGTGCTGACTCAGCCTCGCTCTGTATCAGAGTCACTGGGACAGAAGGCCACCATCTCCTGTAC
CCACAGCAGCGACAACATTGGAAGCAGCTATGTGTACTGGTACCAGCAGCACCCGGGCAGTGCCCCC
ACCACCGTGATCTATGATGATGACGAAAGACCCTCTGGGGTACCCGATCGGTTCTCAGGGTCCATAGA
CAGCTCATCCAATGCTGCCTACCTGACCATCTCTGGGCTGCAGCCTGAGGACGAGGCCGACTACTACT
GT
>pVL128
CAGCCTGTGCTGACTCAGCTGCTCTCCCTCTCTGCATCTCCTGGAGCATCAGCCAGACTCACCTGCACC
CTGAGCAGTGGCATCAATATTGGCAGCAAAGATACAATGTGGCACCAGCAGAAGCCAGGGAGCCCTC
CCCAGTGACTCCTCTACTACCACACAGACTCAGATAAGCACCAAGGCTCCAGGTCCCCAGCTGCTTTT
CTGGATCCAAAGATCTTCAGCCAGTGCAGGGCTTCTGCTCATCTCTGGGCAGCAGCCCAAGGATGAGG
CTGACTGTTACTGTGA

>VL129
CAGTCTGTGACTCAGCCCGCCTCAGTGTCTGGGACCCTGGGCCAGACAGTCACCATCTCCTGCACTGG
AAGCAGCTCCAACATCGGGAGTGGTGATGTGTCCTGGTACCAACAGATCCCAGGAACAGCCCCCAAA
CTCCTCATCTATTCTTCCATTAGTAGGGCTTCCGGGGTCCCCGACCGATTCTCTGGCTCCAGGTCTGGC
AACACAGCCACCCTGACCATCTCTGGGCTCCAGGCTGAGGACGAGGCTGATTATTACTGT
>pVL130
CAGCCTTTGCTGACTCAATTGCCACCTGCAACTGCCTCCCTGGGAGCCTGGGCCGAGATCACCTACAT
CCAGAGCAGGGAGCACAGCACCGGCAGGTCTGCGTCGCATCAGCAGCAGATGGTGAAGGCCCCTCAG
TTTGTGACATGGTTTAAAAATGACAGGAACCACAGCAAGGGGGATGGGATCCCCAGTCACTTCTCAGG
TTCTAGCTCTGGGGTTGACCACTAAGTAACCATCTCCGGTCTGAGGATGAGGCTGAGTCCCTCTGTGG
AATAG
>pVL131
CAGCCTGTGCTGACTCAGCCACCCTCCCTTTCTTTCTGCATCTCCTGGATCATCGGGCAGACTCAACTG
CACCCTGAGCAGTGACGTCAGTCTTGGCAGCTATACCATACATACTGGTACCAGCAGAAGCCAGGGA
GCCCTCCCCAGTGTCTCCTGAGTTTCTTCTCAGATTCATAAAAACATCAAGGCTTCGGGGTCCCCAGCC
GCTTCTCTCTGGGTCCAAAGATTCCTCTGCCAACGCAGGGCTCCTGCTCATCTCTGGACTGCAGCCTGA
GGATGAGGCTGACTCTTACTGTG
>pVL132
CAGTCTGTGCTGACTCAGCCACCATCACTCTCTGGGGCCCCAGGCCAGAGGGTCACCATGTACTGCAC
CGGAAACAGCTTCAACATCGGGGGTGGCAGTTATGTGTCCTGGTACCAACAGCTCCAGGGAACGACC
CCCAAACTCCTGACATATGAGAACAGCAAACACCCCTCTGGGGTCACTGATCAATTCTCTTGATCTTA
GTCTGGCAACTTCGGATCACTGACCATCACTGGGCTCCAGATTGGGGACGAGGCTGATTATTACTGC
>VL133-ORF
CATTCTGTGCTGACTCAGCCATCCTCCCTCTCCACATCTCTGGGAACAACAGCCAGACTCACCTGCACC
ATGAGCAGTGGTTTCATGTTGGCGACTACTGGGTACACTGGTTCCAGCAGAAGCCAGGGAGCCCTGCT
CAGTGTCTCCTGTACTACTAGTCAGACTCAGATATTCACCAGGGCTCTGAGGTCCCCAGTCACTTCTCT
GGATCCAAAGATGCCTCGGCCAATGCAGGGCTTCTGCTCATCTCTGGACTCCAGCCTGAGGACGAGGC
TGACTATTACTGT
>pVL134
CAGCTGTGCTGACTCAGCTGCTCTCCCTCTCTGCATCTCCCAGAACATTAGGCAGACTCACCCGCAACC
TGAGAAATGGCATCAATGTTGGCAGTGAAGATACATTCTGGTACCAGCAGAAGCCAGGGACCCATTG
CCAGTGTCTCCTCTACTCCACACAAATTCAGATAAGCACCAGGGCTTCAGGTCCCCAGCTGCTTCTCTG
GATCCAAGGAGGCCTTGGACAACGCAGGGCTTCTGCTCATCTCTGGACTCCAGCCTGGGGATGCAGCT
GACTATGACTGTGC
>VL135
CAGTCTCTGACTCAGCCCGCCTCAGTGTCTGGGACCCTGGGCCAGACAGTCACCATCTCCTGCTCTGG
AAGCAGCTCCAACATCGGGAATAGTTATAGTTATGTGGGCTGGTACCAACAGATCCCAGGAACAGCC
CCCAAAACCCTCATCTATGCTAATAATAAACGACCCTCAGGGGTCCCAGATCGATTCTCTGGCTCCAA
GTCTGGCAACACAGCCACCCTGACCATCTCTGGGCTCCAGGCTGAGGACGAGGCCGATTATTACTGC
>pVL136
TCCTCCCTCTCTGTTCCCTCTAAACCTCTGTGAGACTCACCTGCACCATGAGCAGGGGCTTCACTCTTG
GTACCTTCTGGATATGTTGGTACCGGCAGAAGCAAAGGAGTCCTCCCAGGTGTTGTCTTACATTCTGCT
CACATTCAGACGAGCACCAGGGCTCTGGGGGTCCCCAGGCAATTCCATGGATCCAATGATGCATTAGC
CAATGCTAGGGTTTTGCTCATCTCTGGACTCCAGGTTAGGCTCACTAGGGGA

>VL137
CAGCCTGTGCTGACCCAGCTGCCCTCCCTCTCTGCATCTCCTGGGACATCAGCCAGACTCACCTGCGCC
CTGAGCAGTGATGTCAGTGTTAGCAGCTCTCTCATATTCTGGTACCAGCAGAAGCCAGGGAGCCCTCC
GGGGTATCTTCTGAGTTTCTACTCAGACTCAGTTAAGCACCAGGGCTCCGGGGTCCCCAGCCACTTCTC
TGGATCCCAAGACACCTTGGCTAATGCAGGGCTTCTGCTCATCTCTGGGCTCGAGGCTGAGGACGAGG
CTGACTGTTACTGT
>pVL138
GAGGCTGTGCTGACTCAGCCTTGCTCGGTGTCAGAGTCCCTGGGGCAGAAGGCCACCATCTCCTGCAC
CCGCAGCAGTGGCGAGATTGGAGACAAATATGAGCATTGGTGCCCACAGCACCCAGGCAGTGCACCC
ACCACCATGATGTACAAGGATGACCAAAGACCCCCCAGGGTTCTTGATCAGTTCTCTGGCCCCGTTGA
CAGCTCCTCCCATGCTGCCTGCCTGGCCATCTCTGGGCTGCAGACTGAGGATGAGGCCGACTAGGACT
AT
>pVL139
CAGCCTGTGCTGACTCAGCTGCTCTCCCTCTCTGCATCTCCTGGAGCATCAGCCAGACTCACCTGCACC
CTGAGAAGTGACGTCAGTTTTGGCAGCAAAGATACATTCTGGTACCAGCAGAAGCCAGGGAGCCCTC
CCCAGCATCTCCTCTACTACCACACACACTCAGATAAGCACCAGGGCTCCAGGTCCCCAGCTGCTTCT
CTGGATCCAAAGATGTCTCGGTCAACGCAGGGCTTCTGCTCATCTCTTGACTGCAGCCTGAGGACGAG
GCTGACTACTCCTGTG
>pVL140
CAGTCTGTGACTCAGCCCGCCTCAGTGTCTGGGACCCTGGGCCAGACAGTCACCATCTCCTGCACTGG
AAGCAGCTCCAACATCGGGAGTGGTTATGTGGGCTGGTACCAACAGATCCCAGGAACAGCCCCCAAA
ACTCCTCATCTATTATGCACTAATAGGGCTTCTGGGTCCCCGACCGATTCTCTGGCTCCAGGTCTGGCA
ACACAGCCACCCTGACCATCTCTGGGCTCCAGGCTGAGGACGAGGCTGATTATTACTGTG
>pVL141
CAGCCTGTGCTGACTCAGTTGTCACCTGCAACTGCCTCCCTGGGAGCCTGGGCTGAGCTCCCCTGCATC
CAGAGCAGTGAGCACAGCACTGACAATGCTGCATCGTGTCAGCAGCAGACAGGGAGGGCCCTCAGTT
TGTGACATGGCTTAATAGGGACAGGAGCCACAGCAAGCGGGATGCAATCCCCAGTCTCTTCTCAGGCT
CCAGCTCTGGGGCCGACCATTAAGTAACCATCTCCGGTCTGAGGATGAGGCTAACTCCATCTGTGGAA
TAGC
>VL142
CAGTCTGTGCTGACTCAGCTGCTCTCCATCACTGCATCTCCCAGAACATCAGGCAGATTCACCTGCAAC
CTGAGAAGTGGCATCAATGTTGGCAGCAAAGATACATTGTGGTACCAGCAGAAGCCAGGGACCCCTG
CCCAGTGTCTCCTCTACTACCACACAGACTCAGATAAGCACCAGAGCTCCAGGTCCCAGCTGCCTCTC
TGGATCCAAAGATGTCTCGGTCAATGCAGGGCTTCTGCATTGGACTCCAGCCTGATGACGAGGCTATT
ACTGT
>VL143
CAGTCTGTGACTCAGCCCGCCTCAGTGTCTGGGACCCTGGGCCAGACAGTCACCGTCTCCTGCTCTGG
AAGCAGCTCCAACATCGGGTATAGTTATAGTTATGTGGGCTGGTACCAACAGGTCCCAGGAACAGCCC
CCAAACTCCTCATCTATGGTAATAACAAACGAGCCTCAGGGGTCCCTGATCGATTCTCTGGCTCCAAG
TCTGGCAACACAGCCACCCTGACCATCTCTGGGCTCCAGGCTGAGGACGAGGCCGATTATTACTGT
>pVL144
TCCTCCCTCTCCGTTCCCTCTAAACCTCTGTGAGACTCACCTGCACCATGAGCAGGGGCTTCACTCTTG
GTACCTTCTGGCTATTTTGGTACCAGCAGAAGCCAAGGAGTCCTCCCAGGTGTTGTCTTACATTCCACT
CACATTCAGATGAGCACCAGGGCTCTGGGGGTCCCCAGGAAATTCCCTGGATCCAATGATGCATCAGC

CAATGCTAGGGTTTTGCTCATCTCTGCATTCCAG
>VL145
CAGCCTGTGCTGACCCAGCGGCCCTCCCTCTCTGCATCTCCTGGAACATCGGCCAGACTCACCTGCACC
CTGAGCAGTGATGTCAGTGTTGGCAGCTGTCTCATATTCTGGTACCAGCAGAAGCCAGGGAGCCCTCC
AGGGTATCTTCTGAGTTTCTACTCAGACTCAGTTAAGCACCAGGGCTCCGGGGTCCCCAGCATCTTCTC
TGGATCCAAAGACACCTCGGCCAAAGCAGGGCTTCTGCTCATCTCTGGGCTCGAGGCTGAGGACGAG
GCTGACTATTACTGT
>VL146-ORF
GTGCTGCCCCAGCCACCCTCAGTGTCAGAATTTCCAGGAGGAAGAATCTCCCTCAACAGGAAAACCAG
TAACATGGTGTGTAACTACGAGGGGGTCCTCTTATCAGGGTCAGCCAGGAAATGCCCTGGACACAGGC
TCTATGAGGAAGGCTTTGATGCTCAGGTATCCCAGGATGAGTCTCTGCTCCCTGTCAGCAGCTCAGCCT
CCCTGACCATCTCTG
>pVL147
CAGGCTGTGGTGACTCAGGAGGCCTCACTGACCATGTCCCCAGGAGGGACAGTCACTCTCACCTGTGG
CTGTAGCACTAAGTGGTCACCAATGGTCACCATCCATCCTGGTTCCAGCAGAAGAATGAAGAAATCCC
CACAACACTGATCTATGACACACACAACAAAAAACGCGCAGACCTCTGTGCAGTTCTCAGGCTCCATC
CCTGGGGCAAAGCTGCGCTGACCTCCTCAGGGGCCAGCCAGAGGAGGAGGCTGAGAGCCCCTGCAG
>VL148
CAGTCTCTGACTCAGCCCGCCTCAGTGTCTGGGACCCTAGGCCAGACAGTCACCATATCCTGCACTGG
AAGCAGCTCCAACATCGGGTATAGTTATAGTTCTGTGGGCTGGTACCAACAGATCCCAGGAACAGCCC
CCAAAACCCTCATCTATGCTACTAACAAACGACCCTCAGGGGTCCCAGATCGATTCTCTGGCTCCAAG
TCTGGCAACACAGCCACCCTGACCATCACTGGGCTCCAGGCTGAGGACGAGGCCGATTATTACTGT
>pVL149
CAGCCTGTGCTGACCCAGCCGCCCTCCCTCTCTGCATCTCCTGGAACATCAGGCAGACTCACCTGCACC
CTGAGCAGTGATGTCAGTGTTGGCAGCTCTCTCATATTCTGGTACCAGCAGAAGCCAGGGAGCCCCTC
CGGGGTATCTTCTGAGTTTCTACTCAGACTCAGTTAAGCACCAGGGCTCCCGGGTCCCCAGCCACTTCT
CTGGATCCAAAGACACCTCGGCCAATGCAGGGCTTCTGCTCATCTCTGGGCTCGAGGCTGAGGACGAG
GCTGACTATTACTGT
>VL150
CAGTCTCTGACTCAGCCCGCCTCAGTGTCTGGGACCCTGGGCTCGACAGTCACCATCACCTGCTCTGG
AAGCAGCTCCAACATAGGTGCTTATGTGGGCTGGTACCAACAGATCCCAGGAACAGCCCCCAAAACC
GTCATCTATGCTAATAACAAACGACCCTCAGGGGTCCCAGATCGATTCTCTGGCTCCAAGTCTGGCAA
CACAGCCACCCTGACCATCTCTGGGCTCCAGGCTGAGGACGAGGCCGATTATTACTGT
>pVL151
AGGTTCCCTCTCCCAGCCTGTGTCCTCCCTCTCTGTTCCCTCTAAACCTCTGTGAGACTCACCTGCACCA
TGAGCAGGGGCTTCACTCTTGGTACCTTCTGGATATGTTGGTACCGGCAGAAGCAAAGGAGTCCTCCC
AGGTGTTTTCTTACATTCTGCTCACATTCAGACGAGCACCAGGGCTCTGGGGGTCCCCAGGAAATTCC
CCGGATCCAATGATGCATCAGCCAATGCTAGGGTTTTCTCATCTCTGCACTCCAAGTTAGGGTCTCTAG
GACACATCATGGC
>VL152
CAGCCTGTGCTGACCCAGCCGCCCTCCCTCTCTGCATCTCCTGGAACATCAGCCAGACTCACCTGCACC
CTGAGCAGTGATGTCAGTGTTGGCAGCTCTCTCATATTCTGGTACCAGCAGAAGCCAGGGAGCCCTCC
GGGGTATCTTCTGAGTTTCTACTCAGACTCAGTTAAGCACCAGGGCTCCGGGGTCCCCAGCAGCTTCTC
TGGATCCAAAGAGACCTCGGCCAATGCAGGGCTTCTGCTCATCTCTGGGCTCGAGGCTGAGGACGAGG

CTGACTATTACTGT
>pVL153
CAGGCTGTGGTGACTCAGGAGGCCTCACTGACCATGTCCCCAGGAGGGACAGTCACTCTCACCTGTGG
CTGTAGCACTAAGTGGTCACCAATGGTCACCATCCATCCTGGTTCCAGCAGAAGAATGAAGAAATCCC
CACAACACTGATCTATGACACACACAACAAAAACGCGCAGACCTCTGTGCAGTTCTCAGGCTCCATCC
CTGGGGCAAAGCTGCGCTGACCTCCTCAGGGGCCAGCCAGAGGAGGAGGCTGAGAGCCCCTGCAGG
>pVL154
CAGTCAGTGCCGACTCAGCCCACCTCAGTGTCTGGGACTCTGGGCCAGAGGGTCACCACCTCCTGCCC
TGGAAGCAGCTCCAACATTGGTGGTGGTTATTATGTATCCTGGTACCAACAGCTCCCAGGAACAGCCC
CCAAAACTGTCATCTATTATGATAATAGTAGATCGGGTCCCTGATCGATTCTCTGGCTCCAAGCCTGGC
AACTCCATCTCCCTGACCATCACTGGGCTCCAGGCTGAGAACGAGGCTGCTTCTCACTGCCACTC
>VL155
CAGGCTGTGGTGATCCAGGAGCCATCATTCTCTGTGTCCCTAGGGGGGACGGTCATACTCACCTGTGG
CCTTAGCACTGGGTCAGTCTCTACCAGTAACTATCCTAGATGGTACCAGCAGACCCCAGGCAAAGGCT
CCCCGTACACTCACCTACAGCACAAACAACCTGCCTCTCTGGGATCCCTGAACGCTTCTCTGGATCCAT
CTCAGGAAACAAAGCCGCCCTCACCATCACGGGGGCCCAGCCCGAGGACGAGGCCGACTATTACTGT
>pVL156
CCGCCTGTGCTGACTCAGTGTCCTCCTTCTCTGCACCTCTGGGAACATCAAACAGACTCACCTGCACCC
TAAGCAGTGTTCTCAGTGGTGGTGGCTTCTGGATGAGCTGATAGCAGCAGAAACAGGGAGCCCCCTCC
AGTATCTCCTCAGTTTCAAGTCAGACTCAGGTAAGAATCAAGGTTCCAGGGTCCCCAGCCACTTCTCT
GGATCCAAAGTTGCCCCCACTGACTCAGACTTTTGGACGTCTCTGGGCTGCAGCCTGAAGAGGGTGAC
ATTACTGTG
>pVL157
TCCTACGAGCTGACGCAGCCAACCTCAGCATCAGTGGCTCTGGGACAGATGGCCAGGATCACCCGTGG
GGGAAATAACATTGGAAATAAATACGCTTACTGGTCCCAGCATAAGCCAGGCCAGGCCCCTATGCTA
GTCACCTATGACAGCAGCAAAAGGCCTTCAGGGATCCCTGACTGATTCTCAGCCTCCAACTCTAGGAA
CATGGTCACCCTGACCATCAGCAGGGCTGGAGCCGAGGACGAGGCTGACTATTACTGT
>VL158
TCCTATGAGCTGAGACAGACATCCACAGTGTCAGTGGCCCTGGGAGAGACAGCCCGGATCACCCATG
GGGGAAACAACATTGGAAGGAAAAATGCTTACTGGTACCAGCAGAAGCCAGACCAGGCCCCTGTGCT
GCTCATCCATAGAGACAGCAGCCGACCGTCTCGGATCCCTGACAGATTCTCAAGCTCCAACTCTGGGA
ACATGGCCACCCTGACCATCAGCGGGGCCCAGGCCGAGGATGAGGCTGACTATTACTGT
>VL159
TCTTCTGAGCTGACTCAGCCACCTACAGTGTCCGCGTCCTTGGGACAGACAACCAAGATCACCTGCCA
GGGAGGCAGCTTTGGAAGTTATTATGCTAGCTGGTACCAACAGAAGCCAGGCCAGGCTCCTGTGCTAG
TCATCTACAGCAACAACAAGCGGTCCTCAGGAATCCCTGACCAATTCTCTGGCTCCTACTCGGGGAAC
ACGGCCACCCTGACCATCAGTCGAGCCCAGACCGAGGACGAGGCTGACTATTACTGT
>pVL160
TCCTATGAGCTGACACAGCCACCCTCAGTGTCGTTGGTCCTGAGACAGATGGCCAGGATCACCTGTGG
GAGAAACAACATTGGAAGGAGAAGTACTCAGTGTTACCAGCAGAAGCCAGGCCAGGCCCCTGTGCTG
GCCACTTATGAGGATAGCGAGTAGGCCTCAGGGATCCCTGACTGGTTCTCTGGCTCCAACTCAAGGGA
CACAGCCACTCTGACAATTAGCAGGGCCCAGATCGAGGACAATGCTAACTATTACTGT
>pVL161
TCTTACGAGCTGAGGCCGCCACTCTCCGCATAGGTAGTCCTGGGAAAGAAACATCTGCTCTGGAATAA

CCTGGGGAATAAATAGGGATCTTGGTCCCAGTGGAAGCTGAACCCAGCCACTATGCTGGTGATTTGTG
AGGATAGCAAGAGGCCCTCAGGGATCTCTGACTGATTCTCAGACTTCCACTGCAGAGCACTGCCACCC
TGACCATCAATGGGATCCAGGCTTGGGATGATACTGACTATTACAGT

>pVL162

CTGACTCAGCTTCCCGAGGCATCTGGGGCCTCTGGGCAGATGGTCTCCACTTCCTGTGCTGAAGCAGC
AGTGACTTTGGGGTTTACAATTGTGTTTCCTGGTGCCAACATCTCCTGGCACAGCCCCACAGGACTCAG
GGTGGCCCAAGAATCAAACTTCACGAGACCCCAGATTCAAACTCTGGCAACACGACCTCTCTGAGCAT
CCTTCAGCTCCAGTTCAGCCATGAAGCTGATTATTATTGCT

>pVL163

TCCTCGGAGCTGACTCAGCCACCTTCAGTGTCTGTGACCCTAATACAGACAGTCAGCACCATCTGCCA
AGGAGAGATCCTAAAAACCTATTATGCTAACTGGTACCATCAGAAGCCAGGCCAGGTGTCTGTTCTCA
ACATCTATAAGGATAATGTGCGGCCCTCAGGGATCTCTGACTGATTCACTGGCTCCAAATCAGAGAAC
ACAGCCACCTTGACAATCGGTGGGACCCAGACAGAGGATGAGGCTGACTATTACTGT

>VL164

CCTCCTGTGCTGACCCAAGCCTCATCTGCATCTGCCTCCCTGGGAGCCTCAGTCAAGCTCACCTGCACC
CTGAGCAGTGCGCACAGCACCTACTATATTGAGTGGTATCAACAGACACCAGGGAAGGCCCCTGGAT
ATGTGATGAATCTTAAAAGTGACGGAAGCCACAGCAAGGGGGATGGGATACCTGACCGCTTCTCAGG
CTCCAGCGCTGGGGCTGACCGCTACTTGACCATCTCCAGTATCCAATCTGAGGATGAGGCTGAGTATT
ACTGT

>pVL165

TGTCCTGACTCAGTCTCCATAGGTATCTGGGGCTCCTGGACAGATGGTCTTCATTTCCTTTGCTGAAGT
AGTGGTGACATTGAGCGTTATGATTGTGTCTGCTGGTACCAACAACTCCTGACACAGCCCCACATGAC
TGAGGGTGGGGTCAAGAATCATATCTCACACACCCTAGACGCCAAGTCTGGCAACATGGCCTTTCTGA
GCATGTCTTGGCTCTAGCCCAGCCATGAGGCTGATTACTATTGCT

>VL166

TCTTCTATGCTGACTCAGCCACTTACCTTGTCCGTGGCCTTTGGAAGCACAGTCACTATCACATGCCAG
GGAGAGCTCCTAGACAGTTATTATGCTGAGTGGTACCAGCAGAAGCCAGACCAGGCTCCCATGCTGGT
CATATATTATGGAAGCAAACGTCCTTCGGGGATTTCTATCCGATTCTCTGGCTCCTACTCAAGCAAGAT
GGCCACCCTGACCCTCAGTGGGGCCTGGGCCGAGGATGAGGCTGACTATTACTGT

>pVL167

CAGTCTGTCCTGACTCAACCTCCCTAGGCATCTGTGCCCTCCTGGACAACGGTCACCAACTCCTGTGCT
GAAGCAGCAGTGACATTGGGGGTTATTATTGGGTCTCTAGGTATCAATATCTTCTGGTGCAGCCCAAA
CAACTGAGGGTGGAGTCAAGAATCAAACCTCAGTGACTCCAGACTCCAACTCTGGCAACACAGCCTCT
CTGAGCATCTCTCAGCTCCAGTCCAGCCATAATGCTGATTATTGT

>pVL168

TCTGAGCTGACTCAGCCCCCTGCCGTGTCTGTGGCCTTCAGGATAGATAGCCACCATCACCTGCCCGG
GAGAGAGAGCACAGAAAGCTATTACGCTTACTGCTCCCAGCAGAAGTTAGGCCAGGCCCCTGTGCTG
GTCATCTATGAGTCTAGTGAGAGGCCCTCGGGGATCTCTGATTGATTCTCTGGCTCCAACTTGGGGAA
CACGGCCACTCTCACCAACAGTTGGGCCCTGGCTAAGGACGAGCCTGATGATTACTAT

>VL169

TCCCATGACTTGACTCAACCACCCTCAACGTTGGTGGCCCTAGAACAGACGGCAACAATCCCCTGCTC
TGGAGATAATCTCGAGGATGAAAATGCTTACTGGTACCAGCAAAAGCCAGGCCAGTCCCCTGCCCTGG
TCATTTATAAGGATAGTGAGTACCCCTCAGGGATCCCTGACCAGTTCTCTGGCTCAAACTTGGGAAAC
ACAGCCACCCTGACCATCAGAGGGGCCAAGACAGAGGACGAGGCTGACTATTACTGC

>VL170

TCTTATGAGCTGACTCAGCTGTCCTCCATGTCGGTGGCCCTGGGACAGACACCCTGTATCACCTATGGG
GGAAACAACATTGGAAGGAAAAGTGCTTACTGGTACCAGCAGAAGCCAGAGTTGGTCATCTATAGTG
ATAGCAGCCAACCCTCTGGGATCCCTGACCGATTTTATGGCTCCAACTCAGGGAACACAGCCACCCTG
ACCATCAGCAGGGCCCAGGCCAAGGACAAAGCTGACTATTACTGT

>pVL171

CTGCCTGTGCTGACCCAGCCCCCATCTGCATCTGCCTCCCTCGGAGTCTCTGCCAAGCTCAACTTCACC
CTGAGCAGTGAGTGCAGCACCTACTCCAACCACCATATCAACAGAGTCCGTGGAAGGTCCCTGGGTAT
GGGATGAAGGCTAACAGTGATGGAAGCAACAGAAAGGGGGATGGGATCCCGAAATGCTTCTCAGGCT
CCACCTCTGGGGCTGACCTCTAATTAACCATCTCCACCATCCAGTCTGAGGACAAGGCTCAGTATCAC
TGT

>pVL172

CCATATCCTTTGCTGAAGCATCAGTGACTTTGTGGGTTATAATTGTGTCTCTTGGTACCAACAGCTCCT
GGCACAGTCAAAAAGAGTGAGAGCGGCTCAAGAATCAAACATCACAAACCTCAGACTCCAACTCTGG
CCACACGTCCTCTCTGAGCATCTATCAGCTCCAGTCCAGCCATGAGGCTGATTATTATTGA

>pVL173

GCCTCCTCTGATCTCACGGTAACCTGTGGTGTCTGTATCATTGGGACACACAGCCAAGATCATCTGCTA
GGGAGAGAACCTAGAAAGCTATCATGCTCTCTCGTACCAGCAAAAGCCAGATGAGGCCCCTATTTTGG
CCATCCATGGGTATAGTGAGCCGCCTCAGGGATCCCTGAATGATTCTCTGGCTCTAACTCAGGGAACA
TGGCCACTCTGACCATCAGTGGGGCCTGAGCTGAGGATGAGGCTAACTATCACTGT

>VL174

CTCCTACGTGTTGACGCAGCCAACATCAGTGTCAGTGACCCTGGGACAGACAGCCAGATTCACCTGTG
GGGGAAACAACATTGGAAACAAAGTTGTTCACTGTACCAGCAGAAGCCAGGACAAGCCCCTGTGCTG
GTGATTTATGATGATAACAGCCGGCCCTCAGGGATCCCTGACCGATTCTCTGGCTCCAACTCGGGGAA
CACAGCCACCCTGACCATCAGCAGGGTCCAGGCTGAGGATGAGGCTGACTATTACTGT

>pVL175

TCCTACGAGCTAACGCAGTCATGATCAGTGTTGTCGTCCCTAGAACAAACAGCCAGCATCACCTGTGG
GGGAAAAAATGCTCACTGGTACCAGCATAAGCCAGGCAAGGCCCCTGTGTTGGTCATCTTTGATGATAG
CAAACAGCCTTCAGGGATCCCTGACCATTTCTCTGGGGTCATTTCTCCAACACTGGGAACACAGCCAC
TCTGACCATCAGCAGGGCCCAGCATGAGGACTAGGCTGACTACTACTGT

>VL176-ORF

TCCTCTGAGCTGACACAGCCACCCTCAGTGTCTGTGGCCCCGGGACAGACAGCCATGATCACCTGCGG
GGGAAATAACATTGGAGGGAAATATTCTTACTGGTACCAGCTGAAGCCGGGCCAGGCCCCTGTGCTG
GTCATCTATAGTGATAGCAAACGACCCTCAGAGATCTCTTCCCGATTCTCTGGCTCCAACTCGGGGAA
CATGTCTGCCCTGATCATCAGCGGGGCCTGGACTGAGGACGAGGCTGACTATTACTGT

>VL177

TCCTCTGACCTGACTCAGCCAGACATGTGGTGTCTGTGTCATTGGGACAGACAGCCAGGATCACCAGC
CAGTGAGAGAGCACAGAAAGTTATTATGCTCACTGGTCCCAGCGGTGGTCAGGCCAGGACCCTGTGCT
GGTCATCTATGAGTATAGTGAGAGTCCCTCAGGGATCCCTGATTGATTCTCTGCCTCCAACTCAGGGA
ACTTGGCCACTCTCACCATCACTGGGGCCCTGGCTGAGGACAAGAATGATGATTACAGG

>VL178

TCTTCTGAGCTGACTCAGCCACCTGGGGTGTCTGTGGCCTTGGGACAGACGGCCACCATCAACTGCCA
GGGAGAGAGCATAGAAAGCTATAATGCTAACTGGTACCAGCAGAAGCCAGGCAAGGCCCCCATGCTG
GTAATCTATGCTAATAGTGAGCAGCCCTCAGGGATCCCTGAACGATTCTCTGGCTCCAGCTCAGAAGG

CTCAGCCACCTTGACCATCAGTGGAGCCCAGCCTGAGGACGAGGCAGACTATTACTGT

>pVL179

CTGCCTGTGCTGACACAGCCCCCATCTGCACCTGCCTCCCTGGGAGCCTCAGCCAAGCTCACCTGCAC
CCTGAGCAGTGAGCACAGCACTTACTCCATTCACTGTATCGACAGAGACCAGGGAAGGCCCCTGGGTA
TGTGATGAAGGTTAACAGTGATGGAAGCCACAGCAAGGGAGGTGGGATCCCCGACCTCTTCTTGGTCT
CTAGCTCTGGGGCTTACTGCTACGTAACTATCTCCAACATCCAGTCTGGGGGTGGTGCTGAGTATCACT
CTG

>pVL180

TCCTATGAGCTGACACACTAGCTCTCATTGTCGGTGGCCTTGGGGCAATCTGCCAGAATCACCGGTGG
GGGGAATAACCTTGGAAGTAAATATGCTCAGTGGTACCAGCAGAAGGCAGACCAGGCCCCTGTGCAG
GTCATCTATGGTAGCAGCAACCAGCCCTCAGGGATCCCTGACTGATATCTGGTTCCAACTTGGGGAAC
ACAGGCCACCTGACCAGCACCAGAGACCAGGCCGAGGACGAGGCTGACTATTACTGT

>pVL181

TCCTATGTGCTGACACAAACACCATCAGTGTCAGTAGCCCTGCAACAAACAGCCAGGATGACCTGTAG
GGGAAACAACATTGGAAGTAAAAATGTTTAGTGGTACCAGCAGAAGCCAGGCCAGGCCCCTGTGCTG
GTCATCTATAGAGATAGCCACCTACCCTCAGGGATCCATGACCGAATCTCTGGCTCCAACTCAGGGAA
CACGGCCACCCTGATCATCACTGGAGCCCAAGTCCAGGATGAGGCTGACTATTATTGT

>pVL182

CTGACTCAGCCTCCCAAGGCATCTGGGGCCTCTGGGGAGATAGTCTCCATCACCTGTGCTGAAGCAGC
AGTGACTTTTGGGGTTATAGTTGTGTCTCCTGGGACCAACAGTTCCTGGCAAAGCCCCACAGGACTGA
GCATTGGTCAAGAATCAAACTTCACAGACCCTAGACTCAAACTCTGGCAACATGACCTCTCTGAGCAT
CCTTCAGTTCCCATTCAGCCATGAGGCTGGTTTTTATTGCTG

>VL183

TCTTCTGAGCTGACTCAGCCATCTGCGGTGTCTGTGGCCTTGGGACAGACGGCCACCATCACCTGCAA
GGGAGGCGACTTTGAAAGTGCTTTTGGTAACTGGTACCAGCAGAAGCCAGGCCAGGCCCCTGTGCTGG
TCATCTATGCTAGTAATGAGCGGCCCTCAGGGATCCCTGAACGATTCTCTGGCTCCAGCTCAGGAGAC
ACAGCCACGCTGACCATCAGCGGGGCCCAGGCTGAGGACGAGGCCGACTATTACTGT

>pVL184

CTGCCTGTGCTGACCACACCCCCATCTACATCTGTCTCCCTGGGAGCCTCAGCCAAGCTCACCTGCACC
CTGAGCAGTGAGCACAGCACCTACCCCCTCCAGTGTATCTACAGAGACCAGGGAAGACCCCTGAGTAT
GTGATGAAGGTTAACATTGATGGAAGCAACAGCAAGGGGGACCGGATCCCTAGCTGCTTCTCAGGCT
CCACTTCTGGGGCTGACTGCTATTTAATCGCCGCCAAAATCCAATTTAGAGATTCTGGTGAGAGCCAT
GCAA

>VL185

TCTTCGGAGGTGACTCAGCCATCTGCGGTGTCTGTGGCCTTGGGACAAACAGCCACCCTCACCTGCCA
GGGAGGCTACTATGAAAGATATATTGTTAACTGGTACCAGCAGAAGCCAGGCCAGGCACCTGTGCTG
GTCATCTATGCAAATAGTGAGCGGCCCTCAGGCATCCCTGAACGAGTCTCTGGCTCCAGCGCATTAGA
CACATCCACGCTGACCATCAGTGGGGCCCAGGCTGAGGATGAGGCTGACTATTACTGT

>pVL186

CTGCTTGTGCTGACCCCGCCACCATCTGCATCTGCCTCCCTGGGAGCCTCAGCCAAGCTCACCTGCACC
CTGAGCAGTGAGCACAGTGCCTACTCTATTCACTGTATCAACAGAGACCAGGGATGGTCCCTGGGTAC
GTGATGATGGTTAGCAGTGACAGAACCCACAGCAAGGGGGACAGGATCCCTGACTTCTTCTCAGGCTC
CAGTTCTGGGGCTGACTGCTACTTAACCATCTCCAAAATCCAGTTGAAAGACAAGGCTGACTATCACT
CTG

>pVL187
CAGTCTGTCCTGACTCAGCCTCTCTAGGCATGTGTGGTCCCTTGGCAAATGGTCACCATACCCTGTGCT
GAAGCAGCAATGACATTTGCAGTTATCATGGTGTCTTTTTTTGCCAACAGCTCCTGGCGTGGCCCCACA
TGACTGACGATGGGTCATGAATCAAACTTCACAGACCCCAGACTCCAAATCTCGCAACAGGACCTCTC
TGAGTATCTCTTACCACCAGCCCAGCCATGAGACTGATTACTACTGT
>pVL188
TCCTCTGAGCTGACACAGCCACCCTCAGTGTCCCTGGCCCCAGGACAGAAGGCAAAGAGCATCTGTGG
GGGAAAGAATATTGGAAGTAAACGTACTTATTGGTAGCAGCAGAATCCAGGACAGGCTCCTGCAAGT
CATCTATTGTGATAGAATCAAGCCCTAAGGGATCCCCGGCCAGATCTCAGGCTCCAACCTTGGGAACA
CAGCCAACCTGACCATTAGAAGGGCCTGGGCCAAAGACAAGGCTGACTATTACTGT
>pVL189
CTCACGCAGCCTCCCTAGGCATCTGGGGCCTCTGGGCAGATGGTCACCATCTCCTGTGCTGAAGCAGC
AGCGACGTTGGGGGTTATAGTTGTGTCACCTGGGACCAACAGTTCCTGACATAGCCCCACATGATGGA
GCACTGATCAAGAATCAAACTTCACAGACCTTAGACTCAAACTCTGGCAACATGACCTCTTTGAGCAT
CCTTCAGTTCCCATTCAGCCATGAGGCTGATTTTTATTG
>VL190
TCTTCTGAGCTGACCCAGCCATCTGAGGTGTCCGTGGCCTTGGGACAGAGAGCCACCCTCACCTGCCA
GGGAAGCAACTTTGAATTTTTTTCTCCTAGCTGGTACCAGCAGAAGCCAGGCCAGGCCCCCGTACTGC
TCATCAATGTTAATAATGAGCGCCCCTCAGGGATCCCTGAACGATTCTCTGGCTCCAGCTCAGGAGAC
ACGTCCACACTGACCATCAGCGGGGCCCAGGCTGAGGACGAGGCTGACTATTACTGT
>pVL191
CTTCCTGTGCTGACCCCGCCACCATCTGCATCTACCTCCCTGGGAGCCTCAGCCAAGCTCACCTGCACC
CAGTGCAGTGAGCACAGCACCTACTCCATTCGCTGTATCTACAGAGACCAGCGAAGGCCCCTGGGTAC
GTGATGCTGGTTATCAGTGATAAAAACCACAGTGAGGGGGACAAGATCCCTGATTGGTTCTCAGGCTC
CAGCTCTGGGGCTGACTGCTACTTACCCATCTCCAAAATCCAGTTTAAAGACGAAGTTGAGTATCACT
CTG
>pVL192
CAGTCTGTTCCTACTCAGCCTCCCTAGGCATCTGGGGCCCAAGGACAGATGGTCATCACCTCCCGTGCT
GAAGCAGCAGTGATTTGGGGTTTATAACTGTGTCTCCTCATATCAACAGCTCCTGGCACAGCCCCGCA
TGACTGAGGAGTGTGGTCAACAATCAAACTTCACAGACCCCAGACTCCAAATCTGGAAAATCTCTGAG
CATCTCTCACCTTCAGCCCTGCCAAGAGTCTGATTATTATTTCT
>pVL193
TCTTCTAAGCTGACTCAGCCACCTTCAGTGTCTGCAGTCTTAGGACAGACTGCCACCATTACCTGTCAT
GGAGGAAACTTTGGAAGCAATTATGTGCACTGGTACCAGCAGAAACCAGGCCAGGCCCCTGTCACAG
TGATATATCAGGACAGCAAGTGGCCCTCAGGGATCCCTGACTGATTCTCTGCTCTAGCTCAGGGAATA
CGGTCACCCTGACCATCAGCAGGGCCCAGACAGAGGATGAGGCTGACTATTACTGCC
>pVL194
GCTCACTTGCACTGTGAGCAGTGAGCACAGCACCTACTTCATTCACTGTATCAACAGAGACCCGGGAA
GGGCCCTGTGTATGTGACGACGGTTAACAGCGATGAAGCCCCAACAGGTGGATGAGATCCCTGACCA
CTTATGGGGCTCCAGCTCTGGTGCTGACCGCTACTTACCCATATCCAACATCTAGTCCGAAGACAAGG
CTGAATATCACTGT
>pVL195
CAGTCTGTCCTGACTCGGTCTCCCTAAGGCATGTGGGCCCCTGGACAATGGTCACCATCTGATATGCTG
AAGCAGCAGTGAAAGTAAGGGTTACAATTTTTCTTTTCCTGGTAGCAACAGCTCCTGTCACAGCTCCA

CAAGACTGACTTCTGGGGTCACAAATCAAAGCTCACAGAACTCAGACTCTAACTTTGGCAACACAGCT
TCTCTGATCATCTTTCAATTCCAGTTCATCAATAAGGGTGATTATTATTGTTG
>VL196
TCCTTGGAGCTGACTCAGCCAGCTTCAGTGTCTGTGGCCTTAGGACAGACTGCCACGATCACCTGCCA
GGGAGGAATCTTTGACAAGAAGTATGTAGACTGGTACCAGCAGAAGCCTGGCGGGACCCCTGTGATA
GTGATTTATAAGGATAGTGAGCGGCCCTCCGGGATCCCTGGCAGATTCTCTAGCTCCAGCTCAGGGAA
CACAGCCACCCTGACCATCAGCAGGGCCCAGGCAGAGGACGAGGCCGTCTATTACTGC
>pVL197
GTACCTGTGCCGACCCAGCCCCCATCTGCATCTGCCTCCCTGGGAGACTTGGCCAAGCTCACCTGCAC
CCTAAGCAGAGAGTACAGAACACAGCATTGTATGGTATCAACAGCAATCAGGGAAGGCCCCTTGATG
TGTGATGTGGCTTAGGAGGGGCAGAAGCCACAGCAAGGGGGCATGATCCTTGACTGCTTCTCGGGCTC
CAACTCCGGTGCTGATTGCTACTTAACCATCTCCAACACCTGGTCTGAGGACAATATTAAGTGTTATTG
T
>pVL198
CCGCCTCTGAGGACCCAGGAGCCCTCTCTCAATGTGATGTCTGGTTTGGTAGTCACCCTCACCTAAACT
ACGAGTATGGGATCTGTCACAACTGGGAATGCATCAGGATAGTTCTTGAAGAAAACTGGCCAAGCTCC
TCTGCAGCTCATATACCAGACAAACAAATGCCTGACATCCATCTCCAAATGCTTTTCAAGTTCCATCCT
CAGGGGCAGAGCCACCCTCTCCATCACTGGGGCACAGCCCAAAGACGGGGTTGAGTATTTCTGT
>VL199
CAGTCTGCCCTGATTCAGCCTTCTTCGGTGTCCGTGGCTCTAGGACAGTCGGTCACCATCTCCTGTGCT
GGAAGCAGCAGTGACATTGGGTATTATAACTCTATTTCCTGGTACCAACAGCACCCAGGCACAACCCC
AAAGCTGCTGATTTACTATACCAATAAGAAGCACTCAGGGATCCCTGATCGCTTCTCTGGCTCCAAGT
CTGGGAACACGGCCTCCCTGACAATCTCTGGGCTCCAGGCTGAGGATGAGGCTGAGTATTACTGT
>pVL200
CAGTCTGCCCTGACTCAGCCTCCCTGTGCATTTGGGGCTCTGGGACAGTCAGTCACCATCTCCTGCACT
GGAAGCAGCAATGACATTGAGTGTTATAACCATGTCTCCTGGTACCAACATAGCTTGGACACAGTCCC
CAAACTTCTGATTTTTGCTATCAGTTCTTGGCCCTCAGGGATCCCTGTGCACTTCTCTGCCTCCAAGTCT
GGCAACGTGACTTCCCTGACCATCTCTGGGCTCTAGGCTGAAGAAGAGGCTGATTATTACTGT
>pVL201
CAGTCTGCCCTAACTCAGCCTCCATCAGTATCTGGCACTCCTGGAAATTGAGTCATCATCTGCTGTTCT
GGAAGCAGCAGTAATATTGGGGGTTATAATCTTGTGTCCTGGTACCAAAAGTGCCAGGGCATCGCCCC
CAAACTACTGATTTATGTTACCAGTGTTCGGCCCTCAGGGATCCCTGATAGCTTCTCTGGCTCCAAGTC
TGGCAACACAGCTTTGCTGACCATCTCTGGGCTCTACCCTGAAGACAAGGGTGATTATTACTGT
>VL202
CAGTCTGCCCTGACTCAGCCTGCGTCAGTGTCCGGGGCTCTAGGACAGTCGGTCACCATCACCTGTGC
TGGAAGCAGCAGTGACATTGGGGGTTATAATGCTGTCACCTGGTTGCAACAGCACCCGGGCACAGCCC
CCAAAGTTCTGATTTATAGTGTGAATACTCGGGCCTCAGGGATTCCTGATCGCTTCTCTGGCTCCAAGT
CTGGCAACACGGCCTCCCTGACCATCTCTGGGCTCCAGGTTGAGGACGAGGCTGATTATTACTGT
>pVL203
CAGCATGTTCTGACTCAGCCTCTCTCAGAACCTGACACTCCTGGAAATTCAGTCACCATCTCCTGCTCT
GGAAGCAGTAGCAATAATGAGGGTTATAACCTTGTGTCCTGATTCCAACAGCACCAGGGACAGCCCCC
AAACTCCTGATTTATGATGTAAATTCTCAGCCTTCAGGGATCTCTGATGGCTTCTTTGGCTCCACATCT
GGCAACACGGCCTCCCTGATCATGTCTGGGCTCCAACTTGAGGACAAGGCTGATCGTTACTGC
>pVL204

GAGTCTGCCCTGTCTCAGCCTCCAATAGCATCTGGTACTCCTGGATAATCAGTCACCATCTCCTATGCT
GGAAGCAGCAGTGACATTGGAGGTTATAATGCCATCTCCTGGTACCAACGGCTCCCAGGCACAGCCCC
CAAACTCCTGATTTATGATGTCAATACTAGGGCCTCAGGGACCTCTGATTGCTTCTTTGGCTCCATGTC
TGGGATCACGGCCTCCCTGACCATCTCTGGGCTCTAGGCTGAGGATGAGGCTGATTATTACTGT

>VL205

CAGTCTGCCCTGACTCAGCCTGCGTCAGTGTCCGGGACTCTGGGACAGTCGATCACCATCTCCTGTGCT
GGAAGCAGCAGCAACATTGGGAGTTCTAACTATGTCTCCTGGTTCCAACAGTACCTGGGAACAGCCCC
CAAACTCCTGATTTATAGTGTGAATACTCAGGCCTCAGGGATCCCTGCTCGAGTCTCTGGCTCCACGTC
TGGGAACACGGCCTCCCTGACCATCTCTGGGCTCCAGGCTGAGGATGAGGCTGATGATCACTGT

>pVL206

CAGCTGACCCTGACTCAGCCTCCCTCAGTATCTGACAATTCTGGAAATGTGGTCAGCATCTCCTATTCT
GGAATCAGCAGCAATATTGGGATTTATAACCTTATGTCCTGGTACCAACAGTGCCAGGAGTCAGTTCC
CAAATTCTTGATTTATTTCAGTTCTCATCCTTCAAGGACCACTAACCACTTCTCTAGCTCCAAATCTGG
CAACACGGCCTCCCTGACCACCTCTGGACTCTAACCTGAGGACGCTGATTATTGCTGT

>pVL207

CAGTCTGCCACGACTCAGCCTCCCTCAGTGTCCATGGCTCTGGGACAGTCGGTCACCATCTCCTGTGCT
GGAAACAGCAGTGATATTGGTGGTCATACTATTGTTCTCTGGCACCAAGAGTGCTCTGGAACAGCCCC
CAAACTCATGATTTAGGACATCAATACTCAGCCCTTAGGGATCCCTACCTACTTCTCTGGCTCCAAGTC
TGGTAACTCAGCCTCCGTGATCATCTCCAGGCTCCAACCAGTGGATGCTGATTATTACTGT

>pVL208

CAGCTTGTGGTGACCCAGGAGCCCTCCCTCACAGTGATATCTGGGCTGACAGTCACCCTCACCTGCTCT
GTGAATATGGGACCTGCCACAACGGGGGGTGCTCCAGGATTGTTCCTAAATAAAACTGGCTACGCTCC
TCTGCAGCTAATATAACAGACAAACAAATGCTTGACATCCATCTACAAGCACTTTTCGGGCTCCATCC
TTGGGGGCAGTGCTGCCCTCCCCATTGCTGGAGCACAGTCTAAGGAGGGGGCTGAGAACTTCTGT

>pVL209

CAGTCTGCCCTGACTCAGCCTCCCTCAGCATCTGGGGCTCTGGCACAGTTGGTCAACAACTCCTGTGCT
GGAAGCAGCAATGACATTGGGCATTATAATGAGGTCTCATGTTAGCAACAGCATCTGGGCTCAGTCCC
CAAACTCCTGATTTACTACACCAATAGGCGGCCCTCAGGGATCTCTGCTCACTTCTCTGCCTCCAAGAT
TGCGAACATGGCCTCCCTGACCATTTCTGGGCTCCAGGCCAAGGATGAGGCTGATTATTACTAT

>VL210

CAGTCTGCCCTGACTCAGCCTGCGTCAGTGTCCGGGACTCTGGGACAGTCGGTCACCATCTCCTGTGCT
GGAAGCAGCAGCAACATTGGAAGTTATAACTATGTTTCCTGGTACCAACAGCACCCGGGCACAGCCCC
CAAACTCCTCATTTATAGAGTCAGTTCTCGGGCCTCAGGGATCCCTGATCGCTTCTCTGGCTCCAAGTC
TGGGAACACGGCCTCTCTGACCATCTCGGGGCTCCAGGCTGAGGACGAGGCCGATTATTACTGT

>pVL211

CAGTCTGCCCTGACTCAGCCTCCCTCAGTGTTCATGGCTTTGAGAGTCGGTCACCATCTCCTGTGCTGG
AAGCAGCAGTGACATTGAACATCATATCTCAGTCTTCTGTTACCAACAGCACCTGGGCACAGACCCAA
GACTCCTGATTTATAGCGTCAGTACTCAGTCCTCAGGGATCTCTGATCCCTTCTCTGGCTCCAAGACTG
GAACACAGCCTCCTTCGCCAGCTCTGGGCCGCAGGCTGAGGACGAGATTGATTATTACTGT

>pVL212

CAGTCTGCCCTGACTCAGCCTCCTTCAGTGTCCAGGGCTTTGAGAGAAATGGTCACCTCTCGTGTGCTG
GAAACAGCAGTGATATTTGAGCTTATGACTGTGTACCAACAGCACCTGGGTGCAGCCCCAAACTACAG
ATTATTGAGGTCAACACTCGACCCTCAGAGATCCTGCTCACTTCTTTGGTTCCCAGTCTGACAACACGG
CCTCCCTGACCACCTCTGGTCTCCAGGCTGCGGATGAGGCTGATTATTACTGT

>VL213
CAGTCTGCCCTGACTCAGCCTGCGTCAGTGTCCGGGACTCTGGGACAGTCGGTCACCATCTCCTGTGCT
GGAAGCAGCAGCAACATTGGAAGTTATAACTATGTTTCCTGGTACCAACAGCACCCGGGCACAGCCCC
CAAACTCCTCATTTATAGAGTCAGTTCTCGGGCCTCAGGGATCCCTGATCGCTTCTCTGGCTCCAAGTC
TGGGAACACGGCCTCTCTGACCATCTCGGGGCTCCAGGCTGAGGACGAGGCCGATTATTTCTGT
>pVL214
CGGTCTGCCCTGACTCAGCCTCCCTCAGTGTTCATGACTTTGAGAGTCAGGTCACCATCTCCTGTGCTG
AAAGCAGCGGTGACATTGAACATCATACCTCAGTCTTCTGTTACCAACAGCACCTGGGCACAGCCCCC
AGACTCCTGATTTATAACGTCAGTACTCAGTCCTCAGGGATCTCTGATCCCTTCTCTGGCTCCAAGACT
GGAACACAGCCTCCTTGACCAGCTCTGGGCTGCAGGCTGAGGACGAGATTGATTATTACTGTT
>VL215
CAGTCTGCCCTGACTCAGCCTGCGCCAGTGTCCGGGACTCTGGGACAGTCGGTCACCATCTCCTGTGCT
GGAAGCACGGGATCTTATAAATACATTTCCTGGTACCAACAGCACCCGGGCACAGCCCCCAAACTCCT
AATTTATAATGGGAATAATCGGGCCTCAGGGATCCCTGATCGCTTCTCCGGCTCCACGTCTGGCAACA
CGATGTCCCTGACCATCTCTGGGCTCCAGGCTGAGGACGAGGCTGATTATTACTGT
>pVL216
CAGTCTGCTCTGACTTAATCTCCCTCAGCATCCAGAGCTCTGGGACAAAACTCTGGAAGCAGCAGTGG
CGCTGGGGGTTATAACGAGGTGTCCTGGTACCAATAAGGCCCAGGCACTGCCTTAGGTAAACTCCTGA
TCAATGTGTCAGTTCTCAGCCTTCTGGGATCTCAGACCATTCTTCTGGCTCTGGGTCCAGCAGCCTGGC
TTCTCTGACAATCTCCAAGCTCCAACCTGAGGACGAGGCAGATTGTCACTGT
>VL217-ORF
CAGTCTGCGCTGACCCTGCTTCCTTCAGTGTCCGGGACTTTGAGACAAGTTGTCACCATCTCCTGCACT
GGAAGCAGGAGCCACACTGGAACTTATAACCACGTCTCCTGGTACCAACAATGCCCGGGAAACTGCC
CCCACACTCCTGCTAGATGCTGTCAGTTCTCTGCTGTCAGGGCTTCCAGCCTGATTCTCAGGCTCCAGG
TCCGGCAGCACACCTCCTTGACCATCTCTGGACTCCACCCTGAGGGTGAGGCTGATGATCACTGT
>pVL218
CTGACACAACCAATCTCAGTGTCCCTGGTTCTGGGACAAAAGATCACAATTCCCTGCTCCGGAAACAG
CAGTGACACTTACAGTTATAATTACATGTCCTGACACCAACCACATTCCCAACGCATGAGGTGGTTGG
TCCTTGGGCCTCAGGTCCCTGCGCCATTCTCCAGCTCCAAGCCAGGCCACATGGGCTCCCTGACCTCCC
CCGGCTGTAGCTAGAGGACAAGCTGGGCACTATTGT
>pVL219
CAGCCTGTGGTGACTCAGAGCACTCACTATGTTCCCAGGAGGGACAGTCACTCTCACCTGTGCCTCCA
GCACTAGAGTGGTCACTAATGGTCACTATCCATCGTGGTTCCAGCAGAAACCTGGCCAAGTCCCCAGA
AGACTGACTTACAATACACACAACAAAAACTCATAGATCCCCACCCAGTTCTCAGGCTCCCTCCTTGG
GGACAAAGTTGCCCTCACCCTCTCGGGGACCTAGCCAAAGGATGACAAGGCTGAGAACTACTGT
>pVL220
ACTCAGGAGTGAGCCCCACACTCACTGTCCCCTTCCCCTGTTGAAGGCAGTCATCATTCTACCTGTTGC
TCTAGCACTGGAGAGGTCACCAATGGTCACTATCCTTGCTGGTGGCAGAAGAAATCTGTCCAAGCCTT
CAGTACATTGATTTATAACACAAACAGCAGACACTCATGGACCCCCTCCCAGTTCTCCGGCTCACTATT
TGAGGGCAAAGCTGCCCTGACCCTCTCAGGAGCCCAGTTGGAAGATAAGGCTGAGTGCTACTGC
>pVL221
CAGCCTGTGCTAACTCAGCTGCCCTCCCTTTCTGAAACTCTGGGAACATCAACCAGACTCACCTGCACC
CTGAGCAGTGGCTTCAGTGTTGGTAGCTATACCATATACTAGTTCCAGCAGAAGTCAGGGAGCCCTCC
CCAGTATCTCTTAAGATTTCATTCAGACTGAGACAAGCACCACAGCCCCAGGGTCTCCAGCCATTTCTC

TGGATTCAAAGATGCTTCAGCCAATGCAGGGCTTTTGCTCATCTCTGGGATCCATCCTGAAGACAAGG
CTGACTATTACTGT
>pVL222
CAGGCAGGGCTGACTCAGCCGCCCTCAGTGACCAACTTCCCAGGACAGACAGTCACAATCATTTGTAC
TGGAAACAGCAACAATGTTGGCAGTGAGAGGGCAGGGTGGCTGCAGCAGCTCCCGGGCCAGGTCTCC
AATATCCTGACCAGCAGGAATAAGAACAGGGCTTTCAGAGTCTCTGAGAGATTCTCTGACTCCAGGTC
AGGCAGCATGGCCTCCTGAGCATCTCTGGGCTCCAGCCTGAGGACGAGGCTGATTATCACTGCT
>VL223-ORF
CGGTCTTTGCTGACCCAGCCATCCTCGGTGTCAGGGACCCTGGACCAGGAAGTCACCATCTCCTGCTC
CGGGAACAGCAACATTGGATCCCATAATGTGTGTTGGTTCCAGCTGTCAGTGGTGCTACCCCAAAACT
CTGACTCTTGGAAGTACCAGGCCCTGAGAGATTCCAGATGATTCTCTGTCTCTAAGTCAGGCAACGTG
GTCTCTTTGACCACCTGTGGGCTCCAGCCTGAGGATCAGGTGATTGTTACTGT
>VL224
CAGACTGTGGTGGTCCAGGAGCCATCACTCTCAGTGTCTCCAGGAGGGAAAGTCACACTCACCTGTGG
CCTTAGCTCTGGGTCTGTCTCTTCCAGTAACTATCCTTTCTGGTATCAGCAAATCCTGGGCCAGGCTCC
CCGCACAGTTATCTACAACACAAAGAACCGCCCTTCTGGGATCCCTGATCGCTTCTCTGGGTCCATCTC
TGGGAACAAAGTCACTCTCACCATCACGGGTGCCCAGCCTGAGGACGAGGCCGACTATTCCTGT
>pVL225
CAGTGTGTGCTTGTGTGACACCTCCTGTGAAGCTCCTGATGGTCCCAGATCCAAGGTCACCTTCTCCTC
CACAGGGAGCAGCAGAGACATTGGGGGTCACCATGGCAATGGTACCAAGGACTCTCATGCAGAGCCC
CTACTTGTGTGACCTAGGACAGCAGTGGGGACCTTCCAGGGGTTCCAGACTGATTCTCGAGATCCACA
TTTATTACTAGAACCTCTTTGGCTACCTCTGGGCTCCAGCCTGAGGACGAGGCTGATTATCACTGT
>pVL226
CAGCCTGTGCTGACCCAGCCGCCCTCCCTCTCTGTATCTCCAGGAACAACAGCCAGACTCACCTGCAC
CCTGAGCAGGGGCGTCAGTGTGGCCAGCTATCCCAGTCTGTGCTGCTACTCAGACTCAAGCATGTGAT
TGGGATCAGCAGATCCCAGCTGCTTCTCTGGATCCAAAGATGCCTCGGCCAATGCAGGCTTTTTGCTTA
TCTCTGGGCTCCAGCCTGAGGACGAGGCTGACTCTTACCAT
>pVL227
GTGCTGACTCAGACCAGCTCTGTGTTGGAGTCTCCGGGGCAGGAGGCCACCATCTCCTGCACCCACAG
CAGTGGCTACATTGGGAGCAGATATGTGCACTGCTACCAGCAGTGCCCCCATCAGCGAAATCTACAAG
AGTGACAAAGGACCCTCCAGGGTTCCTGATCAGTTTTCTGACTCCATAGCCAGCTCCCCTAATGCCAC
CTGCCTGACCGTCTCTGAGCTGCAGCCTGAGGACGATGCCGACTACTCCGG
>pVL228
CAGTCTGGGTGACACAGGAAGCCTCAGTGTCTGGGTCTGTGGGACAGAAGGTCACCCTCTCCTGCGCT
GGAAATAGCAACAACGTTGGAATATTTGATGTGGGCTGGTACCAAATTTCTCACGGTGCCCCCAAAAC
TGTGATGCTCAGAAGTTCTTGGCCCTCAGGGATCCCAGCTCGGTTCTCTAGCTCCAAGTCCGGCAACA
CGGCCTATCTGACCATCTCTGGGCTCCAGCCTGAGGATGAGGCTGATTATTACTGT
>pVL229
CAGCCTGTGCTGACTCAGCTGCTCTCCCTCTCTGCATCTCCTGGAGCATCAGCCAGACTCACCTGCACC
CTGAGAAGTGACGTCAGTTTTGGCAGCAAAGATACATTCTGGTACCAGCAGAAGCCAGGGAGCCCTC
CCCAGCATCTCCTCTACTACCACACACACTCAGATAAGCACCAGGGCTCCAGGTCCCCAGCTGCTTCT
CTGGATCCAAAGATGTCTCGGTCAACGCAGGGCTTCTGCTCATCTCTTGACTGCAGCCTGAGGACGAG
GCTGACTACTCCACTC
>pVL230

CAGTCTCTGACTCAGCCCGCCTCAGTGTCTGGGACCCTGGGCCAGACAGTCACCATCTCCTGCTCTGG
AAGCAGCTCCAACATTGGGTATAGTAGTAGTTATGGGGGCTGGTACCAAACAGATCCCAGGAACAGC
CCTCAAACTCCTCATATATGAGGGTAACAAACGATCCTCAGGGGTCCCGAGATCGATTCTCTGGCTCC
AAGTCTGGCAACACAGCCACCCTCACCATCACTGGGCTCCAGGCTGAGGACGAGGCCGATTATTACTG
T
>pVL231
TCCTCCCTCTCTGTTCCCTCTAAACCTCTGTGAGACTCACCTGCACCATGAGCAGGGGCTTCACTCTTG
GTACCTTCTGGATATGTTGGTACCGGCAGAAGCCTAGGAGTCCTCCCAGGTGTTGTCTTACATTCCACT
CACATTCAAACAAGCACCAGGGCTCTGGGGGTCCCCAGGAAATTCCCCAGATCCAGTGATGCATCAGC
CAATGCTAGGGTTTTGCTCAACTCTGCACTCCAG
>VL232-ORF
CAGCCTGTGCTGACCCAGCCGCCCTCCCTCTCTGCATCTCCTGGAGCATCAGCCGGACTCACCTGCACC
CTGAGCAGTGACGTCAGTGTTGGCAGCTCTCTCATATTCTGGTACCAGCAGAAGCCAGGGAGCCCTGC
CCGATATCTCCTGAGTTTCTACTCAGACTCAGTTAAGCACCAAGGCTCCGGGGTCCCAGCCGCTTCTCT
GGATCCAAAGACACCTCGTCCAATGCAGGGCTTCTGCTCATCTCTGGGCTCGAGGCTGAGGACGAGGC
TGACTATTACTGTG
>pVL233
CAGGCAGGGCTGACTCAGCCACCCTCAGTGACCAAGTCCCCGGGACAGACCGCCACACTCACCTCCAC
TGGAAACAGCAGCAATGGTGGCAGTGAGGGGGCAATTTGCCTGCAGCAACCCCAGCGCCTTCTTCCCA
AACTGCTGATGAGGAGGAAGAAAAATCAGGCCTCTGGAACCTCTGAGAAATTCTCTGTCTCTGGGCCA
GGCAGTGTGGCCTCCTGAGCATCTCTGGTCTGGTGTGAATGTGGGGAAACTGAATCACTCGGA
>pVL234
CAGGCAGGGCTGACTCAGCCACCCTCAGTGACCAAGTCCCCGGGACAGACCGCCACACTCACCTCCAC
TGGAAACAGCAGCAATGGTGGCAGTGAGGGGCAATTTGCCTGCAGCAACCCCAGCGCCTTCTTCCCAA
ACTGCTGATGAGGAGGAAGAAAAAATCAGGCCTCCTGGAACCTCTGAGAAATTCTCTGTCTCTGGGCC
AGGCAGTGTGGCCTCCTGAGCATCTCTGGTCTGGTGTGAATGTGGGGAAACTGAATCACTCGG
>VL235-ORF
CAGCCTGTGCTGACCCAGCCGCCCTCCCTCTCTGCATCTCCTGGAGCATCAGCCGGACTCACCTGCACC
CTGAGCAGTGACGTCAGTGTTGGCAGCTCTCTCATATTCTGGTACCAGCAGAAGCCAGGGAGCCCTGC
CCGATATCTCCTGAGTTTCTACTCAGACTCAGTTAAGCACCAAGGCTCCGGGGTCCCAGCCGCTTCTCT
GGATCCAAAGACACCTCGTCCAATGCAGGGCTTCTGCTCATCTCTGGGCTCGAGGCTGAGGACGAGGC
TGACTATTACTGTG
>pVL236
TCCTCCCTCTCTGTTCCCTCTAAACCTCTGTGAGACTCACCTGCACCATGAGCAGGGGCTTCACTCTTG
GTACCTTCTGGATATGTTGGTACCGGCAGAAGCCTAGGAGTCCTCCCAGGTGTTGTCTTACATTCCACT
CACATTCAAACAAGCACCAGGGCTCTGGGGGTCCCCAGGCAATTCCATGGATCCAATGATGCATCAGC
CAATGCTAGGGTTTTGCTCAACTCTGCACTCCA
>VL237
CAGTCTCTGACTCAGCCCGCCTCAGTGTCTGGGACCCTGGGCCAGACAGTCACCATCTCCTGCTCTGG
AAGCAGCTCCAACATTGGGTATAGTAGTAGTTATGGGGGCTGGTACCAACAGATCCCAGGAACAGCC
CTCAAACTCCTCATATATGAGGGTAACAAACGATCCTCAGGGGTCCGAGATCGATTCTCTGGCTCCAA
GTCTGGCAACACAGCCACCCTCACCATCACTGGGCTCCAGGCTGAGGACGAGGCCGATTATTACTGT
>pVL238
CAGCCTGTGCTGACTCAGCTGCTCTCCCTCTCTGCATCTCCTGGAGCATCAGCCAGACTCACCTGCACC

CTGAGAAGTGACGTCAGTTTTGGCAGCAAAGATACATTCTGGTACCAGCAGAAGCCAGGGAGCCCTC
CCCCAGCATCTCCTCTACTACCACACACACTCAGATAAGCACCAGGGCTCCAGGTCCCCAGCTGCTTC
TCTGGATCCAAAGATGTCTCGGTCAACGCAGGGCTTCTGCTCATCTCTTGACTGCAGCCTGAGGACGA
GGCTGACTACTCCTGT

>pVL239
CAGCCTGAGCTGACTCAGCCTTCCTCCATTTCTGCATCCCCAGGATCTTAAGCTACACTCACCTGAACC
CTGAGCCGTGGCTTCAGTGTGGGCAGCTCTGCCATACACTGGTTCCAGCAGGAGACAGTGAACCCTCT
CTCGTTTCTCCTGGGATTTAGATCAGACTCAGATAAGCACCCGGGCTTGGGGATCCCCAGCCACTTCTC
TGGATCCAAAGGTACCTCGGCCAATGCAGGGCTGCTGCTCATCTCTGGGCTCCAGTGTGAGAATGAGG
CCAGTTATTACTGT

>pVL240
TCCTCCCTCTCTGTTCCCTCTAAACCTCTGTGAGACTCACCTGCACCATGAGCAGGGGCTTCACTCTTG
GTACCTTCTGGATATGCTGGTACCGGCAGAAGCCAAGGAGTCCTCCCAGGTGTTTTCGTACATTCCACT
CACATTCAGATGAGCACCAGGGCTCTGGGGGTCCCCAGGAAATTCCCCGGATCCAATGATGCATCAGC
CAATGCTAGGGTTTTGCTCATCTCTGCACTCCAG

>pVL241
CAGTCTCTGACTCAGCCCGCCTCAGTGTCTGGGACCCTGGGCCAGACAGTCACCATCTCCTGCTCTGG
AAGCAGCTCCAACATCGGGTATGGTAGTAGTACTGTGGGCTGGTTCCAACAGATCCCAGGAACAGCCC
CCAAAACCCTCATCTATGCTACTAACAAACGAGCCTCAGGGGTCCCAGATCGATTCTCTGGCTCCAAG
TCTGGCAACACAGCCACCCTGACCATCTGGGCTCCAGGCTGAGGACGAGGCCGATTATTACTGT

>pVL242
CAGCCTGTGCTGACTCAGCCGCCCTCCCTTTCTGCATCTCCTGGATCATCGGCCAGACTCAACTGCACC
CTGAGCAGTGACGTCAGTCTTGGCAGCTATACCATATACTGGTACCAGCAGAAGCCAGGGAGCCCTCC
CCAGTGTCTCCTGAGTTTCTTCTCAGATTCATAAAAACATCAAGGCTTCGGGGTCCCCAGCCGCTTCTC
TGGGTCCAAAGATTCCTCTGCCAATGCAGGGCTCCTGCTCATCTCTGGACTGCAGCCTGAGGATGAGG
CTGACTATTACTGTG

>pVL243
CCATTCTGTGCTGACTCAGCCATCCTCCCTCTCCACATCTCTGGGAACAACAGCCAGACTCACCTGCAC
CATGAGCAGTGGTTTCATGTTGGCGACTACTGGGTACACTGGTTCCAACAGAAGCCAGGGAGCCCTGC
TCAGTGTCTCCTGTACTACTAGTCAGACTCAGATATTCACCAGGGCTCTGAGGTCCCCAGTCACTTCTC
TGGATCCAAAGATGCCTCGGCCAATGCAGGGCTTCTGCTCATCTCTGGACTCCAGCCTGAGGACGAGG
CTGACTATTACTGT

>pVL244
CAGTCTGTGCTGACTCAGCCACCATCACTCTCTGGGGCCCCAGGCCAGAGGGTCACCATGTACTGCAC
CGGAAACAGCTTCAACATCGGGGGTGGCAGTTATGTGTCCTGGTACCAACAGCTCCAGGGAACGACC
CCCATACTCCTGACATATGAGAACAGCAAACACCCCTCTGGGGTCACTGATCAATTCTCTTGATCTTAG
TCTGGCAACTTCGGATCACTGACCATCACTGGGCTCCAGATTGGGGACGAGGCTGATTATTACTGC

>pVL245
CAGCCTGTGCTGACTCAGCCACCCTCCCTTTCTGCATCTCCTGGATCATCGGCCAGACTCAACTGCACC
CTGAGCAGTGACGTCAGTCTTGGCAGCTATACCATACACTGGTACCAGCAGAAGCCAGGGAGCCCTCC
CCAGTGTCTCCTGAGTTTCTTCTCAGATTCATAAAAACATCAAGGCTTCGGGGTCCCCAGCCGCTTCTC
TGGGTCCAAAGATTCCTCTGCCAACGCAGGGCTCCTGCTCATCTCTGGACTGCAGCCTGAGGATGAGG
CTGACTATTACTGTG

>pVL246

CAGCCTTTGCTGACTCAATTGCCACCTGCAACTGCCTCCCTGGGAGCCTGGGCCGAGATTACCTACATC
CAGAGCAGGGAGCACAGCACCGGCAGGTCTGCGTCGCATCAGCAGCAGATGGGGAAGGCCCCTCAGT
TTGTGACATGGTTTAAAAATGACAGGAACCACAGCAAGGGGGATGGGATCCCCAGTCACTTCTCAGGT
TCTAGCTCTGGGGCTGACCACTAAGTAACCATCTCCGGTCTGAGGATGAGGCTGAGTCCCTCTGTGGA
ATAG
>pVL247
CAGTCTGTGACTCAGCTCAGCCCGCCTCAGTGTCTGGGACCCTGGGCCAGACAGTCACCATCTCCTGC
TCTGGAAGCAGCAGCAACATTGGTGGTTATGGTGTTGGCTGGTTCCAACAGATCCAAGGAAAAGCCCC
CAAAACCGTCATCTATGCTAATAACAAACGACCCTCAGGGGTCCCAGATCGATTTTCTGGCTCCAAGT
CTGGCAACACAGCCACCCTGACCATCTCTGGGCTCCAGGCTGAGGACGAGGGCGATTATTACTGT
>VL248-ORF
ACTCAGCCGCCCTCCCTCTCTACATCTCCTGGAACATCAGGCAGACTCACCTGCACCCTGAGCAGTGA
CATCAGTGTTGACAGCAAAGATACATTCTGGTACCAGCAGAAGCCGGGGACCCCTCCCCAGTGTCTCC
TCTACTACCACACAGACTCAGATAAGCACCAGGGCTCCAGGTCCCCAGCTGCTTCTCTGGATCCAGAG
ATGCCTCGGCCAATGCAGGGCTTCTGCTCATCTCTGGGCAGCAGCCTGAGGACGAGGCTGACTGTTAC
TGTG
>VL249
CAGCCTGTGCTGACTCAGCCATCCTCCCTCTCTGCATCTCCTGGAGCATCAGCCAGACTCACCTGCACC
CTGAGCAGTGGCTTTGGTGTTGATGGCTACTGGATATACTGGTACCAGCAGAAGCCAGGGAACCCTCC
CCAGTATCTCCTGTATTACAAGTCAGATTCAGATAAGCACCAGGGGTCTGGGGTCCCCAGCTGCTTCT
CTGGATCCAAAGATGCCTCAGCCACTGCAGGGCTTCTGCTCATCTCTGGGATCCAGCCTGAGGACGAG
GCTGACCATTACTGT
>pVL250
CAGACTGTGCTGATGCAGCCTCCCACTGCATCCGCCTCCCTGGGAGCCTTGGCCAAGCTCACCTGCTCC
CTGAGCAGTGGCTCCAGTAACTGCAAAGTGGACTGGTACCAGCAAGGCCCAGGGAAGGGGCCAGGGT
TCCTGCTATGAATAGTCACCGGTGGTATTGTGGGATCCAAAGGGGATGGGATCCCTGGTCGCTTCTCA
GGCTCAGGCCTGGATCAGTACCTGCCCATCCAGAACATCCAGGTAGGGGACGAGGCTGACTCCTACTG
C
>pVL251
CAGTCTGTGGTGACCCAGGAGCCATCACTCTCAGTGTCTTCAGGAGGGACAGTCACACTCACCTGTGG
CCTTAACTCTGGGTCAGTCTCTTCCAGTAACCACCCCAGCTGGCACCAGCAAACCCCAGGCCAGGCTC
CCCGCACACTTATCTACTATACAAACACCCGTGCCTCTGGAGTCCCTAATCGCTTCTCTGGATCCATCT
CCGGGAACAGAGCCACCCTCACCATCACGGGGGCCCAGCCTGAGGACGAGGCCGACTATTACTGC
>VL252-ORF
CAGCCTGTGCTGACTCAGCCGTCCTCCCTCTCTGCATCTCTGGGAACAACGGCCAGACTCACCTGAAC
CCTGAGCAGTGGCATCGCTGTTGGCAATTGTCCCATACACTGGTGTCAACAGAAGCCAGGGAGCCCCC
CTCGTATCGCCTGTACTACTACCCAGACTCAGATAAGGACCAGGGCTCCAGGGTTCTGAGCCGCTTCT
CTGGATCCAAAGATGCCACGGCCAATGCAGGGTTTTTGCTCACCTCTGGGCTCCAGCCTGAGGATGAG
GCTATTACCA
>VL253-ORF
CAGGTGGTGCGGACTCAGCCACCCTTAGTGACCACATCCCTGGGACAGATGGCCATACTCACCTGCAC
CAGAAACAGCAACAGTGTTGGCAACAAGGAAGTAGCATGGCTGCATGGCTGCAGCAACACCCAGGCC
ATGTCCTCAACCTTCTGCGATTCCACAGGGATAACAACTGACCCTTTGGAGTCTCATAGAAATTCTCTG
GCTCCAGGTTGGCAACTTGGCCTCCAAAATATCACTGGGCTCCATCCTGGGGGTAAGACTGATTATTA

CTCG

>pVL254

GTGCTGACTCAGTTCCACTCACTTGGAGTCTCCAGGGCAGAAGGTCACCATCTCCTGCACCTGCAGCA

GCGGCAACATTGATGACTATTATGCCTACTAGTACCAGCACCACCCAGGCAGTTCCCCCACCACTGTG

GTCTACAAGGATGACCAAAGACCCTCTGGGGTTCCCGATTGGTTCTCTAGCTCTGTCAACATCTCCTCC

AACACCGCCTACCTCACCACCTCTGGGCTGCAGCCTGAGGATGAGACCGACTATTACTC

Vκ gene sequence

>pVκ1

GACACTGTGTGGACCCCGTCTCCAGCCTCCCTCCCTGTGTCCCAGGAGAGAAGGTCACCGTCACCTGC
TGGGCCAGCCAGAATATTAACAGCATCCTAGCCTGAAATCAGCAAAAACCAGCTCTCAGGCTCCTGCT
CACTCATGCTGCCCCCTGGGCATGATGCCTGCCTGGTCAGGGGCAGTGGGTCCGGACAGACATCACTC
TCACCATCAGGAGCCCAACTGCCTTCAGGAGCACAGACACCCTCCTCCTGT

>pVκ2

GACATCATCTGACCCAGACACCAACCTCTCTGTCTGAGTCTCCAGGAGAGAGTGTCACCATTTCCTGTC
AAGCCAGCGCTGATGTACATGGAAAAACATCTTGGATCCGGTTGAAACCTGGACAAAGATTTGCACCC
TTTATATCTTACATCTCTATCTTGGCCCTGGGCATCCCAGCCTGGCTCAGCAGCAGGGAAAGTGGAAC
CACTTGACCTGAGCTATTGCTGT

>Vκ3

CAGATCATCCTGACTCAGTCTCCAGAGACCCTGGCAGCATCTCAGGGCAGCTTTATCTCCATCACCTGC
AGAAGCAGCATAGAGGTAGGTACCTCCATGGCCTGGTACCAACAGAAACCCAATGAAGCTCCCAGGC
TCCTCATCTTTGGGGCTTCTGCCCGGGCCGTGGGAACCCCATCCAGGTTCCGAGGAAGTGGTTCTGGCT
CTGTCTTCTCCCTCACCATCCATGGACTGGAAGCTAAAGACTTTGGGGTGTATTACTGC

>pVκ4

GGAGCACTATGCTGACCAGTCTCCATCCTCATCTCTGTGTCTCTAGGAGAGAGCGTCTCCATCATTTGC
AGGGAAGATCAGAGCATTAGCGATTACTGAACCTGGTATCAGCAGAAACCAGGCCAGGTTCCTAAGC
TTCTCATCGATGATGCTGATGATCGATATTTTAGTGTCCCAGCCCAGTTCACTGGGATACAGTCTGAGA
CAGAATTCATTCTCCAAATCAGCAAGGTAGGGGCTGATGATGTTGCCAGTTATCACTGC

>Vκ5

GAAGGCTTGTTGACACAGTCTCTGGCCTCCCTATCTCTGTCTCCTAGAGAAAGAGCCACCCTCACCTGC
AGGGCTAGTGAGAGTGTCAGTAGCTATTTAGCCTGGTACCAGCAGAAACCTGGGAAGGCTCTCAAGCT
CCTCATGTATGGTGCATCCACCAGTGCCACTGGCATCCCAGCCAGGTTCAGTAGCCATGGGTCTGGGA
CAGACTTTACTCTCATCATCATCCACCTGGATCCTAAAAATGTTGCAGTT

>pVκ6

ACGGGGACTAGTGTTGACCTAATTTGCAACTTCCGAACAAAACTTCAGGAGAGAGAGTTACCATCAGC
TACAAGGCACCAAAATAATAGTAGCTACTTGAACAGGTACCAATGAAAAACAGGACAGGCTCCCATT
CTTCAGCATTATCATACCTCTGACCTCTACCCTGGTGTCCTCACTTGGTTTCTTACTGTGGGTCTGAGA
CAGAATTCACCTCTACAATCTGCAGCCTGGAGGCTGAAAACGAAGCATATCATTACTCT

>pVκ7

GCTGCTGTGCTGACTCAGTCTCCATCCCTCTTGTCTGTGGCAGTGGGAAGGAGTGTCTCCATCTCCTGC
CAGGCCAGTCTCAGTCTCATCCACAGGAATGGACACACCTACCTGAACTGGTGCCAGCAGAAACCAC
GTCATCTCCTGGACCACTGATCCATAGTGTGTCCAACAGGGCTCCTGGGTTCCCAGCTGGTTCAGTGGC
AGTGGGTCAGGGATGGGTTTCACACTCCAAAACACCGGTGTAGAGATTGGAGACACTGTACATACTTC
TGTGCA

>Vκ8

GATATTGTAATGACCCAGACTCCACTCTCTTTGCCTGTCGTCCCTGGAGAGCTGACCTCTGTCTCCTGC
AGGTCTAGTCAGAGACTTCTATATAGTAGTGGAAACACCTTTTTGAATTGGTACCTGCAGAAGCCAGG
CCAGTCTCCACAGCTCCTTATCTATATGCTATCCAACTGGCTCTCTGGGGTCCTAAACAGATTCAGTGG
CAGCGGATCAGGAACAGATCTCACCCTTAAAATCAGCAGAGTGGAGGCTGAGGATGTTGGAGTTTATT
ACTGC

>Vκ9

GACACAGTTTTGACCCAGACCCCACTCTCTCTGTCTGTCATCCCTGGAGAGTCAGCCTCCATCTCTTGC
AAGTCTAGTCAGAGCCTCCTACATGGTAATGGAAACACCTATTTGCATTGGTACCTGCAGAAGCCAGG
CCAGTCTCTTCAGTGCCTGATCTCTATGGTTTCCAATTGGGCATCTGGGGTCCCAGACAGGTTCAGTGG
CAGCGGGTCTGGGACAGATTTCACCCTTAAAATCAGCAGGTTGGAAGCTGAGGATGTTGGAGTTTATT
ACTGC

>pVκ10

GACATTGTGTGGATACCATCTCCAATCTCCTTCCCTGTGTCCCAGGAGAGAGCGTCTCCGTCACCTGCA
GGGCCAGCCAGAGTATTAGCATTATCCTAGTCTGAAATCAGGAAAAACCAGGACCAGCTCCCAGGTTC
CTACTCACTCATGCGGCCCCTGAACATGACGCCTGCCTGGTCGGGGCAGTGGGTCTGGACAGACTTCA
CTCTCACATCATCAGTTGAGCTGCCTTCAGGAGCACAGACACCTTCC

>pVκ11

CAGACATCAGCCTGCCTGTCTGTATCTTGAGGAGAGAGAGTCACCATTTCCTGCCAAGCCAGTGCTGA
TGTACATGGAGAAATAGCTTGGATTTGCATAAAACGTGGAGAAAGATTTGAACTCCTATATATCTCAG
GTCTCTACCTTGGCCCCACAAGTCCCAACCCGTTTCAGCAGCAGGGGATGTGGAACCAGCTATGCCTT
CAGCATTAGCGCCCTGGAGCC

>Vκ12

GATGTTGTGTTGACCCAGACTCCACTCTCCCTGCCTGTCGTCCCTGGAGAGCCGGCCTCCATCTCCTGC
AAGTCTAGTCAGAGCCTCAAACATAGTGATGGGAAAACCTATTTGTATTGGTTCCTACAGAAGCCAGG
CCAGTCTCCAAAGCTCCTGATCTATTTGGTTTCCACCCGGTACTCTGGGGTCTCAGACAGGTTCAGTGG
CAGCGGATCAGAAACAGATTTCACCCTGAAAATCAGCAGAGTGGAGCCTGAGGATGTTGGAGTCTAT
TACTGT

>Vκ13

GATGTTGTGATGACCCAGACTCCACTCTCCCTGTCTGTCGTCCCTGGAGAGCCGGCCTCCATCTCCTGC
AAGTCTAGTCAGAGCCTCAAACATAGTGATGGAAATACCTATTTGTATTGGTTCCTACAGAAGCCAGG
CCAGTCTCCAAAGCACCTGATCTATTTAGTTTCCATCCGGGTCTCTGGAGTCTCAGATAGGTTCAGTGG
CAGCGGATCAGAAACAGATTTCACCCTGAAAATCAGCAGAGTGGAGCCTGAGGACGTTGGAGTCTAT
TACTGT

>pVκ14

ATGAACCATTATAATCCCAATAAAATTACTTAAAAAAAATTTGCTACTTCCAGAGAAAGCTTCAGCAG
AGAGAGTCACCAGCGCGGCAAGTCAAGACATTAGTAGCTCCTTGAACTGGTACCAACAGAAAACAAC
AGACTCTCAAGCTTCTCATTCACATTTCCACCCTGGGCCTCTGCATGGGTTAGCAATAGTGGGTATAGG
ATATATTTCACCCTCACTCTCAGCACCCTGGTGGCTGAAGATGCAGCTTATTATTACTGT

>Vκ15-ORF

GAAATAACAGTCACACAGTCTCTGGAATCCATGTTAGTGATTCCAGGAGACAAAGTCATCATCACCTG
CAAAGCCAGCCAAGACATTGGTGATGATGTGAACTGGTATCAACGGAAACCAGGAGAAGCTCCTAAG
CTCATTATTAAAGAAGCTACTACTCTCTGGTCTGGGGTTCCCTCTCGGTTCAGTGGCACTGTGCATGGA
GTAGATTTTACCCTGACAATTGATGACGTAAAATCTGAGGATGCTGCATATTATTTCTGT

>pVκ16

GAAATCATCTTGACCCAGTCTCTGGCTTCCATAGCTGTTTCCCAAGAGAACAGAGTCACCATCACCTG
CAGGATCAGCACAAGCATAAGTACTGGCTACTTAACACTGGTACCAACAGAAGCCAGGAGCCCGTCC
AAATCTCCCTGTTTACAGGACGTCAAGCCTGGCTTCTGGGATTCCATCCCCCTTCAGTGGCAGCAGATC
TGGGATGTCTTATTCTCTCACAATCAGCAGTGTGAAGGCTGAAGACGCTGCAATCTACTACTGT

>pVκ17

GATATTGTGATGACCCAGACTCCACTTTTCTTGCCCGTCATCCCTGGAGAGCTGGCCTCCATCTCTCCT

GTAGGTCTAGTTAGAGCCTCCTACATGGTAATGGAAACAGGCGTTTGAATTGGTACCTTCAGAAACCA
GGCCAGTCTCCATGACTCCTATTCCATACAGTATCCAACTGGGCCTCTGGGATCCCAGATAGGTTCACT
GGCAGTGGATCAGGGACAGATTTCATGATTAAAATCATCAAGGTGGAGGCTGAAGATGTTGGAGTTT
ATTACTGC

>pVκ18

TATATTGTGATGATCCAGAGCCTGCTCTCTCTGACCATCATCCCTGGAGAACAGGCCTCCATCTCCTGG
AGGTCTAGTCAGAGCCTTCTATATAATAATGGAAACACCTATTTACATTGGAAACACCTATTTACATG
AAGGCTGGCCAGTCTCCATGGCTCCTGATCTATGGGATTTCCAAGTGTTACTCGAGTGCCAGACAGGT
TCAGTGGCAGTGGGTCAGGGACAGATTTTACACTTAAAATCAGCAAAGTGGAGACTAAGGACGTTGG
GGTTTAT

>pVκ19

GATATTGTGATGATGCAGACTCTACTCTCTCTACTTGTCACCTCTGGAGAGCCAGCCTCTATCTCCTGC
AGATCTAGTCAGAGCCTAGTGATGGATACATCTATGTGAATTGGTACTTTCAGAAGCCAAGCAAGTCT
CCATAGCTCCGTATCTTTGTACTTTCCCACTGCCTCCCTGGGGTCCCAGACAGGTTCAGGGGCAGCAGG
CCAGGGACAGATTTCATTCTCAAAGGCAGCAGAGTGGTGGCTGGGGATTTTGGAGTTTATTACTGC

>Vκ20

GATGTTGTGATGACCCAGACTCCACTCTCCCTGCCTGTCGTCCCTGGAGAGCCGGCCTCCATCTCCTGC
AAGTCTAGTCAGAGCCTGCTGGATAGTGATGGAAAAACCTATTTGTATTGGTACCTGCAGAAGCCGGG
CCAGTCTCCAAAGTTCCTGATCTATTCAGTTTCCAACCGGGACTCTGGGATCTCAGACAGGTTCAGTGG
CAGCGGGTCAGGAACAGATTTCACCCTGAAAATCAGCAGAATGGAGCCTGAGGATGTTGGAGTTTATT
ACTGT

>Vκ21

GAGATTGTGCTGACCCAGTCTCCAGCCTTCCTGTCTGGGACTCCAGGGGAGAAAGCCACCATCACCTG
CCGGGCCAATCAGGGCATTAGCAACTACTTACACTGGTACCAGCAGAAGCCAAATCAACCTCCAAAG
CCCATTATAAAGTATGTTTCCGAGTCCATTTCGGGGGTCCCAGCACGATTCAGTGGCAGTCGATCTGG
GACAGATTTCACACTCACCATCAGTAGGCCAGAAGCTGAAGATGCTGCAACTTATTACTGT

>pVκ22

GACATGGTGTGGATCCCCTCTTCAAGCTCCTTTCCTGTGTCCCAGGAGAGAGGGTCACCATAACCTGC
AGATTTGTAGGGCCAGCCAGAGTATTAACATCATCCTGCCTGAAATCAGTAGAAATGAGGACCAGCTC
CCAAGCTGCTACTCACTCACACTGCCGCCTGGATGTGATGTCTGCCCGGTGGGGGAAGTGGGTCTAGG
TAGGTGTCACTCTCACCACCATCA

>pVκ23

TAGATCATGCTGACTTAGTCTCCAGAGACCCTGGCAGCATCTCAGGGAAGCTTTGTCTCCATCACCTGT
AGAAGCAGAATGGAGGTAGGTACCTCCGTGGCCTGGTACCAACAGAAACCCAACGAAGCTCCCAGGG
TGCTCATCTTTGAGGCAGCGGCTCTGGCTCTGTCTTCTCCCTCGCCATCCATGGACTGGAAACTAAATA
CTTTGGGGTGTGTTACTGCCAGCAGCACTTGAGCTGGCCTCTCACAGTGACAGAGCCCC

>Vκ24

GAGATCCAGATGACCCAGTCTCCAGCCTCCCTGTCTGCATCTCTAGGAGACAGAGTCACCATCACTTG
CCAGGCCACTCAGGGCTTTAACACTTGGTTAGCCTGGTATCAGCAGAAAACAGGGAAGGCTCTTAAGT
TCCTGATCAGTAAGGCAACCATTTTGCACACTGGGGTCTCTTCGAGGTTCAGTGGCAGTGGAACTTGG
ACAGATTTCACTCTCACCATCAGCAGCCTGGAGCCTGAAGATGCTGCAACTTATTACTGT

>pVκ25

AGAAGGAGTCACCGTAACCAGCCAGGACAGTTAAGTCTTAGCAGTGACTTAGCTTGGTATTGGCAGG
ACTCAGTTTAGGATCCTATGATCCTCATCATTAATGCATCCATCTGATCTTTAGGACTTTACCTTGATTC

AGTAGAATTGGTCTAGGACAGGTGTCTCACTCATAGCTAAGTGCCTGCAGGTTGAGGAT

>pVκ26

CAAGTTGTGATGACACAGTCTCCAGCCTCCTTCTATTTGTCTCCAGGAGAAAGAGCCACCCCTCACCTG
CAGGGCCAGTCAGAGTGTTAGCAGCAACTTATCCTGGTACCAGAAGAAACCTGGGTAGCCTCCCAGG
CTCCTCATCTATGGTGTGTCCTCCAGGGCCACTGCCATCCCAACCCGGTTCAGTGGGAGTGGGTCTGG
AACAAACTTTACTCTCACTATTAGCAACCTAGAGCCTGAAGATTCTGCAGTT

>Vκ27-ORF

ACTGCTGTGCTGACTCCATCTCCACTGCTCTTCCCACTGTCCGCGGCACTGGGTGAGAGTGCCTCTGTC
TCCTGTAAGGCCAGTCTCAGTCTCATCTACGGTAATGGACACATTTACTGGAACTATTTCCAGCAGAA
ACCACATCAATCTCCTCAACCACTGATCTATGGGGTGTCCAACAGGGCTCCTGGGGTCCCAGCTTGGT
TCAGTGACGGTGGGTCTGGGATAGATTTCACACTCAGGATCATCAGTGTGGAGACTGCAGAAGCTGCA
CATACCTCTGTG

>pVκ28

GATATTGTGATGACCCAGACTCCACTCTCCCTGCCTGTTGTCCCTGGAGAGCTGGCCTCCATCTCATGC
AGGTGTAGTCAGAGCCTCCTACATAGTAATGGAAACACCTATTTGCACTGGTTCCTGCAGAAGCCAGG
CCAGTCTCCAAGGCGTCTGACCTATAGGGTGTCCAACCGGAACTCTGGGGTCCCAGACAGGTTCATTG
GCAGCGGGTCAGGGACAGATTTTACACTTGGAGGCTGAAGATGTTGGAGTTTTTATTGCTCCCAAGG
TACACA

>pVκ29

AAACATCTTGACCCAGTCTCCGGCTTCCATGGTTGTTTCCCAAGGGGAGAGGGTCACCGTCACCTGCA
GGATCAGCGCAAGTATAAGTACCAGCTACTTAACTGGTAACAACAGAAGCCAGAAGTCCGTCTAAAG
CTCCTTGTTTATGGCACATCAAACTTGGCCTCCAGGGTTTCAGCCAGCTTCAGCGGCAGCGCATCTGGG
GCCTCTTATTCTCTCACCATCAGCTGTGTGTAGGTTGAAGATGCTGCAATCTACTCTGGT

>pVκ30

GACACTGTGCTGACCCAGTCTCCACCCTCCTTGACCAGGTCTCCAGGGCAGAGGGCCACCATCTCCTG
CAGGACAAGTGAGAGTATCAATAATTATTTTGGCATTATTAGCCCTATTAACTGGTATCAACAGAAAC
CAGGACAGCCTCCTAAACTCCTGATCTCTGCAGCACCCAGTTGAGAGTCTGGGGTCCCAGCCAAGGTC
AGTGGCAGTGGGTCTGGGACTGATTTCACCCTCACAATTCATCCTGTGGAGGCCGACGCTGCTGCAAA
CTACTACTGC

>Vκ31

CACATTGTGATGACCCAGTCTCCAGGTTCCCTGGCTGTGTCTGTAGAAGACAGTGTCACCATCAACTG
CAAGTCTAGCCAGAGTCTTTTCGGTAGCTCCAACCAGAAGAATTACTTAGCCTGGTACCAGCAGAAAC
CAGGCCAGGCTCCTAAGCTGCTCATCTACTGGGCATCCAGTAGAGCATCTGGGGTTTCTGACCAATTC
AGTGGCAGTGGGTCTGGAACAGACTTCACTCTCACCATCAGCAGCCTCCAGGCTGAAGATGTGGCAGT
TTATTGCTGT

>pVκ32

GACATTGTGCTGCCCCTATTTGCAACTTTCAGTCAAGGCTTCAGGAAAGGGAGTCACCATCAGCTGCA
AGTCCAAACAAGGCATCAGTAGCTATTTGAACTGGCACCAATAAAAAACAACAGGCTCCCGAGCTTCT
CATTTACATTTCTTCTGTACCCTTGACTCCCTGCCTGGTTTAGTGATGGTGGGTCTGGGATAGATTTCAC
CCACACAATCAGCAGCCTGGAGGCTGAAGATGCATCGCATATTATTATTGTC

>pVκ33

GACATTGTGATGACCCAGAGTCCACTCTCCCTGCCCGTCACCCCAGAAGAGCCGGCCTCCACCTCCTG
CAGGTCTAGTCAGAGCCTTCTCCATAGTAATGGATACACCTATTTGGGTTAGTACCTGCAGAAGCCAG
GCCAGTCTCCACAGCTGCTGATCTATTTGGTTTCCAGCCGGGCCTCTGGAGTCTCAGACAGATTCGGTG

GCAGTGGGTCAAAGACAGATTTCACCCTTAAAATCAGCAGAGTAGAGCCTGAGGATGGTGGAGTTTA
TTACTGC
>pVκ34
CCAACAGAAACCAGGACAACCACCTCAACTGCTCTCACATGAAATTTCAACCCTAACCCCTGGGAACC
CATCCACTTCAGTAGCTGTGGGTCTGGGACAGTTTCATCCCTATAATTACTGGAGTGGAGGCTAGAAA
TGCTACATATACTCCTGTG
>Vκ35
GATATTGTGATGACCCAGACTCCCCTCTGCTTGGCCGTCACCCTGGGAGAGCCAGTTTCCATCTCCTGC
AGGTCTAGTCAGAGCCTCCTCCGTAGTGATGACTACACCTATTTGGATTGGTACCTGCAGAAGCCAGG
CCAGTCTCCACGGCTGCTGATCTATGAGGTTTCCAAGCTGGTCTCTGGAGTCTCAGACAGGTTCAGTG
GCAGTGGGTCAGGGACAGATTTCACCCTTCAAATCAGCAGAGTGGAGGCTGAGGATGTTGGAGTTTAT
TACTGC
>pVκ36
CATGTTCTACTGACCCAGTCGCCAGCTTCCATCTCTGCACCTTCAAGAGAAAATGGCACCATGATGTG
CAAGTCCAGGGAGACCCTTCTACCCTGCAGTGGACACATGGCCACAGGTGTTTCCAAAAGATACCAGG
GCAACCACCTCAATTCCTCTTACATAACATTTCAGCCCTAATCCCTGGGCACCCACCCAGTTCAGTGGC
TGTGGGTCTGGGAAAGTTTCATCACCGTAATTACTGGAGTGGAGGCTAGAAATGGTACACGTACTCCT
GTG
>pVκ37
GGTGTTGTAATGATCCAGACCCCACTCTCCCTGTCCATCAGTCCTGGAGAACTGGCTTCCATCTCCTGT
AGGTTAGTCAAAGACCCCTGCATACTGATGGTTATACCTCATTTTATGGGTTCTAACAGAAGCTTGGCC
AGTCTCCACAGAAGCTGATCCATGAAATTTCCAACCTTAGCCCTGGGGTCTTAGACAGGTTCAGTGGC
AGTGGGTCAGGGACAGATTTCACACTTAAAATTAGCAGGGTGGCAGCTGAGGATGTTGGAGTTTGTTA
CTGC
>pVκ38
CACATTGTGCTCACCCAAGCTCCAGTGTCCTTGGCCTTCTCTCTGGGAAAGAGAGTCTCTATCAGCAGC
AGGTCCAGTGATAGCCTCCTCTACTCAGATGGGAAGTTTTTCTTATTCTGTTACCAGCACAGATCTGGG
CAGCTCCACAGACTCTCATTTCCCTCACCGCCCCCCGAGTTAACAAGATGCCCACCAGGATCAGTAGC
AGCTGGTCTGGGACAGATTTCACTCTCACCATCAGAAGAGTGGAGTCTGAGAAATTTGCAGTCGAGGA
CTGT
>Vκ39
CACATTGTGATGACCCAGTCTCCAGTCTCCCTGGCTGTGTCTGTAGGAGACAGGGTCACCATCAACTG
CAAGTCCAGCCAGAGTCTTTTATACAGCTCCAAACAGAAGAATTACTTAGCCTGGTACCAGCAGAAAC
CAGGCCAGGCTCCTAAGCTGCTCATCTACTGGGCATCCACCCGGGCCACAGGGGTCCCTGACCGATTC
AGTGGCAGTGGGTCTGGAACAGATTTCACTGTCACCATCAGCAGCCTCCAGGCTGAAGATGTGGCACT
TTATTACCGT
>pVκ40
GAAGTCCAGATGATCCAGTCTCCCTCCTCACTGTCTGTATCCCTAGGTATCAGAGTCACCGTCACTTGC
CGGGCATGTTAGGGCATCAGCAAGTGGTTATCCTGGTCTCAGTAGAAACCAAGGAAAGTTCCTAAGCT
CCTGATCGATGTTGCATCCAGTTTGCACACCGGGGTCCCTTCGCAGATTGCGGTTCAGCGGCAGTGGA
TCTGGCACAGATTTCAGTCTCACCATCAGCAGCCTGGAGCCTGAATATGTTGCAACTTA
>pVκ41
GATATTATGATGACTCAGATTCCACTCTCCCTGTCTGTCATCCCTGGGAGCCGGCCTCCATCTCCTGTA
GGTCTAGTCAGAGTCTCCTACATAGTAATGGATACATCTATTTGCAATGGTTCCCACAGAACCCAGGC

CAGTCTCCAAGGCACCTGATCTATAGGGTATCCAATCATAATTCTGGGGTCCCAGACAGGTTCAGTGG
CAGTGGGTCAGGGACAGATTTCACACTCAAAATCAGCAGGGTGGAAGCTGAGGATGTTGGAGTTTATT
ACTGC

>pVκ42

GACATTGTGGTGACCCAGTCTCCAGCCTCTCTGTCTGGAGCTCCTGGAGAGAGTCACCTTCATGGCCA
GTGAAAATCTCAAGAACACTGGAGGGAAAACCTATCCAACAGAAAAACCGAAAACCTAAGCCCCTCT
TTTACCCACTTTCCAAGATACACTATGGGATCCCAGCCCAGTTCAGTGGCAGTAGCTCTGACAGAAAT
TTCACCAGCACCATCAGTAACCTAGGGGCTGAAGATGCCACTAGGTACCACTGT

>pVκ43

CAGAGCGTCCTGACTCACAGTCCATCTTCCTAGTCTGTGTCCCACAGACACAAAGTCACAAGCACCTG
CAGGGTGAGCAAGAGTGTGACTGACAAAAATAGCATCAGGGTACACCGGTATCAACAGAAACCTAGA
AAACCTGCTCAGTGTCTCATATATAAAGCCTCCAATCCTCAGCCAGGGGTCCCAGCCCTATTCTCAGG
CAGTGGGTCTGAGAGAGTTCGCTCTGACCATAAGCAAAATGGAGACAGATGATACTCAGATTACTACT
GT

>Vκ44

GACATTGTGCTGACCCAGTCTCCAGCCTCCTTGACGGTGTCTCCAGGTCACAGCGCCACAATTTCCTGC
AGGGCCAGTGAGAGTATTGGTTCTTTTGGCATGTATTATAACCTTGTCATACATAACCTTCACTGGTAT
CAACAGAAACCAGGACAGCGTCCTAAACTCCTGATCTCTTCAGCCTCCAATCTAGTGTCTGGGGTCCC
AGCCAGGTTCAGTGGCAGTGGGTCTGGGACTGATTTCACCCTCACAATTAATCCTGTGGAGGAGGCCG
ATGCTGCAAACTACTACTGC

>pVκ45

CACATTGTGCTCACCCAAGCTCCAGTGTCCTTGGCCTTCTCTCTGGGAAAGAGAGTCTCTATCAGCAGC
AGGTCCAGTGATAGCCTCCTCTACTCAGATGGGAAGTTTTTCTTATTCTGTTACCAGCACAGATCTGGG
CAGCTCCACAGACTCTCATTTCCCTCACCGCCCCCCGAGTTAACAAGATGCCCACCAGGATCAGTAGC
AGCTGGTCTGGGACAGATTTCACTCTCACCATCAGAAGAGTGGAGTCTGAGAAATTTGCAGTCGAGGA
CTGTC

>pVκ46

GACATCGTGATGATCCAGTTTTCAAGGTTCCTGGCTGTTTCTGCATGAGACAGGTTCACCATCAATTGC
AAGTCCAGACAGAGCATCAGTAACGACTTAGCCTTGTATCAGCAGAAACCAGGACAGGGTCCTAAGA
TGATGATCTCCTTGCCTCCATCCAGACACCTAGGGGCCCTGACTGACTCAGTGGCACTGGATCTGCAA
CAGATTTCGTGCTCACTGTCAGCAACCTCCAGGCTGAAGAAAGGGCCAACTTTTACTGTC

>pVκ47

ATCATGCTCTCTCAGGCTACAGAGTCCTTGCCTTTCTCCCCAGGAGAGAGAGTCTCCATCAGCTGCAG
GGCCAGGAAAAGCCCCCCCCTACATGGACATGAAGCACTACATAGGGTGGTACCTGTATAGACCTGG
GCAAGCCCCCAAGGCCCTCATTTAC

>pVκ48

GACATCATGACGACCCAGTCTCCAGGCTCCTTGGCAGTGTCTCTAGGATAGAGTCGAGATGAAGTGCA
AGACCAGTCAGAGTGTTTACTACTACCTACTTAGCCTGGTAGCAGAAAAATCCAGGCCAGGCTCCTAA
GCTGCTCACCTACTTGGCATCCACTCGGGTTACAGGGGTCCCTGACCGATTCAGTGGCAGTAGGTCTG
GAACAGATTTCACACTCACCATCAGCAGCCTCCAGGCTGAAGATGTGGCAGTTTATTACTGT

>Vκ49

GACATCGTGATGACCCAGTCTCTAGGCTCCTTGGCAGTGTCTCCAGGACAGAGGGTCACCATTAGCTG
CAAGGCCAGTCAGAGTGTTAGCAACTACTTAGACTGGTACCAGCAAAAACCAGGACAGGCTCCTATG
CTGCTTATCTATGCAGCATCCAGCAGAGCATCTGGGGTCCCCGACAGATTCAGTGGCGGTGGATCTGG

GACAGATTTCTCTCTCACCATCAGCAGCCTGCAGGCTGAAGATGTGGCAGTTTATCACTGT
>pVκ50
GACATTGTGATGATCCAGTCTTCGCTGTTCCTGGCTGTTTCTGCAGGAGAGAGGTTCGCTAACAACTGC
AAGTCAAGCCAGAGCATTAGCAACTACTTACCCTGGTACCAGCAGAATCTAGGACAGGATCCTAGGCT
GCTCATCTCCTGGGCATCCACCCAGGCACCTGAGGTCTCTGAATGACTCAGTGGCAGTGGATCTGTGA
CAGATTTCACTCTCACTATCAGCAGCAGCCTTCAGGCTGAAGCTGTGGCTGACTTTTAC
>Vκ51-ORF
GCTTCAGAGTCCTTGCCTATCTCCCCAGGAGAGAGAGTATCCATCAGCTGCAGGACCAGGGAAAGCCT
CCTCTACATGGATATCAAGCATAACTTAGTGTGGTACCAGCACAGATCTGGGCTGGCCCCAAAGGACC
TCATTTGTCATGCTTCCACTGAAGTTAACAGCATGCTCACCCAATGGTTCAGTGGTAGTGCGTCTGACA
GACTTCATTCTTATCATCAGCAGTATGGATGCTGGGCGCCGGCCTGGTGCAGCAGT
>pVκ52
GACATCGTGATGACCCAGTCTCCAGGGTCCTTGGCTGTGTCTCTAGGGCAGAGGGTCAACATTAAGGG
CAAGGCCAGTCAGAGTATCAACAACAAGTTAGATTGGTACCAGCAAAAACCAGAACAGGCTGCTAAG
CTGCTGATCAATGCAGCATCCAGCAGAGCATCTGGGGTCCCCTGACCGATTCAGCAGCAGCGGATCTG
GGACAGATTTCACACTCACCATCAGCAGCCTCCAGGCTGAAGATGTGGCAGTTTATTACTG
>pVκ53
GACATTGTGATGGTCCAGTCTTCAGGGTTCCTGGCTGTTTCTACAGGAGAGAGATTCACCGTCAAGTG
CAAGTCCAGCCAGAGCATTAGAAACTAATTAGCCTGGTACCAGCAGAAACAAGGAAAGGCTTGGAAA
CTGCTCACCTCCTGGGCATCCACCCAGGCACCTGGGGTCCCTGACTGACTCAGTGGCAATGGACCTGT
GACAGATTTTCACTTTCACTGTCAGCAACCTCCAGGCTGAAGATGTGGCTGACTTTTACTG
>Vκ54
GACATCATGATGACCCAGTCTCCAGACTCCTTGGCAGTGTCTCTAGGAGAGAGGGTCGACATGAAGTG
CACGGCCAGTCAGAGTGTTTACCACTACTTAGCCTGGTACCAGCAAAAACCAGGACAGGCCCCTAAGC
CCCTCATCTACTCAGCATCTACCAGACCATCTGGGGTCCCTGACCGATTCAGTGGCAGTGGATCTGGG
ATAGATTTCACTCTCACCATCAGCAACTTCCAGGCTGAAGATGCGGCAGTTTATTGCTGT
>Vκ55
GACATCGTGATGACGCAGTCTCCAGACTCCTTGGCAGTGTCTCTAGGACAGAGAGTCGAGATGAAGTG
CAAGGCCAGTCAAAGTGTTAGCAGCTACTTAGCCTGGTACCAGCAAAAACCAGGACAGGCTCCTAAG
CTGCTCATCTACAAAGCATCCAACAGAGCATCTGGCGTCCCTGATCGATTCAGTGGCAGTGGATCTGG
GACAGATTTCACTCTCACCATCAGCAGCCTCCAAGCTGAAGATGTGGCAGTTTATTACTGT
>pVκ56
GACAGCATAATGATCCAGTCTTCCCGGTTGCTGGCTATTTCTGCAGGAGACAGGTTCACCATCAACTG
CAAGTCCGGCTGGAGCATTAGCAACTACTTAGCCTGGTACCAGTAGAAACCGGGACAGACTCCAAAG
CTGCTCACCTCTTGGGCATCCCACCCAGGTACCTGAGGTCCCTGACTGACTCAGTGGCAGCGGATCTG
TGACAGATTTCACTCTCACCGTCAGCGGTCTCCAGGCTGAAGATGTGGCCGACTTTTA
>pVκ57
GACATCGTGATGACCCAGTCTCCAGATTCCTTGGCTGTGTGTCCAAGACTGATGGTCACCCTTGGCTGC
AGGGCTAGTGAGAGTGTTAGCAACTTCTTAGTCCAGTACCAGCAAAAACCAGGACAGGCTCCTAAGCT
GCTAATGTATGCAGCATCCACCAGGTCATCTGGGTTTCCTGACTGATTCAGTGGCAGTTAATCTGGGAT
AGATTTCACTCTCACACTCGGCAGCCTCCAGGCAGAAGATATGGCTATTGATATCTGT
>pVκ58
TAGATTGTGCTCTCTCAGGCTGCAGAGTCCTTGCCTGTCTCCCCAGGAAAGAGAGTCTCTATCAGCTGC
AGAGCCACGGAAAGCCTCCTCTATGTGGATGTCAGGCATTACTTAGCGTGGTACCAGTACAGACCTGG

GTAGCCCCCCAAGGCCCTCATTTACCATGCTTCCACTCGAGTTAACAGCATGCTCACCGGGTTCAGTG
GCAGTGGGTCTGGGACAGACTTCACTCCTATCATCAGCAGCCTGGAAGCTAAAGATAGTACAACTAAT
TACTGT

>Vκ59

GACATCGTGATGACCCAGTCTCCAGGTTCCTTGGCAGCATCTCTAGGACAGAGAGTCGAGATGAAGTG
CAAGGCCAGTCAGAGTGCTAGCAGCTACTTAGCTTGGTACCAGCAAAAACCAGGACAGGCTCCTAAG
CAGCTCATCTACAGTGCATCCAGCAGAGCGTCTGGGGTCCCCGACCGATTCAGTGGCAGTGGATCTGG
GACAGATTTCACTCTCACCATCAGCAGCCTCCAGGCTGAAGATCTGGCCATTTATTACTGT

>Vκ60

GACATCGTGATGACCCAGTCTCCAGGTTCCTTGGCAGCATCTCTAGGACAGAGAGTCGAGATGAAGTG
CAAGGCCAGTCAGAGTGCTAGCAGCTACTTAGCTTGGTACCAGCAAAAACCAGGACAGGCTCCTAAG
CAGCTCATCTACAGTGCATCCAGCAGAGCGTCTGGGGTCCCCGACCGATTCAGTGGCAGTGGATCTGG
GACAGATTTCACTCTCACCATCAGCAGCCTCCAGGCTGAAGATGTGGCACTTTACTACTGT

>Vκ61

GACATCGTGATGACCCAGTCTCCAGACTCCTTGTCTGTGTCTCCAGGACAAAGGGTCGACATGAAGTG
CAGGGCCAGTCAGAGTGTTAGCAATGAGTTATCCTGGTACCAGCAAAAACCAGGACAGGCTCCTAAG
CTGCTGATCTATGCAGCATCCAACAGAGCATCTGTGGTCCCTGACCGATTCAGTGGCAGTGGATCTGG
GACAGATTTCACTCTCACCATCAGCAGCCTCCAGGCTGAAGATGTGGCCGTTTATTACTGT

>pVκ62

GACATTGTGATGATCCAGTCTTCGCTGTTCCTGGCTGTTTTTCTGCAGGAGAGAGGTTCGCTAACAACT
GCAAGTCAAGCCAGAGCATTAGCAACTACTTACCCTGGTACCAGCAGAATCTAGGACAGGATCCTAG
GCTGCTCATCTCCTGGGCATCCACCCAGGCACCTGAGGTCTCTGAATGACTCAGTGGCAGTGGATCTG
TGACAGATTTCACTCTCACTATCAGCAGCCTTCAGGCTGAAGCTGTGGCTGACTTTTACTGT

>Vκ63-ORF

GCTTCAGAGTCCTTGCCTATCTCCCCAGGAGAGAGAGTATCCATCAGCTGCAGGACCAGGGAAAGCCT
CCTCTACATGGATATCAAGCATAACTTAGTGTGGTACCAGCACAGATCTGGGCTGGCCCCAAAGGACC
TCATTTGTCATGCTTCCACTGAAGTTAACAGCATGCTCACCCAATGGTTCAGTGGTAGTGCGTCTGACA
GACTTCATTCTTATCATCAGCAGTATGGATGCTGGGCGCCGGCCTGGTGCAGCAGT

>pVκ64

GACATCGTGATGACCCAGTCTCCAGGGTCCTTGGCTGTGTCTCTAGGGCAGAGGGTCAACATTAAGGG
CAAGGCCAGTCAGAGTATCAACAACAAGTTAGATTGGTACCAGCAAAAACCAGAACAGGCTGCTAAG
CTGCTGATCAATGCAGCATCCAGCAGAGCATCTGGGGTCCCCTGACCGATTCAGCAGCAGCGGATCTG
GGACAGATTTCACACTCACCATCAGCAGCCTCCAGGCTGAAGATGTGGCAGTTTATTACTG

>pVκ65

GACATTGTGATGATCCAGGCTTCTCGGCTCTTGGCTGTTTCCGCAGGAGAGAGATTCACCATCAACCG
TAAAGTCCAGCCAGAGCATTAGCAACTACCTATCCTGCTACCAGCAGAAACCAGGACAGGGTCCTAA
GCTGCTCATCTCCTGGGCATCCATTCAGGCACATGGGGTCCCTGACTGACTCAGTGGCAGTGGATCTG
CCACTGATCTCACTCTCACCATCAGCAGCCTCCTCTGCTGCTGATGTAGCCAACTTTTA

>Vκ66-ORF

GGTAGATCATGCTCTCACGCTGCAGAGTCCTTGCTTGTCTCTACAGGAGAGAGAGTCTCCATCAGCAG
CAGGGCCATGGAAAGCCTCTTCTGCATAGATGTGAGGCATTGCTTAGCAGGATATCACCACCGACCTG
GGCAGGCCCCCAGGGCCCTCACTAACCACGATTCTACTCAAGTTAACAGCATGCTTACCCAGTTCAGT
GGCAGTGGGTCTGGGACAGACTTCATTCTTATCATCAGCAGCCTGGAAGGTAAAGATAATTACTCTTT
TCCAACC

>Vκ67
GACATCGTGATGACCCAGTCTCCAGAGTCCTTGGCAGAGTCTGTAGGACAGAGGGTCGAGATGAAGT
GCAAGGCCAGTCAGAGTGTTAGCAGCTACTTAGCCTGGTACCAGCACAAACCAGGACAGGCTCCTAA
GCTGCTCATCTACAGAGCATCCAGCAGAGCATCTGGGGTCCCTGACCGATTCAGTGGCAGTGGATCTG
GGACAGATTTCACTCTCACCATCAGCAGCCTCCAGGCTGAAGATGTGGCCGTTTATTACTGT
>pVκ68
GACATCGTGATGACCCAGTCTCCAGGCTCCTTGGCAGTGTCTCTAGGACAGAGAGTCTAGATGAAGTG
CAAGGCCAGTCAGAGTGTTAGCAACTACTTAGACTGGTACCAGCAAAAACCAGTAAAGGCTCCTAAG
CTGCTCATCTACAGAGCATCCAGCAGAGCATCTGGGGTCCCCGACCGATTCAGCGGCAGTGGATCTGG
GACAGATTTCACTCTCACCATCAGCAGCCTCCAGGCTGAAGATGTGGCAGTTTACTACTGT
>pVκ69
GACATCATGATGATCCAGGTTTCCCAGTTCTTGGCTGTTTCTGAAAGAGAGAGATTCACCATCAACTGT
TGAGTCCAGCCAGAGCATTAGCAACTGCTTAGCCTGCTACCAGAAGAAACCAGGACAGAATCCTAAG
CTGCTTAGCTCCTCAGCATCCACCCTGGCAACTGGGGTCCCTGACTGACTCAGTGGCAGTGGATCTGC
AACTGATTTCACTCTCACCACCAGCAGCCTGTAGGCTGAAGATGTAGCCAAGTTTTACTGT
>pVκ70
TACATCATGCTCACTCAGGCCGCAGAGTCTTTGCCAGTCTCCCCAGGAGAGAGTCTCCATCAGCTGCA
GAGCGACGGAAAGCCTCCTCTACGTGGATGTGAAGCATTGCTTAGCTTGGTGCCAGCACAGACCTGGG
CAGCCCCCCAAAGCCCTCATTTACAATGCTTCCACTCCAATTAACAGCGTGCTCACCCGGTTCAGTGGC
AGTGGGTCTGGGACAGACTTCATTCTTATCATCAGCAGCCTGGAAGGTAAAGATAATTAGTTTTCCAA
CCTC
>Vκ71
GACATCGTGATGACCCAGTCTCCAGGCTCCTTGGCGGCGTCTCTAGGACAGAGAGTCGAGATGAAGTG
CAAGGCCAGTCAGAGTGTTAGCAGCAGCTTGGACTGGTACCAGCACAAACCAGGACAGGCTCCTAAG
CTGCTCATCTACAGAGCATCCAGCAGAGCATCTGGGGTCCCTGACCGATTCAGTGGCAGTGGATCTGG
GACAGATTTCACTCTCACCATCAGCAGCCTCCAGGCTGAAGATGTGGCAGTTTATTACTGT
>Vκ72
GACATCGTGATGACCCAGTCTCCAGGCTCCTTGGCAGCGTCTCTAGGACAGAGAGTCGAGATGAAGTG
CAAGGCCAGTCAGAGTGTTAGCAGCAGCTTGGACTGGTACCAGCACAAACCAGGACAGGCTCCTAAG
CTGCTCATCTACAGAGCATCCAGCAGAGCATCTGGGGTCCCTGACCGATTCAGTGGCAGTGGATCTGG
GACAGATTTCACTCTCACCATCAGCAGCCTCCAGTCTGAAGATGTGGCTGTTTATTACTGT
