# Supplementary material for: Comparative analysis of the organization and complexity of immunoglobulin light chain loci in equids
Source: J Anim Sci. 2026 Jan 9;104:skag001. doi: 10.1093/jas/skag001 (PMC12906693; doi:10.1093/jas/skag001)
Supplement: skag001_Supplementary_Data [file skag001_supplementary_data.zip › Supplementary_data.zip!/Supplementary Figure.docx]

**Supplementary figures**

**Comparative Analysis of the Organization and Complexity of Immunoglobulin Light Chain Loci in Equids**

Yanbo Qiu^a^, Yichen Lei^b^, Xiaohua Yi^b^, Xiaoqin Tang^b^, Beibei Zhang^a^, Shuhui Wang^b^*, Xiuzhu Sun^a^*

*a College of Grassland Agriculture, Northwest A&F University, Yangling, China*

*b College of Animal Science and Technology, Northwest A&F University, Yangling, China*

*Correspondence: Shuhui Wang	E-mail: wangshuhui252@163.com

*Correspondence: Xiuzhu Sun E-mail: [sunxiuzhu@nwafu.edu.cn](mailto:sunxiuzhu@nwafu.edu.cn)

Supplementary Figure 1 Phylogenetic tree of 254 Vλ (A) and 72 Vκ (B) genes in donkeys

Supplementary Figure 2 Subgroup classification of potentially functional V genes utilized by horses and donkeys

A~D: Significance analysis of subgroups attributed to potentially functional Vλ (A) and Vκ (B) genes utilized by donkeys; Significance analysis of subgroups attributed to potentially functional Vλ (C) and Vκ (D) genes utilized by horses.

Supplementary Figure 3 Somatic hypermutation (SHM) analysis of horses V gene (FR1-FR3)

A~F: GZ and NQ Horse Vλ (A) and Vκ (B) base mutation position; Vλ (C) and Vκ (D) total mutation rate; Vλ (E) and Vκ (F) transitions and transversions occurrence frequency.

Supplementary Figure 4 Frequency of Vλ gene transitions and transversions occurring in the donkeys and horses IGLV3 subgroup
