# Supplementary material for: Comparative analysis of the organization and complexity of immunoglobulin light chain loci in equids
Source: J Anim Sci. 2026 Jan 9;104:skag001. doi: 10.1093/jas/skag001 (PMC12906693; doi:10.1093/jas/skag001)
Supplement: skag001_Supplementary_Data [file skag001_supplementary_data.zip › Supplementary_data.zip!/Supplementary Table.pdf]

Supplementary tables

Comparative Analysis of the Organization and Complexity of Immunoglobulin Light Chain Loci in Equids

Yanbo Qiu[a], Yichen Lei[b], Xiaohua Yi[b], Xiaoqin Tang[b], Beibei Zhang[a], Shuhui Wang[b]*, Xiuzhu Sun[a]*

a College of Grassland Agriculture, Northwest A&F University, Yangling, China

b College of Animal Science and Technology, Northwest A&F University, Yangling, China

*Correspondence: Shuhui Wang E-mail: wangshuhui252@163.com

*Correspondence: Xiuzhu Sun E-mail: sunxiuzhu@nwafu.edu.cn

Supplementary Table 1 Sequencing datasets generated and processed by PE300 in this study

Sample	Reads (×2)	Merged reads	Productive rearrangement reads	Unique CDR3s	Reads (×2)	Merged reads	Productive rearrangement reads	Unique CDR3s
	IGλ				IGκ			
GZ Donkey-1	127137	125846	8247	4538	401227	291835	42526	5646
GZ Donkey-2	101898	98482	11715	5403	387366	300067	48461	6421
GZ Donkey-3	212697	211768	22528	11673	39321	30863	11682	2249
JM Donkey-1	82419	79822	14813	4816	59617	45840	15270	2677
JM Donkey-2	83695	81659	17227	4668	67391	54592	16459	2829
JM Donkey-3	61542	59891	18539	13562	156278	121067	16400	2736
NS Donkey-1	215213	207654	37500	18616	110443	83243	13998	2712
NS Donkey-2	200475	199767	78095	24600	68892	54953	15615	2758
NS Donkey-3	72860	70829	21790	15293	70382	49809	14362	2688
GZ Horse-1	76219	75199	22864	10382	802483	790721	8205	2516
GZ Horse-2	323892	317816	16832	9816	98418	98257	59997	7831
GZ Horse-3	347388	341436	45499	23254	471478	465719	45798	5426
NQ Horse-1	48259	47545	30223	6623	89924	89707	53497	5858
NQ Horse-2	262945	258878	64488	18329	110908	110678	54141	5042
NQ Horse-3	294185	291599	64199	24611	191287	187010	14259	4216

Supplementary Table 2 V(D)J recombination diversity index analysis

A

Index	Sample	GZ Donkey	JM Donkey	NS Donkey
		(Mean ± SEM)		
lambda	Richness	90±2.65[a]	93.33±0.67[ab]	117±2.65[b]
	Shannon-Wiener Index	3.59±0.1	3.59±0.03	3.62±0.07
	Pielou Evenness Index	0.8±0.03	0.79±0.01	0.76±0.01
kappa	Richness	43.33±2.33	39.67±0.33	39.33±0.88
	Shannon-Wiener Index	1.63±0.07	1.58±0.01	1.62±0.02
	Pielou Evenness Index	0.43±0.02	0.43±0	0.44±0

B

Index	Sample	GZ Horse	NQ Horse
		(Mean ± SEM)	
lambda	Richness	96±4.58	101.33±5.67
	Shannon-Wiener Index	3.59±0.11	3.42±0.21
	Pielou Evenness Index	0.79±0.02	0.74±0.04
kappa	Richness	49±0	48.33±1.76
	Shannon-Wiener Index	1.99±0.15	1.41±0.21
	Pielou Evenness Index	0.51±0.04	0.36±0.05

Note: Within the same row, groups marked with different lowercase letters are considered statistically significant (P<0.05), while identical letters indicate no significant difference (P>0.05).

Supplementary Table 3 Statistics of inserted nucleotides (nt) in the region contributing to junction diversity in horse and donkey IGλ (A) and IGκ (B)

A

	3'V-Region	P1	N	P2	5'J-Region
GZ Horse	15.02±0.02[a]	0±0	14.74±0.03[a]	0.09±0[a]	7.69±0.01[a]
NQ Horse	14.54±0.02[b]	0±0	15.26±0.02[b]	0.07±0[b]	7.19±0.01[b]
GZ Donkey	14.09±0.03[c]	0±0	15.91±0.04[c]	0.08±0[c]	7.31±0.01[c]
JM Donkey	14.32±0.03[d]	0±0	15.30±0.03[b]	0.09±0[a]	7.32±0.01[c]
NS Donkey	15.70±0.02[e]	0±0	13.70±0.02[d]	0.12±0[d]	7.86±0.01[d]

B

	3'V-Region	P1	N	P2	5'J-Region
GZ Horse	22.80±0.01	0.01±0	1.57±0.01	0.03±0	8.62±0
NQ Horse	22.15±0.01	0±0	2.13±0.01	0.03±0	8.74±0
GZ Donkey	22.62±0.01	0.01±0	1.84±0.01	0.03±0	8.52±0
JM Donkey	22.44±0.02	0±0	1.99±0.02	0.03±0	8.60±0.01
NS Donkey	22.39±0.02	0±0	2.02±0.02	0.03±0	8.60±0.01

Note: Within the same column, groups marked with different lowercase letters are considered statistically significant ($P<0.05$), while identical letters indicate no significant difference ($P>0.05$).

Supplementary Table 4 Percentage (%) of cysteine content in CDR3λ (A) and CDR3κ (B)

A

Sample \ Number of Cysteines	0	1	2	3
GZ Donkey-1	92.39	7.49	0.12	0.00
GZ Donkey-2	93.53	6.24	0.23	0.00
GZ Donkey-3	92.70	7.08	0.20	0.02
JM Donkey-1	92.16	7.64	0.16	0.03
JM Donkey-2	91.61	8.24	0.16	0.00
JM Donkey-3	95.66	4.18	0.15	0.01
NS Donkey-1	96.64	3.20	0.15	0.01
NS Donkey-2	96.31	3.22	0.45	0.02
NS Donkey-3	95.20	4.65	0.15	0.00
GZ Horse-1	94.67	5.11	0.21	0.00
GZ Horse-2	94.59	5.21	0.19	0.01
GZ Horse-3	95.35	4.44	0.21	0.00
NQ Horse-1	97.17	2.72	0.11	0.00
NQ Horse-2	91.80	8.10	0.10	0.00
NQ Horse-3	94.12	5.76	0.11	0.00

B

Sample \ Number of Cysteines	0	1	2
GZ Donkey-1	98.36	1.64	0.00
GZ Donkey-2	98.29	1.70	0.01
GZ Donkey-3	98.06	1.94	0.00
JM Donkey-1	98.07	1.93	0.00
JM Donkey-2	98.17	1.82	0.01
JM Donkey-3	98.29	1.71	0.00
NS Donkey-1	97.91	2.09	0.00
NS Donkey-2	98.14	1.83	0.03
NS Donkey-3	98.17	1.83	0.00
GZ Horse-1	98.15	1.85	0.00
GZ Horse-2	99.56	0.44	0.00
GZ Horse-3	98.37	1.62	0.01
NQ Horse-1	99.68	0.32	0.00
NQ Horse-2	98.26	1.74	0.00
NQ Horse-3	97.76	2.24	0.00

Supplementary Table 5 Correlation analysis between CDR3λ and CDR3κ length and number of cysteine residues in horses and donkeys

Sample	CDR3λ		CDR3κ	
	Correlation coefficient	*P* Value	Correlation coefficient	*P* Value
GZ Donkey	-0.088579374	$P<0.01$	-0.020913005	$P<0.01$
JM Donkey	-0.075132014	$P<0.01$	-0.022105707	$P<0.01$
NS Donkey	0.028109897	$P<0.01$	-0.018775537	$P<0.01$
GZ Horse	0.002801739	$P>0.05$	-0.017003622	$P<0.01$
NQ Horse	-0.134805698	$P<0.01$	-0.017359084	$P<0.01$
